# Implementation factors of group antenatal care in low-resource settings in Tanzania: A qualitative study using the Consolidated Framework for Implementation Research

**DOI:** 10.1371/journal.pgph.0006221

**Published:** 2026-09-18

**Authors:** Alen Kinyina, Augustino Hellar, Raymond Bandio, Hamid Mandali, Ahmad Makuwani, Phineas Sospeter, Jennie Jaribu, Isaac Lyatuu, Yusuph Kulindwa, Frank Phiri, Ntuli Kapologwe, Sarah Chamos, Cyprian Mtani, Wilfred Kafuku, Omari Sukari, Abubakari Munga, Husna Athumani, James Hellar

**Affiliations:** 1 Prime Health Initiative Tanzania (PHIT), Dar es salaam, Tanzania; 2 Ifakara Health Institute (IHI), Dar es salaam, Tanzania; 3 Ministry of Health, Dodoma, Tanzania; 4 East, Central, and Southern Africa Health Community (ECSA-HC), Arusha, Tanzania; 5 Ministry of Foreign Affairs and East African Co-operation, Dodoma, Tanzania; 6 Geita Regional Health Management Team (RHMT), Geita, Tanzania; University of Washington Seattle Campus: University of Washington, UNITED STATES OF AMERICA

## Abstract

Antenatal care (ANC) is essential for improving maternal and newborn health, yet many women do not complete the recommended follow-up visits and often experience suboptimal quality of care and weak provider-client interactions. Group antenatal care (G-ANC) offers an alternative service delivery model that combines clinical assessments with group-based education, facilitated discussion, and peer support for women with similar gestational age (GA). This study examined the facilitators and barriers to G-ANC implementation in low-resource settings in Tanzania using the Consolidated Framework for Implementation Research (CFIR).A qualitative study was conducted in the Geita Region from June to November 2024. Data were collected through Focus Group Discussions (FGDs) with pregnant and recently delivered women who participated in G-ANC. Key Informant Interviews (KIIs) included the healthcare providers (HCPs), facility leaders, and health managers. Using NVivo Version 14.0 software, we managed and analysed all interviews to explore facilitators and barriers to the implementation of G-ANC. Data were grouped and reported according to their relevance to the CFIR constructs. Implementation of G-ANC was shaped by interacting factors across all CFIR domains. Key facilitators included the perceived relative advantage of G-ANC over conventional ANC, adaptability of the model to local contexts, supportive facility leadership, effective teamwork, and peer support among women. Similarly, implementation processes such as proper scheduling of group sessions supported continuity of G-ANC services. In addition, the positive experiences of community health workers (CHWs), HCPs and pregnant women reinforced sustained implementation. Major barriers included inadequate space for meetings, workforce shortages, competing clinical demands, socioeconomic constraints and privacy concerns in group settings. Successful implementation of G-ANC in low-resource settings requires alignment between intervention design, health system capacity, and community context. Addressing multilevel barriers while strengthening identified facilitators is critical for the successful adoption of G-ANC and its integration into other maternal and newborn health services.

## Introduction

Pregnancy and childbirth continue to pose significant risks to the health and survival of both women and their infants in low-resource settings. Globally, about 260,000 women died during and following pregnancy and childbirth in 2023 alone [1]. In the same year, approximately 2.3 million newborns died due to complications arising during pregnancy, childbirth, and the early postnatal period [2]. Despite a 40% decline in the maternal mortality ratio (MMR), from 727 to 442 deaths per 100,000 live births between 2000 and 2023, Sub-Saharan Africa (SSA) still accounts for 70% of the global burden [3]. In Tanzania, the MMR is 104 per 100,000 live births and the neonatal mortality rate is about 24 per 1,000 live births [4]. Reducing maternal and newborn mortality remains a central global development priority, as evident in its inclusion in Sustainable Development Goal (SDG) 3 [3,5].

Antenatal care (ANC) is a critical entry point for improving maternal and newborn health outcomes through early detection of complications, preventive interventions, and health education. Adherence to the World Health Organization (WHO) recommendation of a minimum of eight ANC visits improves the early identification and management of pregnancy-related complications [6,7]. Despite the implementation of multiple strategies to expand ANC coverage, service utilisation remains suboptimal, particularly in low-resource settings [7]. In Tanzania, many primary healthcare facilities face persistent challenges, including high client volumes, shortages of skilled health workers, limited infrastructure, and constrained time for meaningful provider–client interaction [8]. These constraints often result in brief, task-focused ANC services that limit opportunities for adequate interaction, clinical consultation, and psychosocial support [7–9].

Health system pressures, including workforce shortages, limited funding allocations, and changes in geopolitical health financing, have further strained maternal health services [10]. These pressures underscore the need for service delivery approaches that are both efficient and resilient, particularly in low-resource settings where health system capacity is limited. In response, the WHO has increasingly emphasised innovative and client-centred approaches to maternal health service delivery [11]. Within this policy context, Group Antenatal Care (G-ANC) has been introduced as one of the alternative models designed to reorganize ANC delivery [12–14]. Group antenatal care is a service delivery model that brings together women of similar gestational age (GA) for group-based sessions integrating individual clinical assessments, facilitated discussions, health education, and peer support [13,15]. This model aims to improve efficiency, strengthen women’s engagement in self-care, and enhance social support during pregnancy while maintaining the essential clinical components of ANC [9,14,16].

Group antenatal care follows a standardized yet flexible process to ensure quality clinical services while promoting women’s engagement and continuity of care [15,17,18]. Women are first enrolled during an initial individual ANC visit, typically in the first trimester. Eligibility is assessed, GA is confirmed, and women with similar expected date of delivery (EDD) are grouped together, usually in cohorts of 8–15 participants [15,19]. This homogeneity in GA allows discussions and clinical content to be aligned with the stages of pregnancy. Each group follows a predefined schedule of sessions, often aligned with the WHO recommendation of at least eight ANC contacts. Sessions are held at regular intervals, for example, monthly in early pregnancy and more frequently in the third trimester, with each meeting lasting approximately 90–120 minutes [15,19,20].

A typical session is organized into three integrated components. Firstly, brief individual clinical assessments are conducted within the group setting. These include peer to peer blood pressure measurement and weight monitoring. Individual clinical consultations are done to assess fundal height, foetal heart rate, and review of danger signs [19]. Secondly, facilitated group discussions form the core of the session, in which a trained midwife/nurse, or other skilled HCPs, use participatory methods rather than didactic teaching. Topics tailored to GA usually include nutrition, infection screening, birth preparedness and complication readiness, danger signs, mental health, family planning and newborn care [15,19,20].

During the sessions, women are encouraged to share experiences, ask questions, and support one another to reinforce health literacy and self-care. Time is allocated for peer interaction and social support. This informal exchange helps normalize pregnancy experiences, reduces anxiety, and has been shown to improve satisfaction and retention in care, particularly in low-resource settings where barriers to repeated facility visits are common [9,16,21,22]. Monitoring focuses not only on clinical outcomes but also on attendance, quality of facilitation, and women’s experiences of care [16].

Evidence from multiple low- and middle-income countries suggests that G-ANC can improve continuity of ANC attendance, strengthen health education delivery, and enhance provider–client interaction [9,19–21,23–25]. In Tanzania, the approach has been shown to increase ANC attendance and improve uptake of selected maternal health interventions among women participating in group care, although these outcomes have not been consistent across settings [19,24,26]. Similar to other complex health interventions, the implementation of G-ANC has varied substantially across facilities and regions, influenced by differences in infrastructure, staffing, leadership acceptance, and community context [9,15]. For example, some facilities have successfully integrated G-ANC into routine services, while others have struggled due to space constraints, competing clinical demands, and limited organizational readiness [9,19].

Such variation highlights a critical gap between intervention design and real-world practice. Evidence-based interventions like G-ANC may fail to achieve their intended effect if they are not implemented as designed or if contextual barriers undermine delivery [9,15,19]. Understanding why implementation succeeds in some settings and fails in others is therefore essential. However, there remains limited research in Tanzania that systematically examines the implementation factors shaping G-ANC implementation. Moreover, there is limited studies in similar settings have applied formal implementation science frameworks to guide the synthesis of G-ANC implementation factors.

The Consolidated Framework for Implementation Research (CFIR) offers a comprehensive approach for examining the complex, multilevel factors that influence implementation outcomes [27]. This framework integrates constructs across five domains, including intervention characteristics, outer setting, inner setting, characteristics of individuals, and implementation processes [27]. It provides a structured lens for identifying barriers and facilitators affecting program delivery. Applying the CFIR allows for a nuanced understanding of how G-ANC interacts with health system structures, provider practices, and community contexts in low-resource settings. The CFIR can be used to guide assessments of implementation contexts and inform implementation strategies aimed at improving the effectiveness of an intervention or program within a specific setting [28].

Guided by the CFIR framework, this study aimed to explore the factors influencing the implementation of G-ANC in low-resource settings in Tanzania. Applying the CFIR enabled comprehensive examination of multilevel implementation factors across diverse facility settings and generated context-specific insights to inform integration of G-ANC into routine ANC services in Tanzania and other similar settings.

## Methods

### Ethical statement

Ethical approval for the study was obtained from the National Institute of Medical Research in Tanzania (NIMR) through the National Health Research Ethics Committee (NatHREC) (ID No: NIMR/HQ/R.8a/Vol.IX/4194; Approval Date: 23 January 2023). All participants provided written consent prior to participation. Confidentiality and anonymity were maintained throughout the analysis and reporting by using unique ID and removing personal identifiers from transcripts. Participants were allowed to quit the research at any time without any consequences. Participants received copies of the signed consent forms and all documents were securely stored by the study team.

### Study design

This study adopted a qualitative research design. A qualitative design was selected to allow in-depth exploration of the contextual, organizational, and social dynamics shaping implementation, which are not readily captured through quantitative methods [29]. The Consolidated Criteria for Reporting Qualitative Research (COREQ) checklist was used to enhance the rigour and transparency of the study (S1 Table).

### Study setting

The study was conducted in the Geita Region, a predominantly rural region in north-western Tanzania. This region is characterized by dispersed populations, limited transport infrastructure, and resource-constrained health facilities. Six public health facilities were purposively selected to capture variation in implementation context and service delivery capacity. The selected facilities include hospitals, health centres, and dispensaries, reflecting different levels of the health system and varying patient volumes, staffing levels, and infrastructure.

### Intervention description

Group Antenatal Care was implemented under the project named “Mlinde Mama”. The Mlinde Mama Project, named after Kiswahili words meaning “protect a mother,” implemented by the Prime Health Initiative Tanzania (PHIT) with funding support from the Gates Foundation. Published findings from the project indicate that G-ANC contributed to improved ANC coverage and increased uptake of selected ANC services, including malaria testing, blood pressure monitoring, and preventive medications [19]. Women participating in G-ANC also reported positive experiences of care, increased satisfaction, and enhanced emotional and peer support [19,30].

Each step of the implementation was informed by existing evidence adapted to the Tanzania context. Initially, pregnant women attending their first ANC visit were screened for the eligibility criteria and informed consent to join G-ANC cohorts was sought. Eligibility included women who had a GA below 20 weeks, were willing to participate in group sessions, and had no serious mental health conditions or severe medical illnesses such as chronic hypertension. The name of each woman who consented to join G-ANC was recorded in an available GA specific cohort of 8–15 women. The cohort tracker included the name, cohort number, cohort name, contact address, and phone number for meeting reminders (S2 Table).

**Fig 1:**
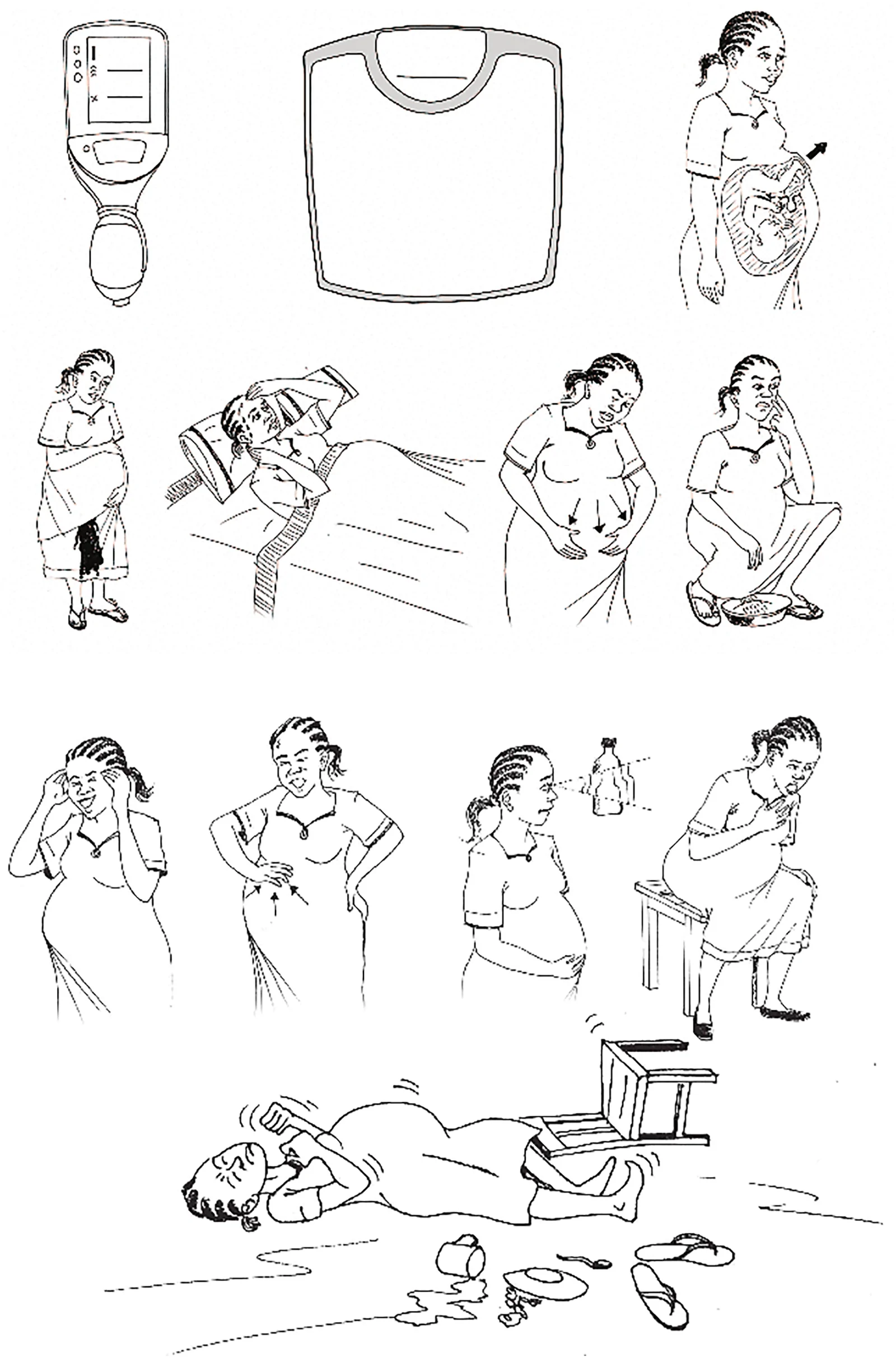
This figure illustrates a self-assessment card on which pregnant women record their blood pressure, body weight, and danger signs.

Group care sessions were composed of three main components including clinical assessment, participatory facilitated learning and peer support. At the start of each group visit (meeting), women took their own blood pressure (BP) and weight, reflected and recorded whether or not they had experienced any danger signs (Fig 1). Women participating in G-ANC received orientation and practical guidance from HCPs on how to measure BP and weight before group sessions. As women finished their self-assessments, they individually began seeing the ANC provider(s) for private consultations. The consultations with the provider included assessment of self-recorded BP and danger signs through review of the self-assessment card, determination of GA, measurement of fundal height, and auscultation of fetal heart tones.

**Fig 2:**
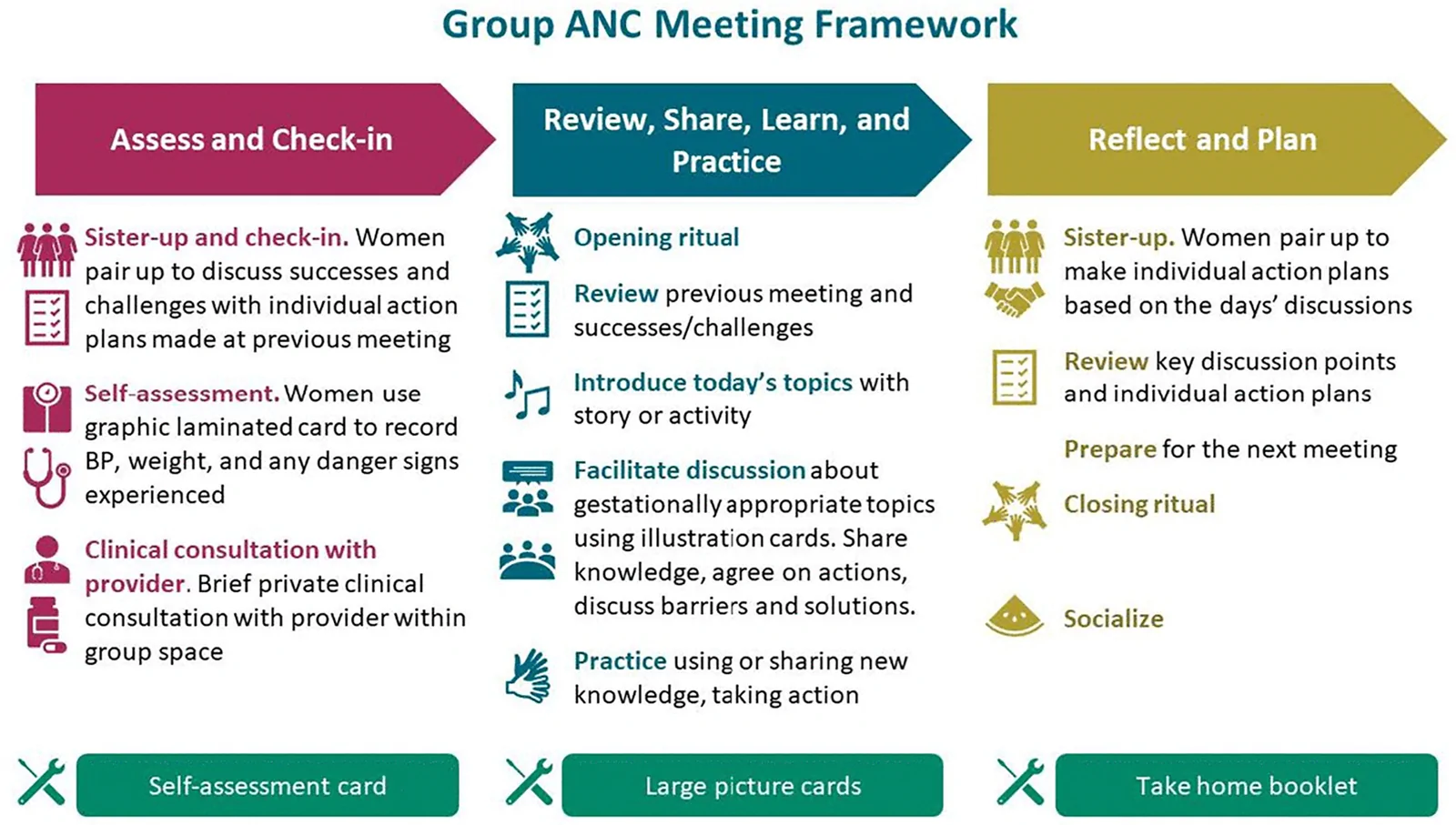
G-ANC meeting framework.

Participatory learning involved women sitting in a group circle. The main principle was the inclusion of activities that helped women reflect on their own resources, values, and ideas, and make plans for actions they wanted to take in relation to a healthy pregnancy and birth. These activities promoted empowerment and active discussion with reference to the provided maternal booklets (S1 File). At the end of each group meeting, HCPs/facilitators reflected on what went well and documented lessons learned. Reflections were guided by the quality-assurance tools and the facilitation fidelity checklist (S1 Text). Each meeting was guided as outlined in the G-ANC meeting framework (Fig 2).

### Data collection

A qualitative data collection was conducted between June and November 2024. Focus group discussions (FGDs) were conducted with women who had attended ANC through G-ANC. Key informant interviews (KIIs)/ In-depth interviews conducted with HCPs and health system managers involved in the organization and delivery of ANC services. Semi-structured interview guides were used to facilitate systematic exploration of implementation processes while allowing flexibility to probe emerging issues. The interview guides were used elsewhere, informed by existing similar G-ANC studies [18] and adapted to the Tanzanian health system context. The interview guides were piloted to assess clarity, relevance, flow of questions, and contextual appropriateness. The pilot process also helped refine wording, sequencing of probes, and interview approaches to improve consistency and depth of data collection across participant groups.

### Selection of participants

Participants were purposively selected from key stakeholder groups directly involved in the implementation and with experience of G-ANC. This included recently delivered women (≤6 months postpartum) or pregnant women attending G-ANC, HCPs facilitating the sessions, and health managers overseeing maternal health services. This approach allowed the study to capture real-life experiences and concrete examples of how G-ANC was delivered, perceived, and navigated within routine facility settings. Two FGDs were conducted with pregnant women, and four FGDs were conducted with postnatal women residing within the selected facilities’ catchment areas.

Snowball sampling approach was used to identify women who had previously participated in G-ANC but were no longer attending ANC clinics. In this process, the first few women were identified from the G-ANC cohorts and contacted through their phone numbers. Since G-ANC was implemented through cohorts of women with similar gestational ages, most participants stayed together for extended periods and became familiar with one another. The selected women then recommended other eligible participants by sharing their contacts or, in some cases, informing them directly in person. This process was further facilitated by HCPs or community health workers (CHWs) who were involved in the G-ANC cohorts. Each FGD involved between 8 and 10 participants.

Key informant interviews were conducted with HCPs directly involved in ANC delivery, including doctors, clinical officers and nurses or midwives. Facility in-charges were interviewed to capture leadership and organizational perspectives. In addition, district and regional-level maternal and reproductive health coordinators were interviewed to provide system-level insights.

### Sampling strategy and data saturation

Sample size was guided by the principles of purposive sampling, information power, and thematic saturation rather than numerical targets [31]. Participants were selected to ensure diversity across roles, facility levels (hospital, health centre, dispensary), and geographical contexts (urban, peri-urban, and rural). Data collection continued until thematic saturation was achieved, defined as the point at which additional FGDs or KIIs no longer yielded new or substantively different insights relevant to the facilitators and barriers to implementation [32]. Saturation was assessed iteratively within participant groups and across stakeholder categories to ensure adequate representation of perspectives from women, healthcare providers, and health managers. Triangulation was achieved by comparing perspectives across women, HCPs, and health managers, as well as across different facility types. This approach strengthened analytic credibility by enabling cross-validation of findings across implementation contexts [31].

### Data collection procedures

Interviews were conducted using semi-structured guides designed to elicit detailed accounts of implementation processes, enabling factors, and challenges encountered during G-ANC delivery. Core domains explored included:

- Operational experiences of implementing or participating in G-ANC
- Integration of G-ANC within conventional ANC workflows
- Facility, HCPs, and system-level facilitators supporting the implementation of G-ANC.
- Barriers affecting continuity and quality of G-ANC implementation.

Example prompts included: *“How is group ANC organized and delivered in this facility?”*, *“What has influenced the G-ANC model to work well in this setting?”*, and *“What challenges have affected the delivery or participation in group care?”* Probing questions were used to deepen discussion and clarify emerging themes.

All FGDs and KIIs were conducted in Kiswahili by trained qualitative researchers who were independent of service delivery to minimize response bias. Sessions were held in private, quiet locations within or near health facilities to ensure confidentiality and participant comfort. Interviews were audio-recorded with participant consent, and detailed field notes were taken to capture contextual factors and non-verbal interactions. Audio recordings were conducted using password-protected digital recording devices and secure mobile applications approved for research data collection. The software used did not require any authorized or licensed access. Each FGD was facilitated by a moderator and supported by a note-taker who documented group dynamics and key discussion points. In some sessions, an additional research team member assisted with coordination and participant flow.

### Data analysis

Audio recordings from all FGDs and KIIs were transcribed verbatim and translated into English by trained bilingual researchers (SC, JM and FP). Using NVivo Version 14.0 software, we managed and analysed all interviews to explore facilitators and barriers to the implementation of G-ANC. Initial thematic analysis was conducted using an inductive approach to categorize the data. As themes emerged, a codebook was developed to define the identified themes and was continuously refined through an iterative process to capture new aspects of the dataset.

Data were then grouped according to their relevance to the CFIR constructs (Fig 2). Discussions among researchers (AK, FP, JM and SC) were conducted to review the findings, and areas of disagreement were resolved by revisiting the data. This approach allowed for a broader understanding of topics that may have been misinterpreted by an individual researcher and helped identify issues that may have otherwise been overlooked.

**Fig 3:**
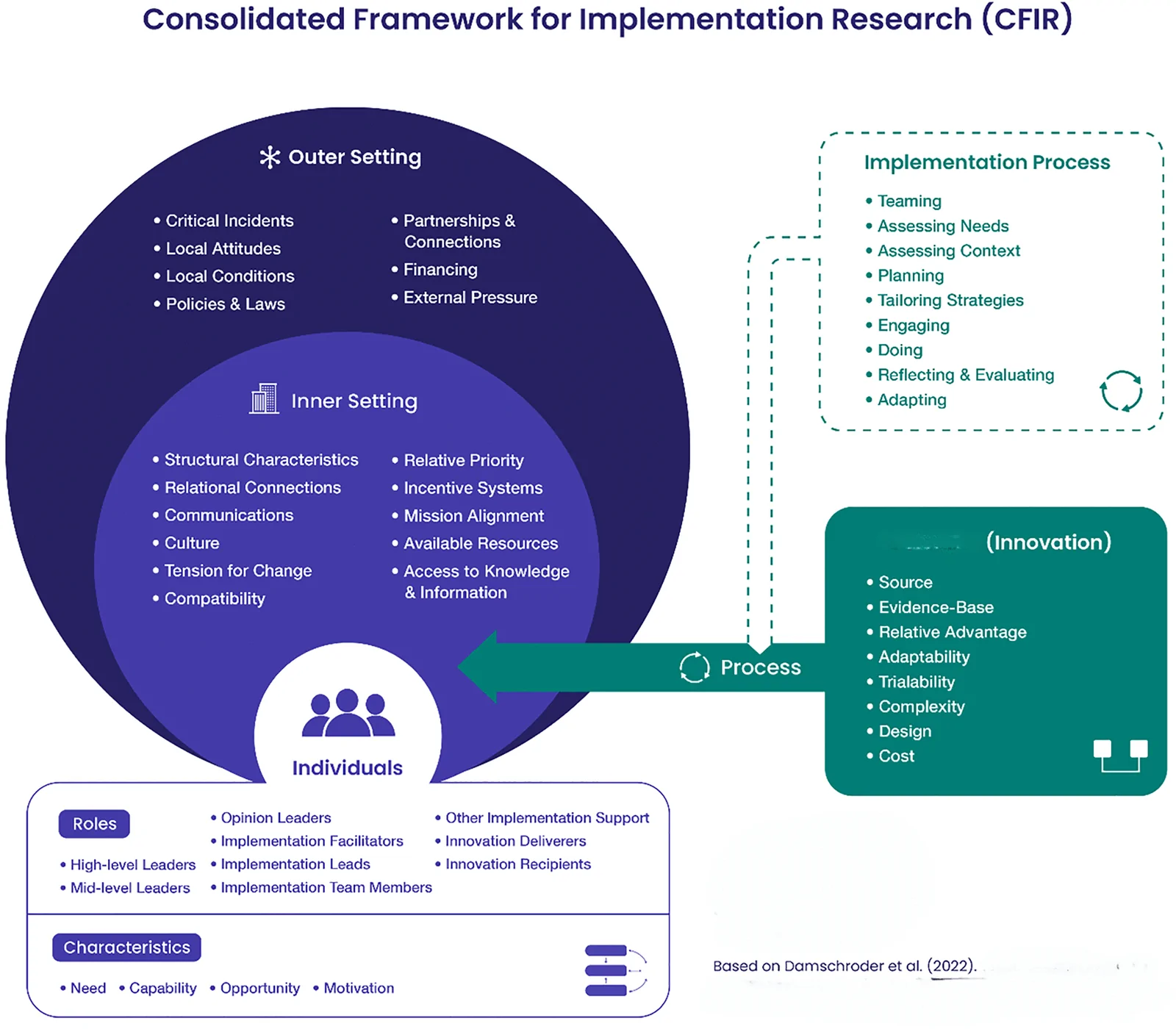
Consolidated Framework for Implementation Research (CFIR).

Synthesis of the data highlighted the implementation factors of G-ANC across all CFIR constructs including: (1) intervention characteristics, (2) outer setting, (3) inner setting, (4) characteristics of individuals, (5) implementation process. Use of this framework supports systematic comparison across settings and stakeholder groups and strengthen the transferability of findings to similar low-resource contexts (Fig 3) [27].

## Results

A total of 21 key informant interviews (KIIs) were carried out with antenatal care (ANC) providers, facility in-charges across all six facilities, and three health managers. Additionally, six focus group discussions were held, comprising four groups with 35 postnatal women and two groups with 18 pregnant women currently participating in group ANC sessions (Table 1).

**Table 1:** Characteristics of study participants.

Participant category	Characteristics	Respondents (n)
**Women (FGDs)**	Pregnant women	18
	Recently delivered women (≤6 months)	35
	**Facility/catchment area**	
	Nkome Dispensary	12
	Butengorumasa Dispensary	9
	Chato District Hospital	13
	Nzera District Hospital	12
	Bwanga Health Center	15
	Katoro Health Center	13
**Healthcare providers (KIIs)**	Medical doctors	3
	Clinical officers / assistant clinical officers	4
	Nurses/midwives	5
	Facility in-charges	6
**Health managers (KIIs)**	District health coordinators	2
	Regional Health Coordinator	1

### Implementation factors of G-ANC as organized into five domains of CFIR

#### Domain 1: Intervention Characteristics.

A dominant facilitator identified in this domain was the perceived relative advantage of G-ANC. Women and HCPs described G-ANC as a great approach for improving health education, peer learning, and women’s engagement during pregnancy. Providers valued the opportunity to deliver health education collectively rather than repeatedly to individual clients. One HCP explained:

*“Instead of repeating the same education many times, we discuss it once in the group and everyone benefits.”*

Another provider emphasized the participatory nature of the model, stating that:

*“In group care, women learn from each other, not only from the nurse... That does not happen in normal ANC.”*

Health managers also viewed the intervention positively because it aligned with broader maternal health priorities. As one manager noted:

*“Because this program was introduced through official channels and linked to maternal health goals, we felt it was something important to implement.”*

At the same time, the most consistently reported barriers were related to the complexity and resource demands of implementing G-ANC within already constrained health systems. Providers described challenges coordinating group sessions alongside routine clinical services, particularly in facilities with workforce shortages and limited infrastructure.

One provider explained:

*“Group care needs planning. You must organize women, prepare materials, and still attend other patients. It is not simple.”*

Resource constraints were also noted due to the absence of dedicated funding for infrastructure and staffing. As one health manager described:

*“We were told to implement group care using what we already have. There was no special budget for space or extra staff.”*

These findings indicate that although G-ANC was perceived as beneficial, successful implementation depended heavily on the ability of facilities to adapt the model within existing organizational and resource limitations.

Table 2 summarizes facilitators and barriers related to CFIR Domain 1 (Intervention Characteristics). It highlights how features of the G-ANC model itself shaped implementation across study sites.

**Table 2:** (Domain 1): Intervention characteristics.

CFIR Construct	Findings (Enablers and Barriers)	Illustrative quotes
**Intervention source**	**Enablers** 1. Healthcare providers and managers reported that G-ANC was introduced through a structured research initiative aligned with national maternal health priorities, which increased legitimacy and motivation for implementation at facility level. 2. Alignment with Ministry of Health priorities for improving ANC coverage and quality encouraged facilities to adopt the model despite limited resources.	*“Because this program was introduced through official channels and linked to maternal health goals, we felt it was something important to implement, not just as a stakeholders’ research.” Health manager*
**Evidence strength and quality**	**Enablers** 1. Providers and managers perceived G-ANC as improving women’s understanding of pregnancy-related information, attendance at ANC visits, and preparation for delivery. 2. Observed improvements in participation and continuity reinforced confidence in the intervention’s value.	*“When women attend group care, they understand better and come more consistently. You can see changes compared to the usual ANC.” Healthcare provider* *“You can see the difference in how women explain danger signs and prepare for delivery after attending group care.”Healthcare provider*
**Relative advantage**	**Enablers** 1. G-ANC was perceived as superior to conventional one-on-one ANC for delivering health education, fostering peer support, and strengthening provider–client interaction. 2. Providers reported reduced repetition of counselling messages compared to individual ANC visits.	*“Instead of repeating the same education many times, we discuss it once in the group and everyone benefits.” Healthcare provider* *“In group care, women learn from each other, not only from the nurse. That does not happen in normal ANC.” Healthcare provider*
**Adaptability**	**Enablers** 1. Facilities adapted group size, timing, and session location to accommodate space limitations, staffing constraints, and women’s availability. 2. Community-based sessions were used in some settings to reduce transport barriers and improve attendance.	*“We adjusted the meeting time and sometimes held sessions closer to the community so women could attend without difficulty.” Healthcare provider*
**Complexity**	**Barriers** 1. Providers described G-ANC as logistically demanding, requiring coordination of group sessions, individual clinical assessments, and cohort scheduling. 2. Managing group dynamics while attending to other clinical duties increased perceived complexity, especially in understaffed facilities.	*“Group care needs planning. You must organize women, prepare materials, and still attend other patients. It is not simple.” Healthcare provider*
**Design quality and packaging**	**Barriers** 1. Some facilities lacked adequate educational materials and visual aids to support interactive group discussions. 2. Inconsistent availability of tools for self-assessment (e.g., blood pressure equipment) affected fidelity to the model.	*“Sometimes the materials are not enough, so teaching becomes only talking instead of showing and practicing.” Facility in-charge*
**Cost**	**Barriers** Managers reported that G-ANC implementation relied largely on existing facility resources, with limited dedicated funding for space modification, materials, or additional staffing. **Enablers** Use of existing staff and infrastructure allowed implementation to proceed without major additional financial inputs.	*“We were told to implement group care using what we already have. There was no special budget for space or extra staff.” Health manager*

#### Domain 2: outer setting.

Within the outer setting domain, respondents appreciated the strong peer support and social connectedness created through G-ANC participation. Women described the group setting as encouraging shared learning, emotional support, and providing opportunity for continuity of care in ANC services. One pregnant woman explained:

*“When one woman misses a meeting, we ask her why she didn’t come. That encouragement helps us continue together.”*

Similarly, a postnatal woman stated.

*“We are used to learning together as women in the community. Sitting together and sharing feels normal.”*

Community-based delivery approaches also improved accessibility in some settings. As one HCP noted:

*“When the sessions are closer to the community, women attend more and are not in a hurry to leave.”*

These findings suggest that the alignment of G-ANC with existing community norms and peer networks strengthened women’s engagement and acceptability of the intervention.

However, the main barriers were socioeconomic constraints and concerns about privacy within group settings. Women from remote and low-income households often struggled to attend sessions consistently because of transport costs and competing domestic demands. One postnatal woman explained:

*“Sometimes I want to attend, but I don’t have money for transport. If I must choose between coming to the group and feeding my children, I stay at home.”*

Limited male partner support further constrained participation, with one pregnant woman reporting that

*“My husband says one clinic visit is enough. When he doesn’t understand the purpose of the group, it becomes difficult for me to attend all sessions.”*

In addition, concerns about confidentiality reduced willingness to discuss sensitive issues openly during sessions. As one woman explained:

*“There are things you want to ask, but you keep quiet because you don’t know how others will talk about it outside the group.”*

Together, these findings show that while peer support enhanced participation and continuity of care, broader socioeconomic and social realities continued to influence women’s ability to fully engage in G-ANC.

Table 3 presents facilitators and barriers related to CFIR Domain 2 (Outer Setting). It illustrates how community, household, and broader contextual factors influenced the implementation of G-ANC.

**Table 3:** (Domain 2): Outer setting.

CFIR Construct	Findings (Enablers and Barriers)	Illustrative quotes
**Patient needs and resources**	**Barriers:** 1. Women’s participation in G-ANC was constrained by transport costs, food insecurity, and competing household and income-generating responsibilities. 2. These constraints disproportionately affected women from remote areas and low-income households, limiting consistent attendance despite perceived benefits of G-ANC.	*“Sometimes I want to attend, but I don’t have money for transport. If I must choose between coming to the group and feeding my children, I stay at home.” Postnatal woman*
**Cosmopolitanism (community linkages)**	**Enablers** 1. Community-based delivery of some G-ANC sessions strengthened linkages between health facilities and communities, improving accessibility and attendance. 2. Involvement of community health workers supported follow-up and information sharing outside facility settings.	*“When the sessions are closer to the community, women attend more and are not in a hurry to leave. It becomes easier for us to follow them up.” Healthcare provider*
**External social norms and cultural context**	**Enablers** G-ANC aligned with existing communal learning traditions and norms of mutual support among women, facilitating engagement and participation. **Barriers:** In small communities, close social ties heightened concerns about privacy and confidentiality during group discussions.	*“We are used to learning together as women in the community. Sitting together and sharing feels normal.” Postnatal woman* *“Sometimes you don’t speak freely because the women in the group are also your neighbours.” Pregnant woman*
**Family and household influence**	**Barriers** 1. Limited male partner support affected women’s ability to attend G-ANC sessions consistently. 2. Some partners questioned the need for repeated group visits, particularly when benefits were not immediately visible.	*“My husband says one clinic visit is enough. When he doesn’t understand the purpose of the group, it becomes difficult for me to attend all sessions.” Pregnant woman*
**Peer influence and social support**	**Enablers** 1. Peer encouragement and shared accountability among women supported continued attendance and engagement in G-ANC. 2. Women reminded and motivated each other to attend sessions and follow health advice.	*“When one woman misses a meeting...we ask her why she didn’t come. That encouragement helps us continue together.” Pregnant woman*
**External policies and programs**	**Enablers** Alignment of G-ANC with broader national maternal health priorities supported acceptance at district and facility levels. **Barriers** Lack of explicit policy guidance on integrating G-ANC into routine ANC limited consistency in implementation across facilities.	*“The program fits well with maternal health goals, but we don’t yet have clear guidance on how it should fully replace or fit into routine ANC.” District health manager*
**Stigma and social risk**	**Barriers** 1. Fear of gossip or unintended disclosure discouraged some women from discussing sensitive health or social issues in group settings. 2. Women sometimes deferred sensitive questions to private consultations.	*“There are things you want to ask, but you keep quiet because you don’t know how others will talk about it outside the group.” Pregnant woman*

#### Domain 3: inner setting.

Within this domain, supportive leadership, teamwork, and organizational adaptability during implementation of G-ANC were commonly cited by respondents. Facilities where leaders actively supported group care demonstrated better coordination of sessions, stronger staff commitment, and greater continuity of implementation. One HCP explained:

*“If the in-charge treats group care as important, staff prepare for it. If not, other services take priority...”*

Regular communication among providers as part of teamwork also strengthened coordination of group sessions. As one provider stated:

*“When we communicate well as a team, group care runs smoothly. If communication is poor, sessions are easily interrupted.”*

In several facilities, teams adapted implementation approaches over time through shared reflection. A facility in-charge described this process by explaining that

*“At first, it was difficult, but after discussing as a team, we adjusted how we organize the groups and it became easier.”*

However, some barriers within this domain were related to limited infrastructure and workforce shortages or competing clinical demands. Some facilities lacked adequate and comfortable space to conduct group sessions. One facility in-charge explained:

*“Our facility was not built for group care. The rooms are small, and when many women attend, it becomes crowded and uncomfortable.”*

Shortages of HCPs further disrupted continuity of group sessions, particularly during emergencies and periods of high patient volume. A healthcare provider noted that

*“One nurse is covering many services. When emergencies come, group care has to wait.”*

Another provider explained.

*“The goals of group care fit well with ANC, but the challenge is fitting it into our daily workload without changes to the schedule.”*

Overall, these findings show that although organizational commitment supported implementation, structural and workforce limitations remained major barriers affecting expected successful implementation in some facilities.

Table 4 summarizes facilitators and barriers related to CFIR Domain 3 (Inner Setting), focusing on organizational and facility-level factors that shaped the implementation of G-ANC.

**Table 4:** (Domain 3): Inner setting.

CFIR Construct	Findings (Enablers and Barriers)	Illustrative quotes
**Structural characteristics**	**Barriers** Some facilities lacked adequate physical space to conduct group sessions while ensuring privacy and comfort.	*“Our facility was not built for group care. The rooms are small, and when many women attend, it becomes crowded and uncomfortable.” Facility in-charge*
**Networks and communication**	**Enablers** Regular communication among facility staff enabled coordination of group sessions, clinical assessments, and follow-up activities. **Barriers** In facilities with weak internal communication, scheduling conflicts and inconsistent messaging disrupted implementation.	*“When we communicate well as a team, group care runs smoothly. If communication is poor, sessions are easily interrupted.” Healthcare provider*
**Implementation climate – compatibility**	**Enablers** Providers perceived G-ANC as compatible with existing ANC objectives, particularly health education and birth preparedness. **Barriers** In some facilities, G-ANC was layered onto routine ANC without adjustments to workflows, creating tension with existing service delivery demands.	*“The goals of group care fit well with ANC, but the challenge is fitting it into our daily workload without changes to the schedule.” Healthcare provider*
**Implementation climate – relative priority**	**Enablers** Facilities where leadership prioritized G-ANC ensured protected time and staff commitment. **Barriers** Where G-ANC was viewed as secondary to other services, sessions were frequently postponed or interrupted.	*“If the in-charge treats group care as important, staff prepare for it. If not, other services take priority.” Healthcare provider*
**Readiness for implementation – leadership engagement**	**Enablers** Active involvement of facility in-charges in planning and monitoring G-ANC supported continuity and staff motivation.	*“When the leader follows up and asks about the sessions, we feel supported and take the work seriously.” Healthcare provider*
**Readiness for implementation – available resources**	**Barriers** 1. Shortages of HCPs limited the ability to dedicate staff exclusively to group sessions. 2. Inconsistent availability of educational materials and self-assessment tools affected session quality.	*“One nurse is covering my services (duties). When emergencies come, group care has to wait.” Healthcare provider*
**Implementation climate – learning environment**	**Enablers** Facilities that encouraged reflection and problem-solving adapted scheduling and delivery strategies over time to improve implementation.	*“At first, it was difficult, but after discussing as a team, we adjusted how we organize the groups and it became easier.” Facility in-charge*

#### Domain 4: characteristics of individuals.

Positive perception and confidence of both HCPs and women participating in G-ANC were highlighted by respondents as the main facilitators in this construct. Providers viewed the model as valuable for improving women’s understanding of pregnancy-related information and strengthening engagement in care. One HCP explained:

*“Group care helps women understand better, but at the beginning we were not sure if it would work with our limited staff.”*

However, confidence in facilitating group discussions improved with experience and repeated exposure to the model. As another provider stated:

*“At first, leading a group discussion was difficult, but after several sessions I became more confident.”*

Similarly, women described becoming more comfortable participating in discussions over time. One pregnant woman explained:

*“In the beginning, I was quiet, but after listening to others, I also started asking questions.”*

These findings suggest that familiarity with G-ANC gradually strengthened confidence, participation, and ownership among both providers and women.

On the other hand, important barriers cited in this domain were related to individual-level skills and confidence in facilitating participatory group discussions. In addition, some women remained hesitant to discuss sensitive issues openly within group settings, particularly during early sessions.

*“We were trained to provide routine ANC services, but facilitating group discussions was different. At the beginning, it was difficult to guide conversations, encourage participation, and manage the group at the same time... Some providers unsure about how to involve everyone equally during the sessions.”*

Similarly, one pregnant woman described concerns about discussing personal matters openly within the group setting:

*“In the first sessions, many women were quiet because we did not know each other well. Some topics are very personal, and you fear other people may talk about your problems outside the group. With time we became more comfortable...but at the beginning it was not easy.*

Despite these challenges, providers remained motivated to continue implementation because of the perceived benefits of the model. As one HCP explained:

*“Even when it is difficult, we continue because we see the benefit for the women.”*

Together, these findings indicate that successful implementation of G-ANC depends on building confidence among HCPs and women and improving providers’ facilitation skills.

Table 5 presents facilitators and barriers related to CFIR Domain 4 (Characteristics of Individuals). It shows how individual attributes, beliefs, and capacities influenced the implementation of G-ANC.

**Table 5:** (Domain 4): Characteristics of individuals.

CFIR Construct	Findings (Enablers and Barriers)	Illustrative quotes
**Knowledge and beliefs about the intervention**	**Enablers** Most HCPs viewed G-ANC as a valuable approach for improving women’s understanding of pregnancy-related health information and engagement with care. **Barriers** Some providers initially questioned whether group care could be delivered effectively within existing workloads and infrastructure constraints.	*“Group care helps women understand better, but at the beginning we were not sure if it would work with our limited staff.” Healthcare provider*
**Self-efficacy (providers)**	**Enablers** Healthcare providers reported increased confidence in facilitating group discussions over time, particularly after repeated sessions and peer support from colleagues. **Barriers** Limited prior training in facilitation skills made early sessions challenging for some providers.	*“At first, leading a group discussion was difficult, but after several sessions I became more confident.” Healthcare provider*
**Individual identification with the organization**	**Enabler** Healthcare providers who perceived G-ANC as aligned with their professional role and facility goals demonstrated greater commitment to implementation.	*“We see this as part of improving our maternal services, so we feel responsible for making it work.” Facility in-charge*
**Women’s confidence and engagement**	**Enablers** Participation in group sessions increased women’s confidence to ask questions and share experiences over time. **Barriers** Some women initially felt shy or hesitant to speak in group settings, particularly when discussing sensitive topics.	*“In the beginning, I was quiet, but after listening to others, I also started asking questions.” Pregnant woman*
**Personal attributes and social position**	**Barriers** Differences in age, education level, and social status among women influenced participation and willingness to speak during sessions.	*“Some women talk easily because they are educated, but others remain quiet and just listen.” Healthcare provider*
**Motivation and commitment**	**Enablers** Healthcare providers’ intrinsic motivation to improve maternal outcomes supported continued delivery of G-ANC despite challenges.	*“Even when it is difficult, we continue because we see the benefit for the women.” Healthcare provider*

#### Domain 5: process.

The main facilitators within the implementation process were deliberate planning, continuous adaptation (flexibility), and the presence of motivated champions supporting G-ANC delivery. Facilities that organized group schedules in advance and assigned clear responsibilities among staff experienced fewer disruptions during implementation. One facility in-charge explained:

*“When we plan the groups in advance and assign roles, the sessions go well. Without planning, things become chaotic.”*

Engagement of committed providers also strengthened continuity of implementation. As one health manager stated:

*“Having someone who believes in the program and pushes it forward makes a big difference.”*

In several facilities, routine reflection and flexibility helped maintain participation despite contextual challenges. One HCP described this process by explaining that

*“When we saw women struggling to come, we changed the location and time [schedule]  ...and attendance improved.”*

However, the most consistently reported process-related barriers were interruptions during service delivery. Group sessions were frequently disrupted by emergencies and competing clinical duties, particularly in facilities with limited staffing. One HCP explained:

*“Sometimes we start well, but an emergency comes and the group session is interrupted.”*

In some settings, inadequate coordination and unclear responsibilities contributed to delays and inconsistency in implementation.

*“Sometimes staff were not sure who was responsible for preparing the group sessions or following up the women. When communication was unclear, sessions started late or were postponed because everyone expected someone else to organize the activities.”*

Similarly, a facility in-charge described how inconsistent coordination disrupted continuity of implementation:

*“There were times when group sessions did not run as planned because duties were not clearly assigned among providers. Without proper coordination between staff...it became difficult to maintain the schedule...”*

In general, implementation of G-ANC, like other services requires flexibility and local adaptation. Also, continuity of G-ANC services requires strong coordination with other existing health services to reduce interruptions.

Table 6 summarizes facilitators and barriers related to CFIR Domain 5 (Process). This describes how planning, engagement, execution, and ongoing adaptation influenced the implementation of G-ANC.

**Table 6:** (Domain 5: Process).

CFIR Construct	Findings (Enablers and Barriers)	Illustrative quotes
**Planning**	**Enablers** Facilities that developed clear schedules and cohort plans for G-ANC sessions reported smoother implementation and fewer disruptions. **Barriers** Inadequate planning and unclear roles led to missed or delayed sessions, particularly in high-volume facilities.	*“When we plan the groups in advance and assign roles, the sessions go well. Without planning, things become chaotic.” Facility in-charge*
**Engaging Opinion Leaders and Champions**	**Enablers** Identification of motivated providers and facility leaders as champions supported staff engagement and continuity of implementation.	*“Having someone who believes in the program and pushes it forward makes a big difference.” Health manager*
**Engaging key stakeholders**	**Enablers** Engagement of district and facility leadership facilitated alignment of G-ANC with routine ANC services. **Barriers** Limited involvement of male partners constrained broader support for women’s participation.	*“If partners were more involved, women would find it easier to attend regularly.” Healthcare provider*
**Executing**	**Enablers** Facilities that integrated group discussions with individual clinical assessments delivered G-ANC more consistently. **Barriers** Frequent interruptions due to emergencies and competing services disrupted session flow and reduced quality.	*“Sometimes we start well, but an emergency comes and the group session is interrupted.” Healthcare provider*
**Reflecting and evaluating**	**Enablers** Informal reflection among providers enabled continuous adaptation of session timing, group size, and facilitation approaches. **Barriers** Lack of structured feedback mechanisms limited systematic learning across facilities.	*“We discuss among ourselves what worked and what didn’t, but there is no formal system to share lessons.” Facility in-charge*
**Adaptation during Implementation**	**Enablers** Ongoing adaptation, including shifting session locations and adjusting group composition, helped maintain participation under changing conditions.	*“When we saw women struggling to come, we changed the location and time, and attendance improved.” Healthcare provider*

### How CFIR domains interactions shaped fidelity and continuity of G-ANC

Our analysis showed that fidelity and continuity of G-ANC delivery were not determined by single CFIR domains in isolation, but rather by dynamic interactions across domains. At the intervention level, the perceived relative advantage and acceptability of G-ANC among women and providers created strong motivation to sustain group sessions. However, this motivation was frequently constrained by inner setting factors, most notably limited physical space, high routine ANC workload, and staffing shortages, which disrupted session flow, reduced privacy, and led to interruptions that compromised fidelity to the intended G-ANC model. Outer setting influences, including transport costs, competing household responsibilities, and limited male partner support, further interacted with inner setting constraints to affect attendance regularity and cohort continuity, particularly in rural and remote catchment areas.

**Fig 4:**
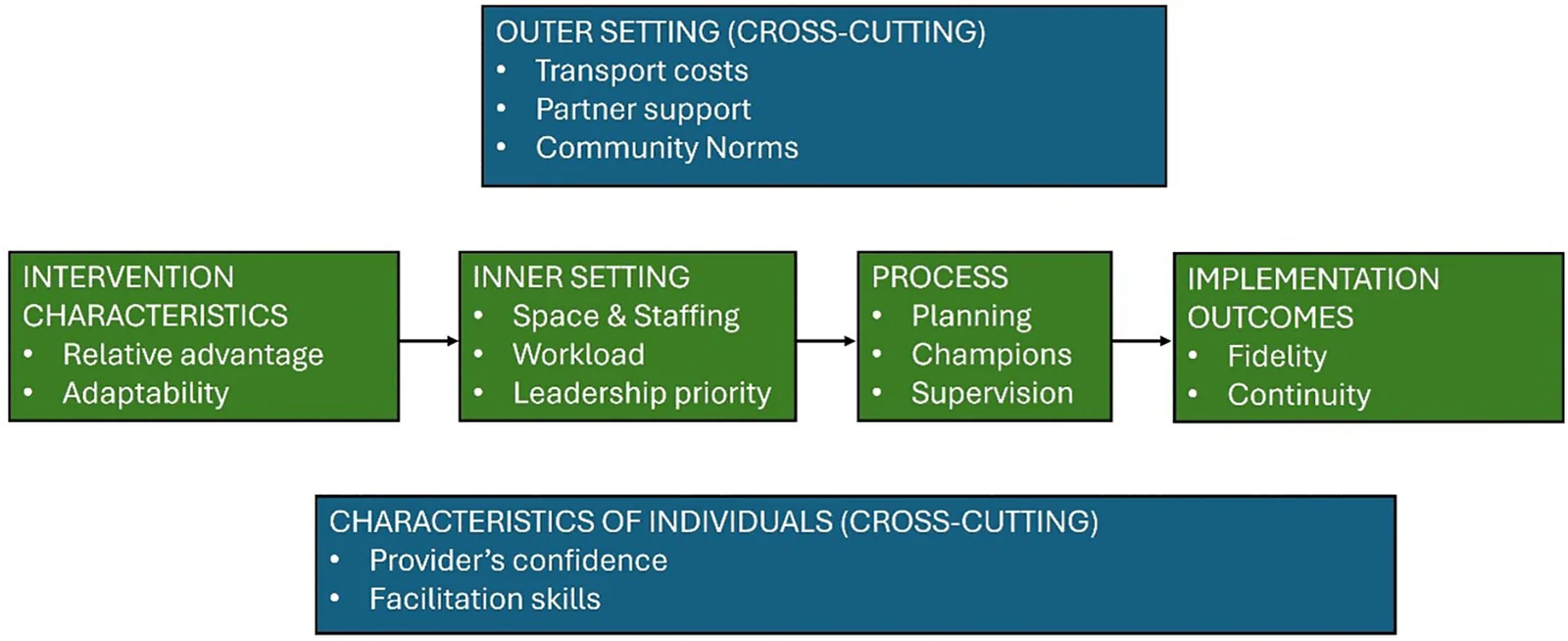
Interactions of CFIR domains in influencing fidelity and continuity of G-ANC delivery.

In facilities where leadership engagement was strong and G-ANC was prioritized within routine schedules, these constraints were partially mitigated through adaptive strategies such as reallocating space, adjusting session timing, or conducting sessions at the community level. The process domain played a critical moderating role: proactive planning, clear role assignment, and the presence of local champions enabled facilities to adapt G-ANC delivery while preserving core components, thereby maintaining continuity despite contextual constraints. In contrast, weak feedback mechanisms and limited monitoring reduced opportunities to identify and correct fidelity drift over time (Fig 4).

## Discussion

Although G-ANC has been implemented in several settings in Tanzania, its implementation has not consistently achieved the intended outcomes across all contexts. However, previous studies have often lacked strong theoretical guidance, and limited attention has been given to identifying facilitators and enabling factors influencing successful implementation of G-ANC. This study applied the CFIR to examine factors influencing the implementation of G-ANC in low-resource settings. Findings demonstrate that effective implementation of G-ANC are multifaceted and may occur at different levels, including the individual woman, HCPs, health facility, health system or policy levels, characteristics of the intervention, and implementation processes.

At the level of intervention characteristics, G-ANC was perceived to offer a clear relative advantage over conventional one-on-one ANC. Group care is characterized by strengthened health education delivery, facilitated peer learning, and improving efficiency in overstretched health systems. These are common outcomes of G-ANC, however, they are not unique since some systematic review studies have reported the same both in SSA [9] and in developed countries [15]. Similar advantages have been reported in studies from Kenya [25], Ghana [13], Rwanda [33], Bangladesh [14], and Nepal [23], where G-ANC enabled more comprehensive discussions, reduced repetition of counselling, and enhanced engagement of pregnant women in ANC services.

This study highlighted that these perceived advantages functioned as important motivators for HCPs and healthcare managers to support implementation, even in contexts with limited resources. However, as documented in other settings, the benefits of G-ANC were closely linked to its design and adaptability. Group care is designed to promote peer to peer support, individual clinical consultation, and self- assessment [9,15]. A pragmatic cluster-randomized controlled trial that was conducted in Nigeria and Kenya reported that these are reasons that G-ANC is most preferred compared to conventional ANC [25]. In this study, flexibility in group size, timing, and location, particularly the use of community-based sessions was critical for accommodating space constraints, staffing shortages, and women’s attendance. Evidence from Burkina Faso and Rwanda similarly highlights adaptability as a key determinant of acceptability in low-resource contexts [21,33].

Although HCPs described G-ANC as reducing repetitive counselling compared to conventional ANC, these efficiencies did not necessarily translate into reduced provider burden during G-ANC implementation. Instead, G-ANC often introduce additional coordination responsibilities, including cohort scheduling, facilitation of participatory discussions, and management of individual clinical consultations within the same session [9,12,21]. In facilities already constrained by workforce shortages and high patient volumes, G-ANC always implemented in parallel with conventional ANC services without corresponding adjustments to staffing, workflow organization [18,34]. At the same time, the HCPs described the logistical demands of coordinating more than one cohort on the same day and conducting individual clinical assessments alongside competing clinical responsibilities. These findings echo other studies indicating that G-ANC may increase perceived workload, especially during pilot studies or when introduced in parallel with routine ANC without adjustments to staffing norms or workflows [15]. As a result, the intended efficiency of G-ANC is partially offset by operational complexity and competing clinical demands. These findings suggest that efficiency within G-ANC should not be interpreted as automatic reductions in workload, but rather as a potential systems-level benefit that requires deliberate redistribution of tasks, strengthened teamwork, and organizational adaptation. Without such adjustments, G-ANC risks becoming an additional layer of work within already overstretched maternal health services.

Outer setting factors strongly influenced implementation by shaping women’s ability to participate consistently in G-ANC. Consistent with other studies in Tanzania [35–37], this study found that socioeconomic constraints, including transport costs and competing household responsibilities were major barriers, particularly for women in remote or low-income households. Similar barriers have been reported in other SSA countries [38–40], where poverty and geographic access limit utilization of maternal health services despite innovations in care delivery [41]. Gender dynamics further constrained participation, as limited male partner support affected women’s ability to attend follow-up ANC visits [42]. While a study in Malawi has documented increased male engagement through group-based care [24], findings from this study suggest that male involvement remains highly context-dependent and influenced by social norms.

Alignment of G-ANC with community values and social norms functioned as an important facilitator. Women perceived group care as consistent with traditions of communal learning and mutual support, which encouraged engagement and sustained participation. These findings concur evidence from other studies demonstrating that interventions aligned with existing social practices are more likely to be adopted and sustained [43,44]. However, close social proximity within small groups also heightened concerns about privacy and confidentiality, particularly when discussing sensitive issues. Similar tensions between social support and confidentiality have been reported in other G-ANC studies [45]. These notions are contrary to a large, a large cluster-randomized controlled trial conducted in Ghana which reported that women in the G-ANC reported greater respectful maternity outcomes than those in the control group (93.5% vs. 83%) [20]. This highlights the need for skilled facilitation and opportunities for additional time for private consultations. Skilled facilitators know how to balance sensitive group discussions while ensuring that more private concerns can be addressed during individual consultations. This aligns with the broader goals of the WHO respectful maternity care framework, which emphasizes privacy and confidentiality as essential components of respectful and compassionate care [46,47].

According to the CFIR framework, the inner setting includes features of the structural and cultural contexts through which the implementation process proceeds [28]. Inner setting factors including infrastructure, staffing, leadership, and facility settings were key to implementation success. Facilities with engaged leadership demonstrated stronger prioritization of G-ANC, protected staff time, and more consistent scheduling of sessions. This concurs with findings from other studies in SSA, which have repeatedly identified leadership engagement as a key enabler of health system innovations across diverse contexts [48,49]. In contrast, inadequate physical space, health workforce shortages, and competing clinical demands disrupted session flow and limited fidelity to the G-ANC model.

These constraints mirror findings from a systematic review conducted by Mihay et al. where insufficient space and staffing were reported to undermine G-ANC delivery despite positive perceptions of the intervention [15]. Importantly, facilities that fostered internal communication and learning were better able to adapt implementation strategies over time, suggesting that organizational learning capacity can mitigate structural constraints. This may be explained by the fact that good communication enables HCPs and facility teams to identify implementation challenges early and collectively develop context-specific solutions [50]. Second, continuous learning and information sharing promote adaptability and consistency in service delivery, allowing facilities to adjust implementation strategies in response to staffing or workflow constraints [51].

Characteristics of individuals reported to shape G-ANC implementation. This domain focuses on individuals engaged in the intervention and implementation process, who are carriers of cultural, organizational, professional mindsets, norms, interests, and affiliations [28]. Healthcare providers’ beliefs about the value of G-ANC and their confidence in facilitation skills influenced session quality and continuity. While HCPs initially reported uncertainty due to limited training in participatory methods, their confidence increased with experience. These findings are consistent with findings from similar studies in Kenya and Nigeria which emphasized the importance of capacity building for HCPs in facilitating G-ANC sessions [25,34]. Among women, participation in group sessions gradually increased confidence to ask questions and engage actively. Similarly, a systematic review found that most articles reported factors such as HCPs’ knowledge, beliefs and self-efficacy [15].These findings highlight that implementation success depends not only on structural readiness but also on investment in capacity-building and confidence among individuals involved.

Implementation processes including planning, engagement, execution, and reflection played a critical role in shaping implementation of G-ANC. This CFIR fifth domain focuses on requirement for active approaches to promote the adoption of the intervention and to achieve individual and organizational level use of the intervention as designed [28]. Facilities with well-structured planning in terms of allocating specific HCPs, space and integrated group sessions into routine workflows experienced fewer disruptions. In contrast, weak planning, frequent interruptions due to emergencies, and the lack of formal feedback mechanisms undermined consistency. Most of the previous G-ANC studies have documented the critical role of clear planning especially in recruitment and scheduling of cohorts [15]. Mehay et al.[15] emphasized that even well-designed G-ANC interventions require good structured implementation processes to sustain delivery effectiveness. Soyege et al. reported strategic planning in healthcare as crucial for adapting to change, enhancing patient outcomes, and ensuring long-term intervention success [52]. Effective planning and engagement may facilitate better coordination of resources, staff responsibilities, and streamlined service delivery processes [52].

### Implications of the study

The findings of this study provide important insights for strengthening maternal health policy in Tanzania and similar low-resource settings. The results support the growing recognition of G-ANC as a promising model for improving health education, peer support, and women’s engagement in pregnancy care. Moving beyond implementation research toward broader integration within national ANC guidelines may help promote more consistent and sustainable delivery of G-ANC across facilities. National and subnational policies can further support implementation by providing clear guidance on cohort organization, scheduling, staffing arrangements, documentation, and minimum standards for delivery. Such policy direction has the potential to strengthen coordination of services, improve continuity of care, and support integration of G-ANC within routine maternal health programs.

The study also highlights several practical opportunities for strengthening implementation within health facilities. Facilities that demonstrated teamwork and leadership engagement were better able to successfully implement G-ANC delivery despite resource limitations. Expanding investments in provider training, supportive supervision, task sharing, and protected time for group sessions may further strengthen implementation capacity and service quality. In addition, improving facility capacity through adaptable spaces for group discussions and private consultations can support respectful and client-centred care while maintaining privacy and confidentiality. Strengthening staffing capacity and organizational support systems may therefore enhance the continuity, acceptability, and effectiveness of G-ANC within routine ANC services.

Additionally, the findings provide important directions for future research. Further studies examining long-term integration and sustainability of G-ANC within routine health systems would strengthen understanding of how the model can be scaled effectively in low-resource settings. Future research should also explore the cost implications of G-ANC, its influence on provider workload over time, and strategies for improving equitable participation among women facing socioeconomic barriers. Expanding research to include policymakers, male partners, and community stakeholders are recommended to further generate valuable insights into how health systems and communities can collectively support successful implementation and scale-up of G-ANC.

### Strengths and limitations

This study has several notable strengths. First, the use of the CFIR provided a robust, theory-informed structure for examining multilevel factors influencing the implementation of G-ANC. Applying CFIR enabled systematic exploration of barriers and facilitators across intervention, organizational, individual, and contextual levels. This enhances the analytical rigor and interpretability of findings. Second, the inclusion of multiple stakeholder perspectives including women receiving care, HCPs, facility leaders, and health system managers strengthened the credibility of the findings through triangulation and allowed for comparison of experiences across roles and facility levels. Third, data were collected across diverse service delivery contexts, including hospitals, health centres, and dispensaries in rural and peri-urban settings, improving the relevance of findings for low-resource health systems.

Several limitations should also be considered when interpreting the findings of this study. The study was conducted in a single region of Tanzania, which may limit the transferability of findings to other settings with different health system structures, cultural norms, or levels of resource availability. Although efforts were made to include diverse participant groups, the perspectives of male partners, community leaders, and policymakers were not directly explored, which may have constrained understanding of broader social and policy influences on G-ANC implementation. In addition, as with all qualitative research, the findings are based on self-reported experiences and may be subject to social desirability or recall bias. The study also focused on implementation experiences during a research phase, and implementation dynamics may differ during long-term scale-up or institutionalization.

Women with more positive experiences or sustained participation in G-ANC may have been more likely to participate in the study because they were easier to identify through ongoing clinic attendance and more willing to share their experiences. Potentially, this may introduce selection and positive reporting bias. Although snowball sampling was used to identify postpartum women who were no longer actively attending ANC clinics, the perspectives from women who completely disengaged from care or declined participation may remain underrepresented. Despite these limitations, the study provides in-depth, contextually grounded insights that are valuable for informing adaptation and strengthening of G-ANC implementation in similar low-resource settings.

## Conclusion

The findings show that implementation of G-ANC is shaped by interacting factors across all five CFIR domains. G-ANC was perceived to offer important advantages including improved efficiency of health education, enhanced peer support, and stronger women engagement in pregnancy care. However, these benefits were constrained by limited infrastructure, health workforce shortages, and lack of dedicated resources.

Outer-setting factors, particularly socioeconomic constraints, gender dynamics, and privacy concerns, influenced women’s ability to participate consistently in group sessions. At the facility level, supportive leadership, effective teamwork, and organizational readiness facilitated implementation, while inadequate physical space and competing clinical demands disrupted continuity. Individual-level factors, including providers’ facilitation skills and women’s confidence to engage in group settings, further shaped the quality of implementation and evolved with experience. Implementation processes such as deliberate planning, presence of champions, and opportunities for adaptation supported sustained delivery, whereas weak coordination and frequent service interruptions undermined fidelity.

This study highlights that successful implementation of G-ANC requires alignment between intervention design, health system capacity, and community context. Addressing these multilevel facilitators and barriers is essential for integrating G-ANC into routine ANC and strengthening maternal and newborn health services in low-resource settings.

## Supporting information

S1 Table The Consolidated Criteria for Reporting Qualitative Research (COREQ) checklist. (DOCX)

S2 Table Cohort tracker. (PDF)

S1 Text Quality-assurance tools and the facilitation fidelity checklist. (PDF)

S1 File Maternal take home booklet. (PDF)

## References

[1] World Health Organization WHO. Maternal mortality. 2025. https://www.who.int/news-room/fact-sheets/detail/maternal-mortality Accessed 2026 January 21.

[2] United Nations International Children’s Emergency Fund (UNICEF). Neonatal mortality. 2025. https://data.unicef.org/topic/child-survival/neonatal-mortality/ Accessed 2025 December 28.

[3] World Health Organization WHO. African region’s maternal and newborn mortality declining, but progress still slow. 2025. https://www.afro.who.int/news/african-regions-maternal-and-newborn-mortality-declining-progress-still-slow Accessed 2026 February.

[4] Ministry of Health, National Bureau of Statistics. Demographic and Health Survey and Malaria Indicator Survey. 2022. https://dhsprogram.com/pubs/pdf/FR382/FR382.pdf

[5] World Health Organization, UNICEF, UFPA. Newborn mortality. https://www.who.int/news-room/fact-sheets/detail/newborn-mortality Accessed 2025 May 6.

[6] World Health Organization WHO. Antenatal care. 2018. https://platform.who.int/docs/default-source/mca-documents/policy-documents/policy-survey-reports/srmncah-policysurvey2018-fullreport-pt-3.pdf?sfvrsn=3465d5a7_4 Accessed 2026 January 21.

[7] World Health Organization WHO. WHO recommendations on antenatal care for a positive pregnancy experience. 2016. https://www.who.int/publications/i/item/9789241549912 Accessed 2026 January 21. 28079998

[8] Mgata S, Maluka SO. Factors for late initiation of antenatal care in Dar es Salaam, Tanzania: A qualitative study. BMC Pregnancy Childbirth. 2019;19(1):415. doi: 10.1186/s12884-019-2576-0 31718586 PMC6849280

[9] Lazar J, Boned-Rico L, Olander EK, McCourt C. A systematic review of providers’ experiences of facilitating group antenatal care. Reprod Health. 2021;18(1):180. doi: 10.1186/s12978-021-01200-0 34493314 PMC8425020

[10] Gutema EA, Ousman SK, Tamirat A, Tesemma M, Alemu ZA. Fund contraction challenges for supplies for maternal and newborn health services in low- and middle-income countries: a scoping review. BMC Public Health. 2025;25(1):4351. doi: 10.1186/s12889-025-25721-6 41316130 PMC12751249

[11] World Health Organization (WHO). Compendium on respectful maternal and newborn care [Internet]. 2025 [cited 2026 Feb 2]. Available from: https://iris.who.int/server/api/core/bitstreams/2ccf29b8-2c8d-45ba-a44c-a45138b88df4/content

[12] Sadiku F, Bucinca H, Talrich F, Molliqaj V, Selmani E, McCourt C, et al. Maternal satisfaction with group care: a systematic review. AJOG Glob Rep. 2023;4(1):100301. doi: 10.1016/j.xagr.2023.100301 38318267 PMC10839533

[13] Zielinski R, Kukula V, Apetorgbor V, Awini E, Moyer C, Badu-Gyan G, et al. “With group antenatal care, pregnant women know they are not alone”: The process evaluation of a group antenatal care intervention in Ghana. PLoS One. 2023;18(11):e0291855. doi: 10.1371/journal.pone.0291855 37934750 PMC10629640

[14] Sultana M, Ali N, Akram R, Jahir T, Mahumud RA, Sarker AR, et al. Group prenatal care experiences among pregnant women in a Bangladeshi community. PLoS One. 2019;14(6):e0218169. doi: 10.1371/journal.pone.0218169 31188891 PMC6561581

[15] Mehay A, Motta GD, Hunter L, Rayment J, Wiggins M, Haora P, et al. What are the mechanisms of effect of group antenatal care? A systematic realist review and synthesis of the literature. BMC Pregnancy Childbirth. 2024;24(1):625. doi: 10.1186/s12884-024-06792-6 39354405 PMC11446066

[16] Hunter LJ, Da Motta G, McCourt C, Wiseman O, Rayment JL, Haora P, et al. Better together: A qualitative exploration of women’s perceptions and experiences of group antenatal care. Women Birth. 2019;32(4):336–45. doi: 10.1016/j.wombi.2018.09.001 30253938

[17] Suleiman YM, Yohanna W, Aremu SO, Halidu GI, Adamu AI. Utilization, satisfaction, and perceived maternal health benefits of group antenatal care in Karu LGA, North Central, Nigeria. BMC Pregnancy Childbirth. 2025;26(1):73. doi: 10.1186/s12884-025-08579-9 41408528 PMC12829021

[18] Grenier L, Onguti B, Whiting-Collins LJ, Omanga E, Suhowatsky S, Winch PJ. Transforming women’s and providers’ experience of care for improved outcomes: A theory of change for group antenatal care in Kenya and Nigeria. PLoS One. 2022;17(5):e0265174. doi: 10.1371/journal.pone.0265174 35503773 PMC9064109

[19] Hellar A, Bandio R, Ernest E, Makuwani A, Kinyina A, Sospeter P, et al. Integrating group antenatal care into routine services: a registry-based cohort study in Geita, Tanzania. BMC Glob Public Health. 2026;4(1):12. doi: 10.1186/s44263-026-00243-4 41622235 PMC12862910

[20] Lanyo TN, Zielinski R, Kukula VA, Apetorgbor VEA, Ghosh B, Lockhart NA, et al. Improving respectful maternity care through group antenatal care: Findings from a cluster randomized controlled trial. Midwifery. 2025;147:104457. doi: 10.1016/j.midw.2025.104457 40378481 PMC12146043

[21] Dao B, Ouedraogo Y, Mhlanga M, Kone A. Exploring the acceptability and impact of group antenatal care: a qualitative study among women in selected health facilities in Burkina Faso. Reprod Health. 2025;22(1):265. doi: 10.1186/s12978-025-02085-z PMC12751604 41466411

[22] McKinnon B, Sall M, Vandermorris A, Traoré M, Lamesse-Diedhiou F, McLaughlin K, et al. Feasibility and preliminary effectiveness of group antenatal care in Senegalese health posts: a pilot implementation trial. Health Policy Plan. 2020;35(5):587–99. doi: 10.1093/heapol/czz178 32155254

[23] Thapa P, Bangura AH, Nirola I, Citrin D, Belbase B, Bogati B, et al. The power of peers: an effectiveness evaluation of a cluster-controlled trial of group antenatal care in rural Nepal. Reprod Health. 2019;16(1):150. doi: 10.1186/s12978-019-0820-8 31640770 PMC6805428

[24] Jeremiah RD, Patel DR, Chirwa E, Kapito E, Mei X, McCreary LL, et al. A randomized group antenatal care pilot showed increased partner communication and partner HIV testing during pregnancy in Malawi and Tanzania. BMC Pregnancy Childbirth. 2021;21(1):790. doi: 10.1186/s12884-021-04267-6 34819018 PMC8611988

[25] Grenier L, Suhowatsky S, Kabue MM, Noguchi LM, Mohan D, Karnad SR, et al. Impact of group antenatal care (G-ANC) versus individual antenatal care (ANC) on quality of care, ANC attendance and facility-based delivery: A pragmatic cluster-randomized controlled trial in Kenya and Nigeria. PLoS One. 2019;14(10):e0222177. doi: 10.1371/journal.pone.0222177 31577797 PMC6774470

[26] Patil CL, Klima CS, Steffen AD, Leshabari SC, Pauls H, Norr KF. Implementation challenges and outcomes of a randomized controlled pilot study of a group prenatal care model in Malawi and Tanzania. Int J Gynaecol Obstet. 2017;139(3):290–6. doi: 10.1002/ijgo.12324 28905377 PMC5673548

[27] Damschroder LJ, Reardon CM, Widerquist MAO, Lowery J. The updated Consolidated Framework for Implementation Research based on user feedback. Implement Sci. 2022;17(1):75. doi: 10.1186/s13012-022-01245-0 36309746 PMC9617234

[28] Kirk MA, Kelley C, Yankey N, Birken SA, Abadie B, Damschroder L. A systematic review of the use of the Consolidated Framework for Implementation Research. Implementation Science. 2015;11(1):72. doi: 10.1186/s13012-016-0437-z PMC4869309 27189233

[29] Busetto L, Wick W, Gumbinger C. How to use and assess qualitative research methods. Neurol Res Pract. 2020;2:14. doi: 10.1186/s42466-020-00059-z 33324920 PMC7650082

[30] Kinyina A, Hellar A, Bandio R, Mandali H, Makuwani A, Munga A, et al. Perspectives of women, healthcare providers and health managers on Group Antenatal Care implementation in Geita, Tanzania: A qualitative study. PLoS One. 2026;21(4):e0345539. doi: 10.1371/journal.pone.0345539 41984864 PMC13082642

[31] Hennink M, Kaiser BN. Sample sizes for saturation in qualitative research: A systematic review of empirical tests. Soc Sci Med. 2022;292:114523. doi: 10.1016/j.socscimed.2021.114523 34785096

[32] Saunders B, Sim J, Kingstone T, Baker S, Waterfield J, Bartlam B, et al. Saturation in qualitative research: exploring its conceptualization and operationalization. Qual Quant. 2018;52(4):1893–907. doi: 10.1007/s11135-017-0574-8 29937585 PMC5993836

[33] Musabyimana A, Lundeen T, Butrick E, Sayinzoga F, Rwabufigiri BN, Walker D, et al. Before and after implementation of group antenatal care in Rwanda: a qualitative study of women’s experiences. Reprod Health. 2019;16(1):90. doi: 10.1186/s12978-019-0750-5 31248425 PMC6595554

[34] Noguchi L, Grenier L, Kabue M, Ugwa E, Oyetunji J, Suhowatsky S, et al. Effect of group versus individual antenatal care on uptake of intermittent prophylactic treatment of malaria in pregnancy and related malaria outcomes in Nigeria and Kenya: analysis of data from a pragmatic cluster randomized trial. Malar J. 2020;19(1):51. doi: 10.1186/s12936-020-3099-x 31996209 PMC6990503

[35] Chamani AT, Mori AT, Robberstad B. Implementing standard antenatal care interventions: health system cost at primary health facilities in Tanzania. Cost Eff Resour Alloc. 2021;19(1):79. doi: 10.1186/s12962-021-00325-0 34876154 PMC8650535

[36] Mwalupani N, Macha L, Zakayo E. Socio-economic Factors Associated with Desired Number of Antenatal Care Visits in Tanzania. RPJ. 2024;26(1):106–22. doi: 10.59557/rpj.26.1.2024.70

[37] Rwabilimbo AG, Ahmed KY, Page A, Ogbo FA. Trends and factors associated with the utilisation of antenatal care services during the Millennium Development Goals era in Tanzania. Trop Med Health. 2020;48(1):38. doi: 10.1186/s41182-020-00226-7 PMC7268642 32518496

[38] Ilori T, Adewale BA, Obembe TA, Morakinyo OM. Socio-economic factors associated with antenatal care in Nigeria. Afr J Reprod Health. 2022;26(8):123–33. doi: 10.29063/ajrh2022/v26i8.12 37585038

[39] Sui Y, Ahuru RR, Huang K, Anser MK, Osabohien R. Household Socioeconomic Status and Antenatal Care Utilization Among Women in the Reproductive-Age. Front Public Health. 2021;9:724337. doi: 10.3389/fpubh.2021.724337 34589464 PMC8475754

[40] Dickson KS, Kwabena Ameyaw E, Akpeke M, Mottey BE, Adde KS, Esia-Donkoh K. Socio-economic disadvantage and quality Antenatal Care (ANC) in Sierra Leone: Evidence from Demographic and Health Survey. PLoS One. 2023;18(1):e0280061. doi: 10.1371/journal.pone.0280061 36634154 PMC9836291

[41] Muchemwa G, Khozah MY. Accessibility and barriers to maternal health services for adolescents in Sub-Saharan Africa: a scoping review. Ther Adv Reprod Health. 2025;19. doi: 10.1177/26334941251403814 PMC12743789 41466601

[42] Tesha J, Fabian A, Mkuwa S, Misungwi G, Ngalesoni F. The role of gender inequities in women’s access to reproductive health services: a population-level study of Simiyu Region Tanzania. BMC Public Health. 2023;23(1):1111. doi: 10.1186/s12889-023-15839-w 37296416 PMC10251643

[43] Hassan S, Kagwanja N, Diallo B, Oyando R, Perel P, Etyang A, et al. Implementing community-based interventions for the management of chronic conditions in low- and middle-income countries: A scoping review of qualitative evidence. PLOS Glob Public Health. 2025;5(7):e0004860. doi: 10.1371/journal.pgph.0004860 40627592 PMC12237052

[44] Felisian S, Mushy SE, Tarimo EAM, Kibusi SM. Sociocultural practices and beliefs during pregnancy, childbirth, and postpartum among indigenous pastoralist women of reproductive age in Manyara, Tanzania: a descriptive qualitative study. BMC Womens Health. 2023;23(1):123. doi: 10.1186/s12905-023-02277-4 36959588 PMC10035110

[45] Gaur BPS, Vasudevan J, Pegu B. Group Antenatal Care: A Paradigm Shift to Explore for Positive Impacts in Resource-poor Settings. J Prev Med Public Health. 2021;54(1):81–4. doi: 10.3961/jpmph.20.349 33618503 PMC7939754

[46] Nassali MN, Russell JC, Tsuaneng M, Tumagole A, Mussa A, Moreri-Ntshabele B, et al. Promoting respectful maternity care with the WHO labor care guide and the checklist mnemonic “COPE”: A quality improvement project. Int J Gynaecol Obstet. 2025;171(2):798–804. doi: 10.1002/ijgo.70238 40411352 PMC12553107

[47] Kawish AB, Umer MF, Arshed M, Khan SA, Hafeez A, Waqar S. Respectful maternal care experience in low- and middle-income countries: a systematic review. Medicina (B Aires). 2023;59(10):1842. doi: 10.3390/medicina59101842 PMC10608623 37893560

[48] Nzinga J, Boga M, Kagwanja N, Waithaka D, Barasa E, Tsofa B, et al. An innovative leadership development initiative to support building everyday resilience in health systems. Health Policy Plan. 2021;36(7):1023–35. doi: 10.1093/heapol/czab056 34002796 PMC8359752

[49] Adebisi OT, Akinwaare MO. Exploration of facilitators and barriers to the implementation of the eight-contact antenatal care model in healthcare facilities in Nigeria. Matern Health Neonatol Perinatol. 2026;12(1):12. doi: 10.1186/s40748-026-00256-9 41928349 PMC13047834

[50] Gharaveis A, Hamilton DK, Pati D. The Impact of Environmental Design on Teamwork and Communication in Healthcare Facilities: A Systematic Literature Review. HERD. 2017;11(1):119–37. doi: 10.1177/1937586717730333 29022368

[51] Kraft S, Caplan W, Trowbridge E, Davis S, Berkson S, Kamnetz S, et al. Building the learning health system: Describing an organizational infrastructure to support continuous learning. Learn Health Syst. 2017;1(4):e10034. doi: 10.1002/lrh2.10034 31245569 PMC6508554

[52] Soyege OS, Nwokedi CN, Tomoh BO, Mustapha AY, Mbata AO, Balogun OD. Strategic planning in healthcare: a framework for sustainable growth and service excellence. International Journal of Multidisciplinary Research and Growth Evaluation. 2024;5(6):1579–83. doi: 10.54660/.IJMRGE.2024.5.6.1579-1583

