# Supplementary material for: Implementation factors of group antenatal care in low-resource settings in Tanzania: A qualitative study using the Consolidated Framework for Implementation Research
Source: PLOS Glob Public Health. 2026 Sep 18;6(9):e0006221. doi: 10.1371/journal.pgph.0006221 (PMC13588383; doi:10.1371/journal.pgph.0006221)
Supplement: Supplementary file 1 [file pgph.0006221.s002.pdf]

Group Antenatal Care

(G-ANC)

Cohort Enrollment and Attendance Trackers

DATES:

At enrollment: Check that her EDD matches the range for this cohort. Fill in name, phone, GA at ANC1, and EDD. **At every meeting**: for each woman, check (√) the "A" column if she attended. Record [1]actual delivery date if known. **If participation stops:** If you learn of a reason that a woman stops coming to G-ANC, mark the relevant meeting column in the register with the appropriate code. Codes include: M=moved away; SB=stillbirth (pregnancy loss); CB=childbirth; MD=maternal death; NLI=no longer interested. If the woman does not want to be (or cannot) be contacted before future meetings, draw a line from this code across through the last box.

Cohort #:			Cohort name:			EDD range (write dates):										
						First Meeting on										
SN	ANC NO	Name	Contact info (phone)	GA* at ANC1	EDD*	Called to remind (C), Attended Meeting (A) & Took IPTp (I)										Actual Delivery date
						M1: 2 March		M2:		M3:		M4:		M5:		
						C	A	C	A	C	A	C	A	C	A	
TOTAL ENROLLED=			TOTAL Attended by meeting (%)													

[1] Gestational age=GA; EDD=estimated delivery date; A=Attended this meeting

Cohort EDD Range List for

Cohort Number	Date of Meeting 1 (Day of the week)	EDD if 24 weeks GA at Meeting 1	EDD if 20 weeks GA at Meeting 1
C1.			
C2.			
C3.			
C4.			
C5.			
C6.			
C7.			
C8.			
C9.			
C10.			
C11.			
C12.			
C13.			
C14.			
C15.			
C16.			
C17.			
C18.			
