# Supplementary material for: Implementation factors of group antenatal care in low-resource settings in Tanzania: A qualitative study using the Consolidated Framework for Implementation Research
Source: PLOS Glob Public Health. 2026 Sep 18;6(9):e0006221. doi: 10.1371/journal.pgph.0006221 (PMC13588383; doi:10.1371/journal.pgph.0006221)
Supplement: Supplementary file 3 [file pgph.0006221.s004.pdf]

HUDUMA ZA AFYA YA UZAZI WAKATI WA UJAUZITO KWA NJIA YA KIKUNDI

Chukua hatua kwa afya ya mama na mtoto

Mkutano 1:	Mkutano 2:	Mkutano 3:	Mkutano 4:	Mkutano 5:
Wiki ya 20-24	Wiki ya 24-28	Wiki ya 28-32	Wiki ya 32-36	Wiki ya 36-40

1 Kuzuia matatizo wakati wa ujauzito

LALA KWENYE CHANDARUA CHENYE DAWA WAKATI WOTE WA UJAUZITO NA KUNYWA DAWA YA SP KAMA TIBA YA TAHADHARI YA MALARIA KWA VIPINDI

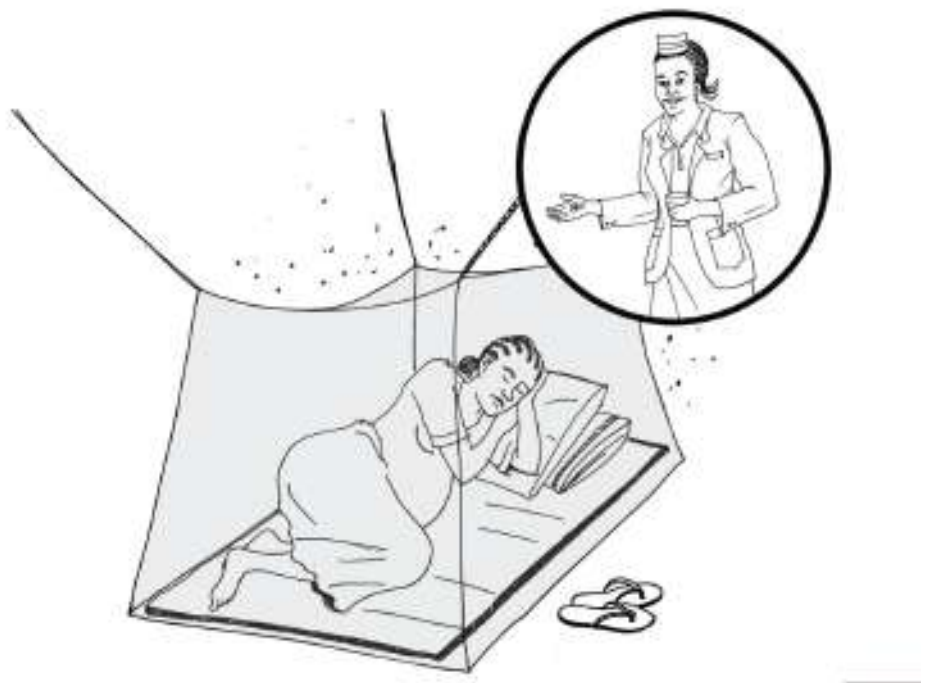

KULA VYAKULA MBALIMBALI NA KUNYWA VINYWAJI ZAIDI KILA SIKU. USIVUTE SIGARA AU KUNYWA POMBE

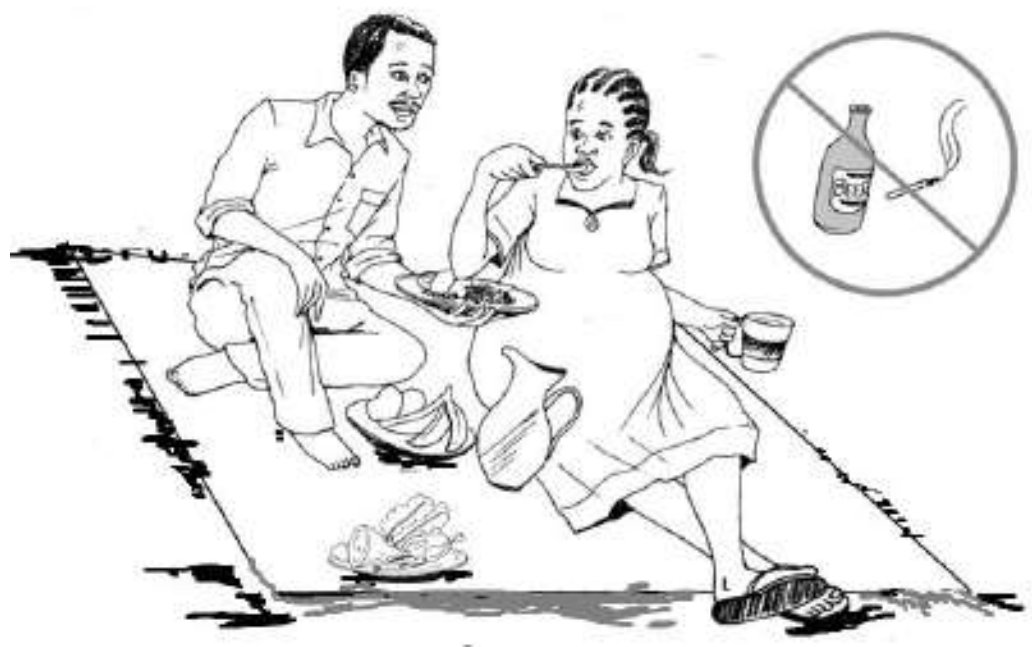

NAWA MIKONO BAADA YA KUTUMIA CHOO AU KUMBADILISHA MTOTO NEPI. NAWA MIKONO KABLA YA KUANDAA CHAKULA AU KABLA YA KULA

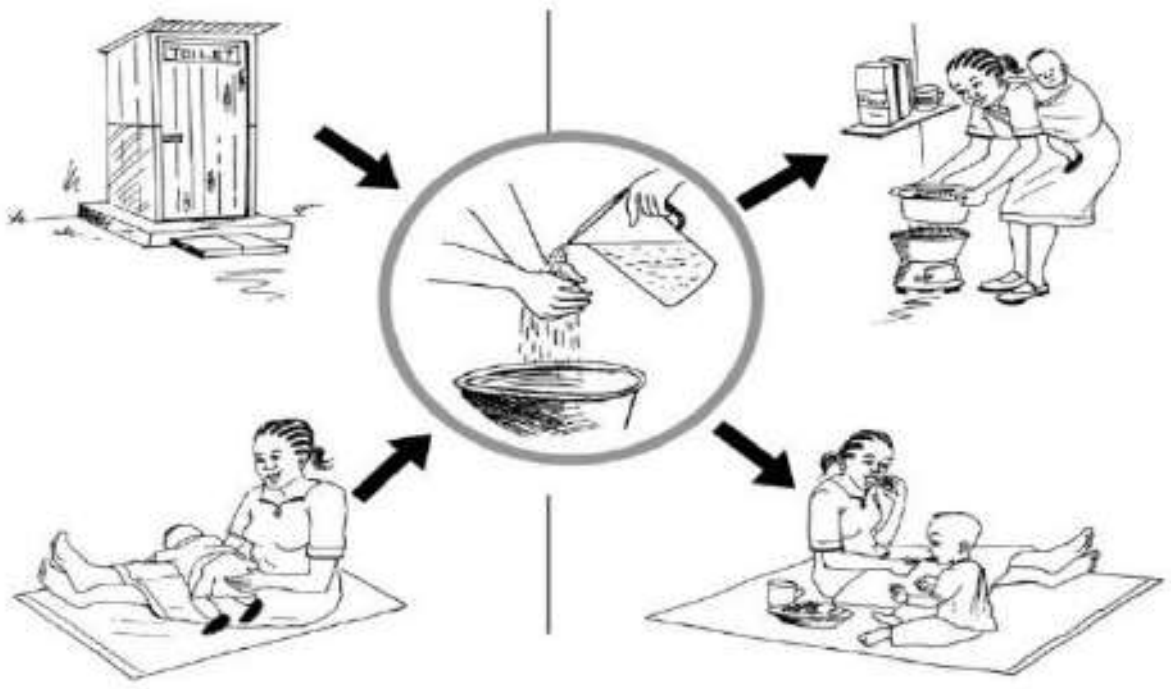

MEZA VIDONGE VYA MADINI CHUMA NA FOLIKI ACID KILA SIKU HADI MTOTO AKIWA NA UMRI WA MIEZI MITATU. PIA KULA VYAKULA VYENYE MADINI CHUMA NA FOLIK KWA WINGI.

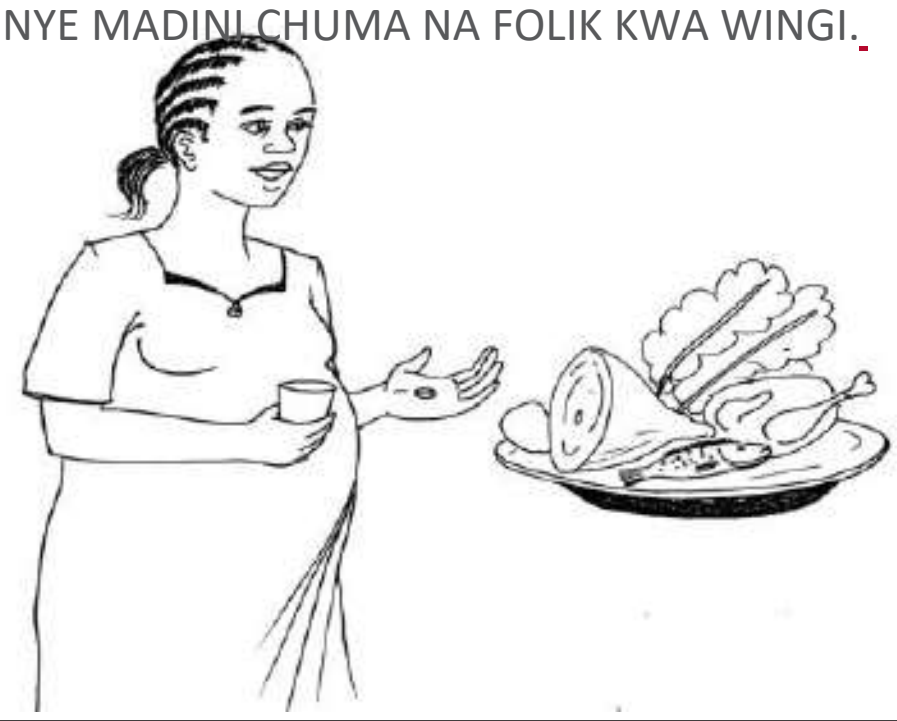

JIKINGE NA MAAMBUKIZI YA UKIMWI NA MAGONJWA YANAYOAMBUKIZWA KWA KUJAMIIANA

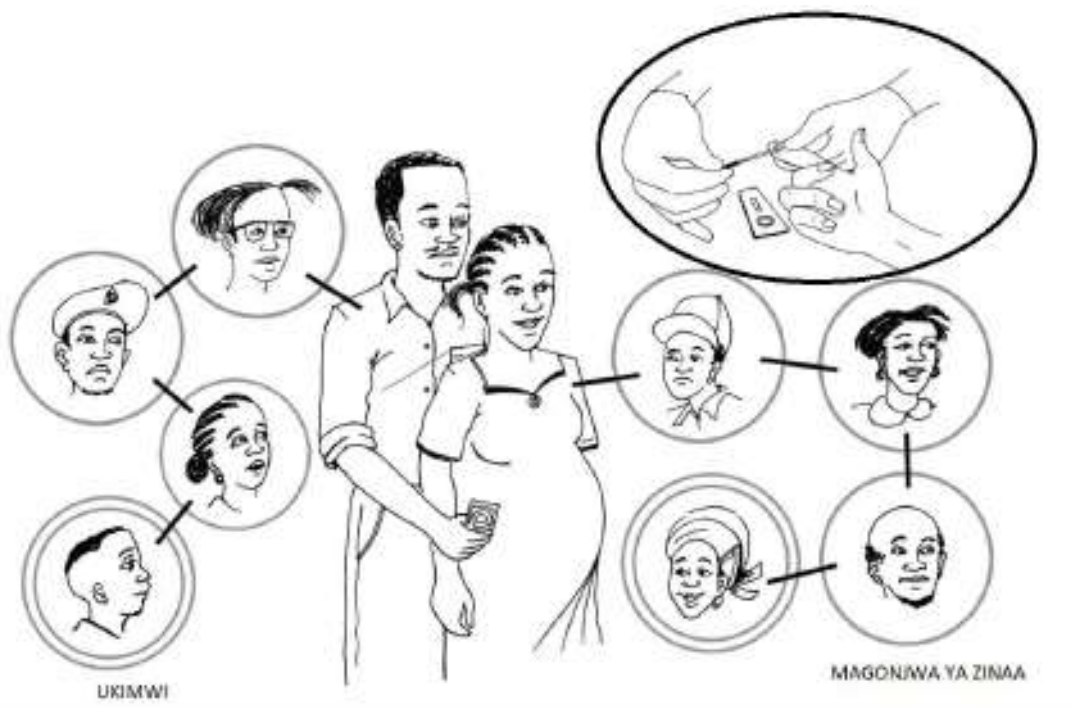

HUDHURIA KLINIKI YA WAJAWAZITO KUFUATANA NA RATIBA. RUDI WAKATI WOWOTE UONAPO DALILI YOYOTE YA HATARI

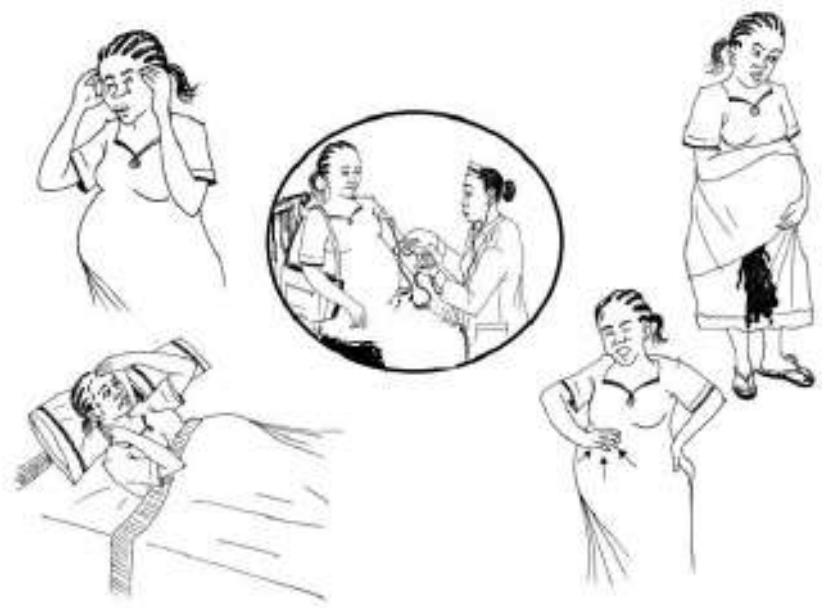

USIFANYE KAZI NGUMU. PUMZIKA ANGALAU KWA SAA 1 WAKATI WA MCHANA

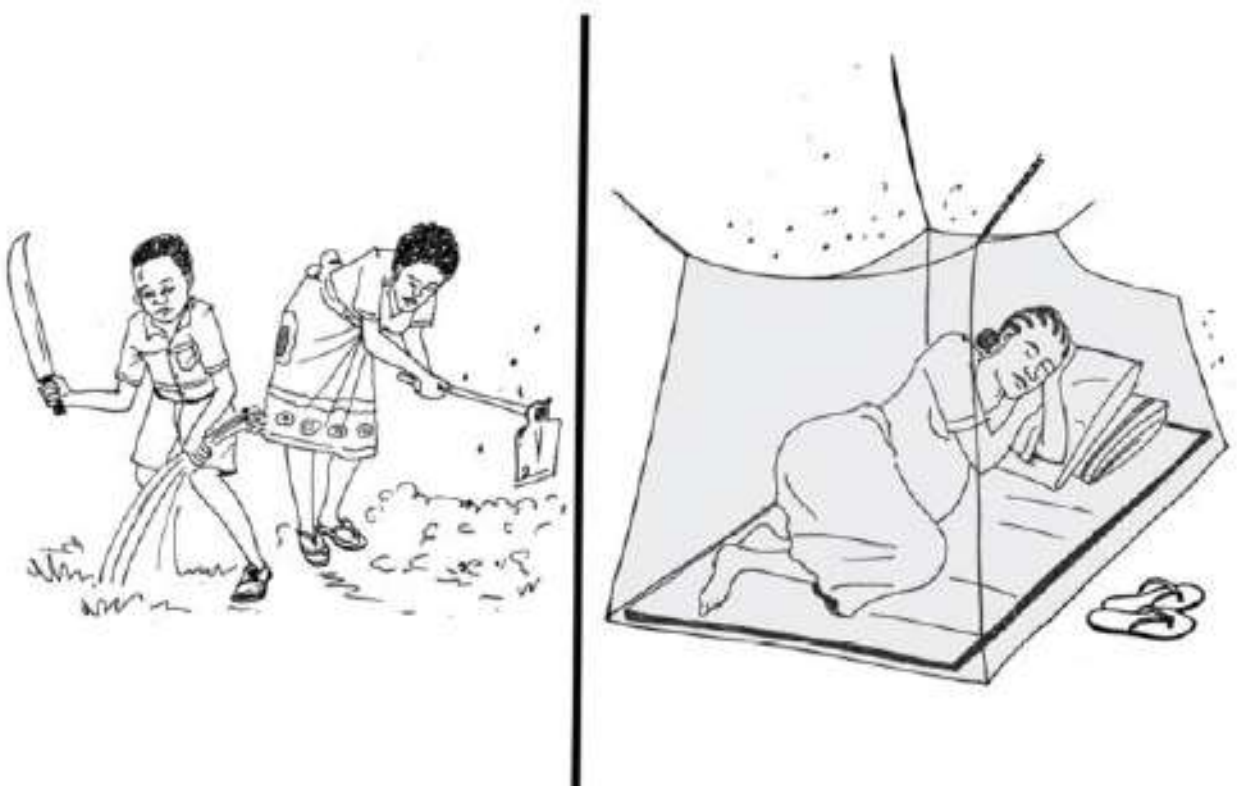

2 Dalili za hatari wakati wa ujauzito

DALILI ZA KIFAFA NA KIFAFA CHA MIMBA

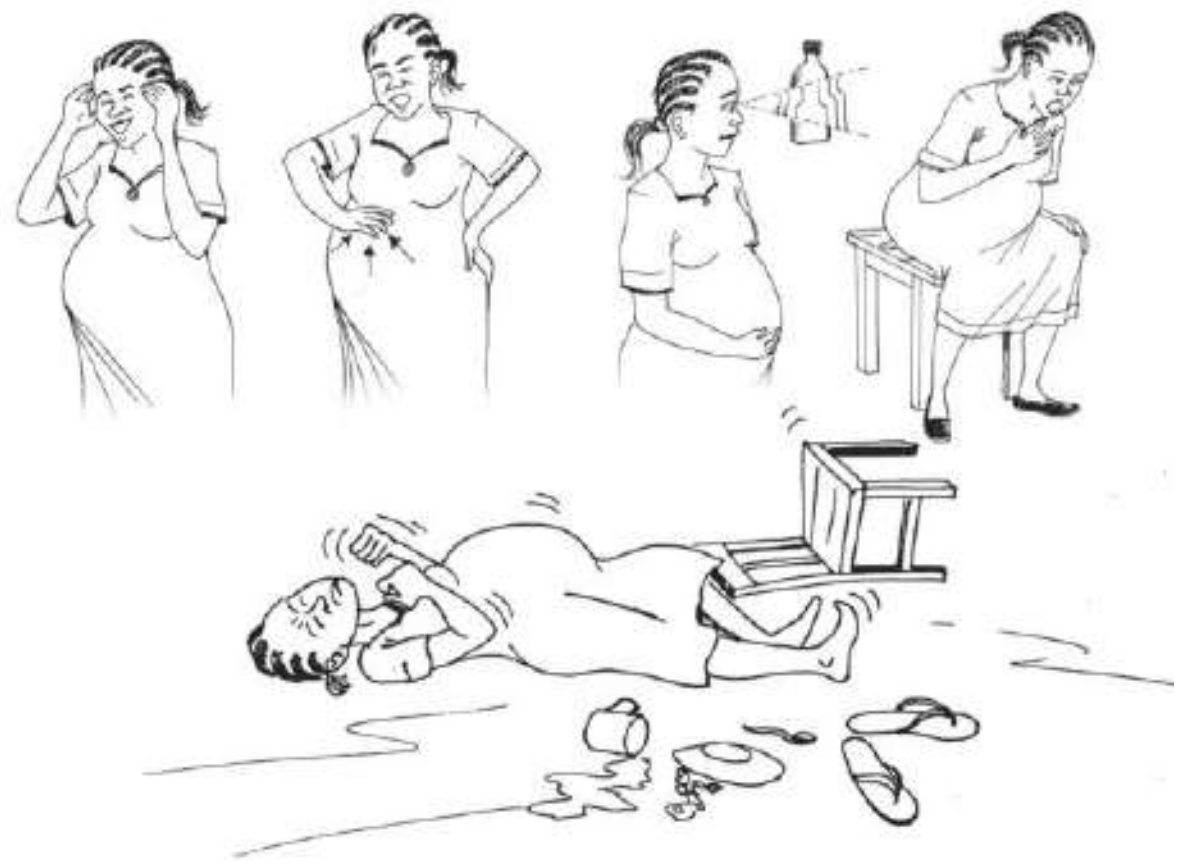

DAMU AU MAJI MAJI YANAYONUKA UKENI WAKATI WA UJAUZITO

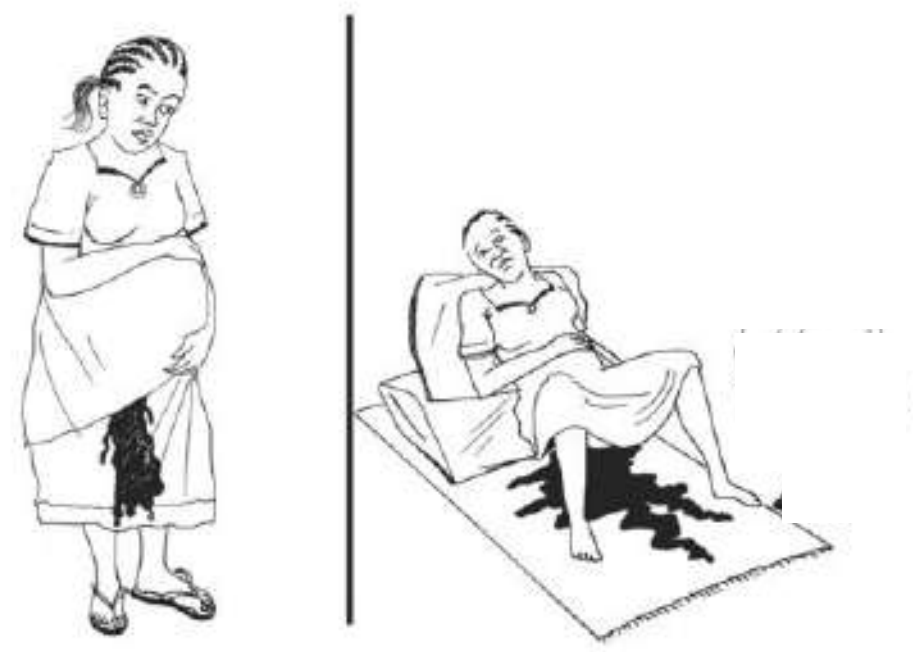

KUJIFUNGUA KABLA YA WAKATI/NJITI

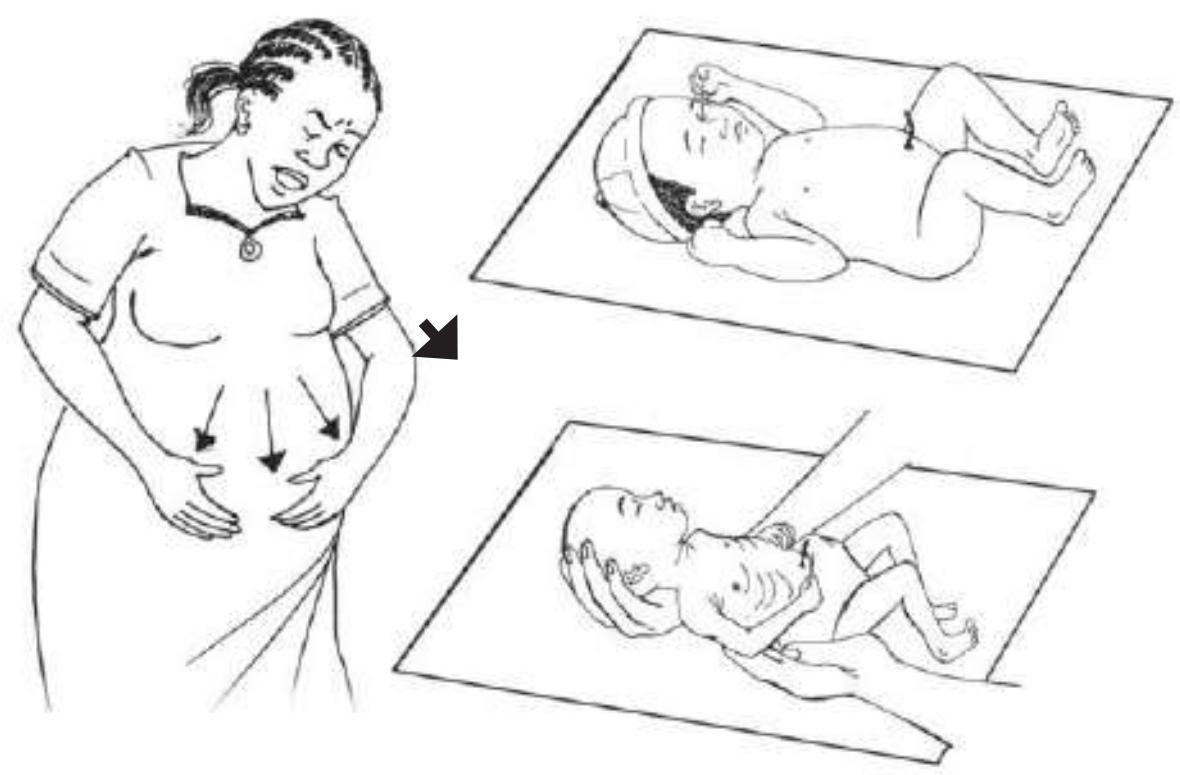

MAAMBUKIZI NA MAGONJWA

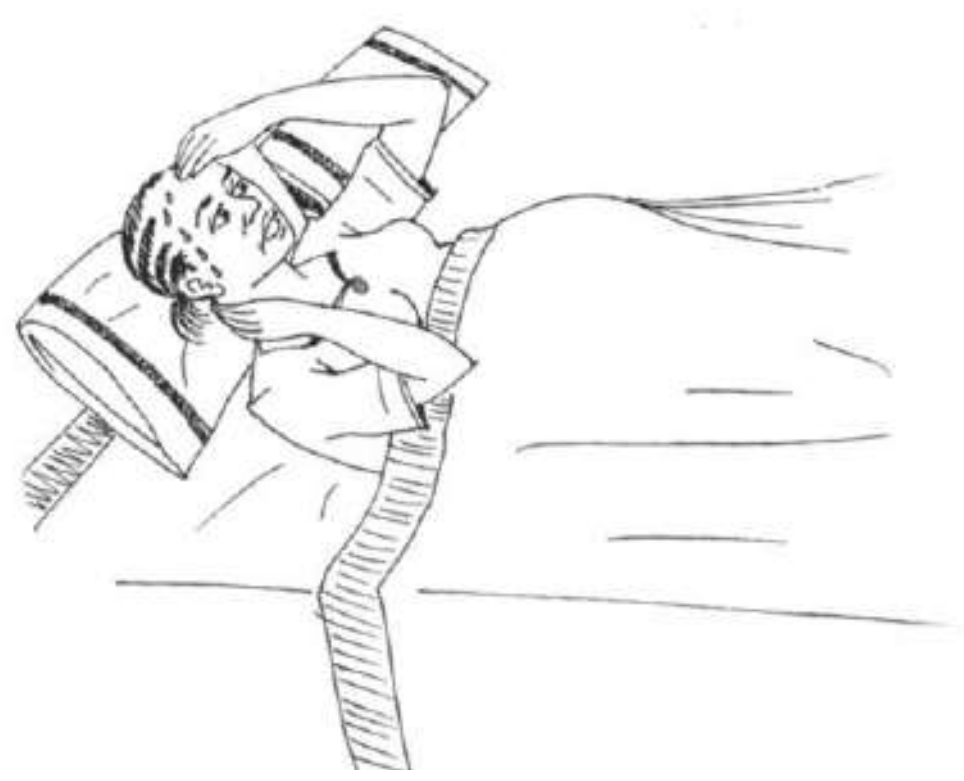

MAUMIVU YA MARA KWA MARA WAKATI WA KUKOJOA AU KUTOKWA MAJIMAJI YENYE HARUFU MBAYA UKENI

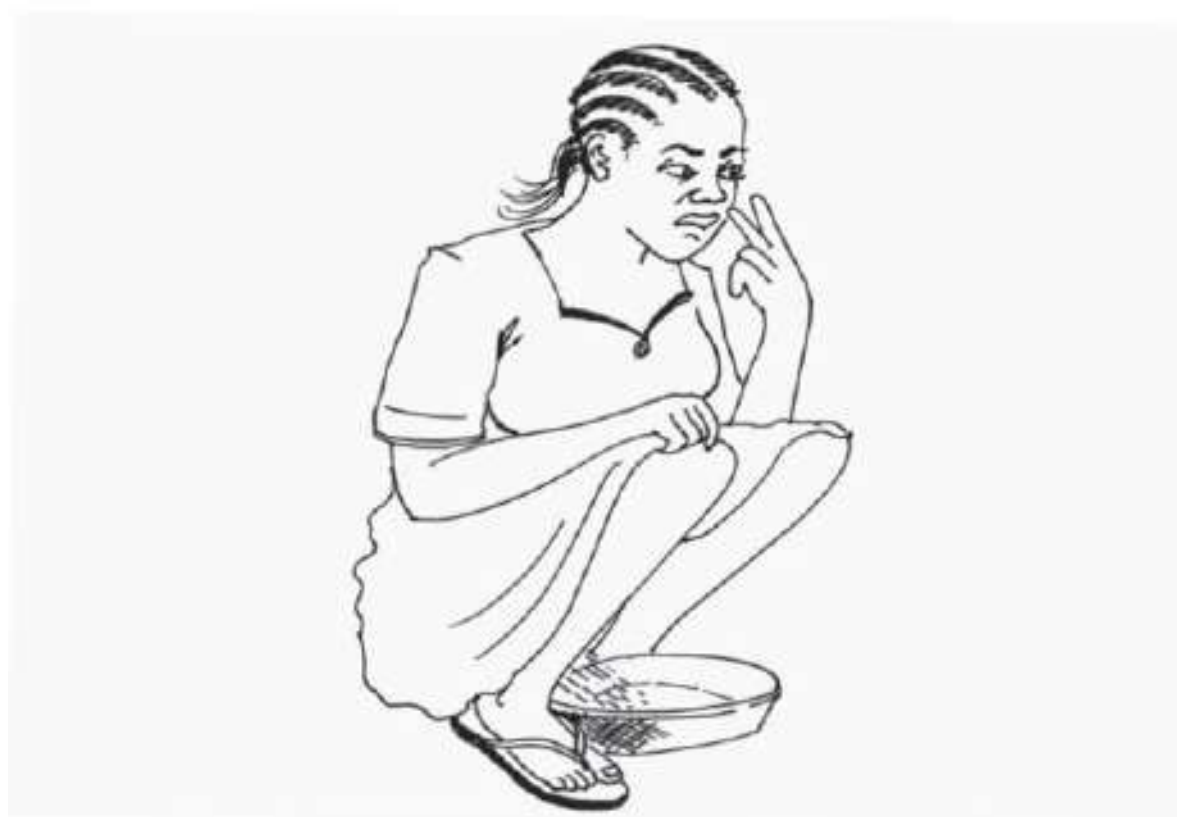

UNYANYASAJI WA KIJINSIA

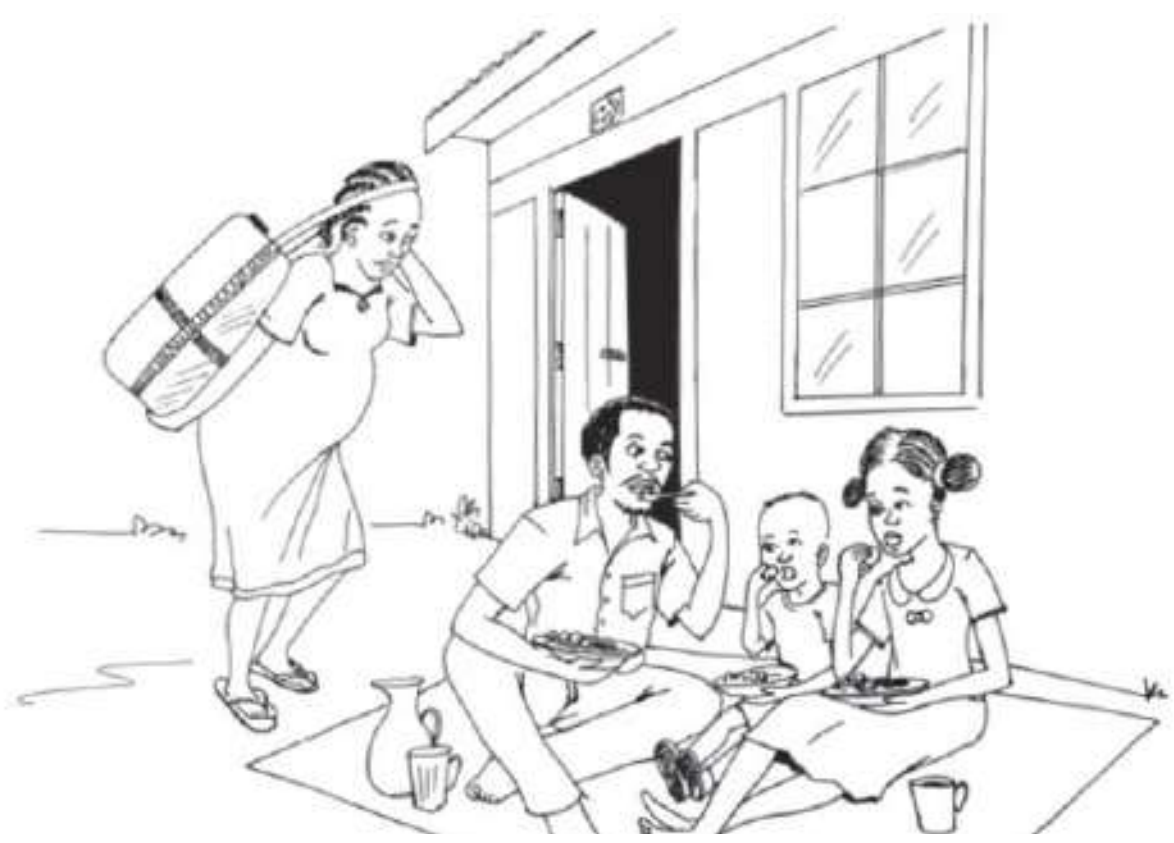

3 Maandalizi kabla ya kujifungua

VITU VYA KUANDAA KWA AJILI YA KUJIFUNGUA

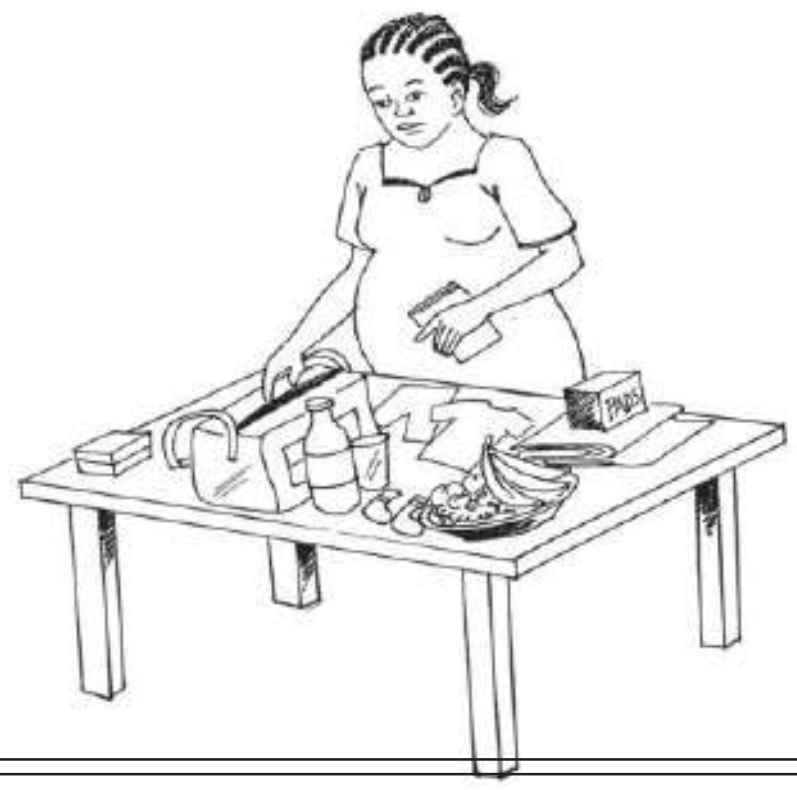

CHAGUA SEHEMU YA KUJIFUNGULIA NA ANDAA MTU WA KUKUSINDIKIZA KITUO CHA AFYA

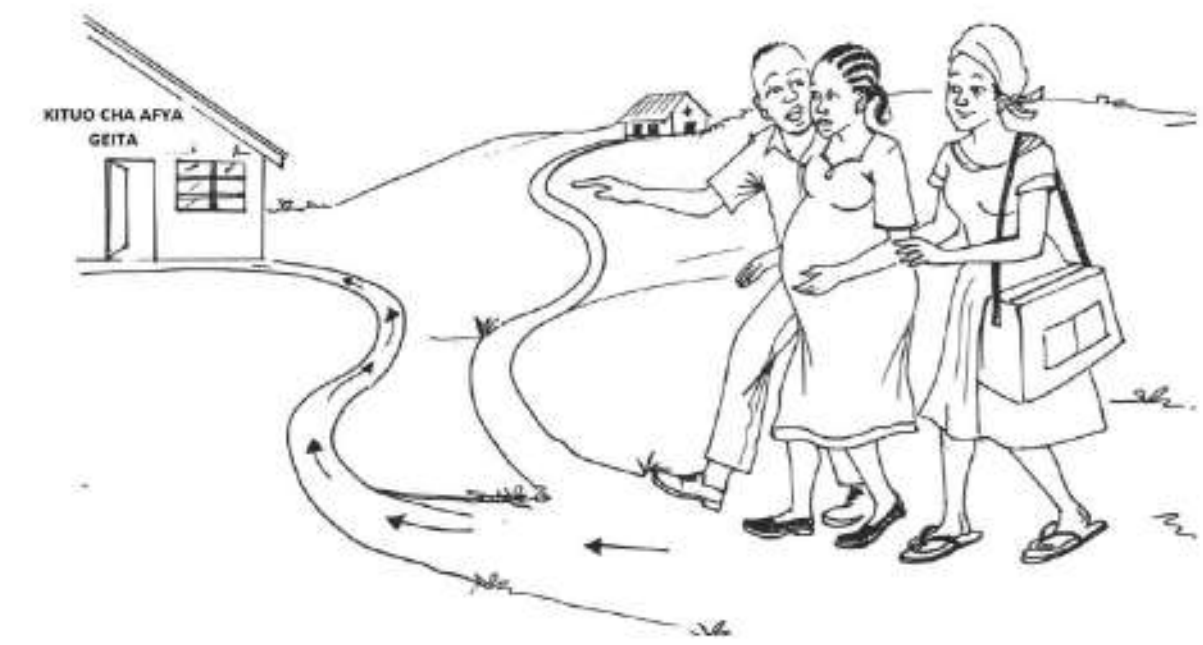

TUNZA HELA KWA AJILI YA KUJIFUNGUA NA MAHITAJI MENGINE

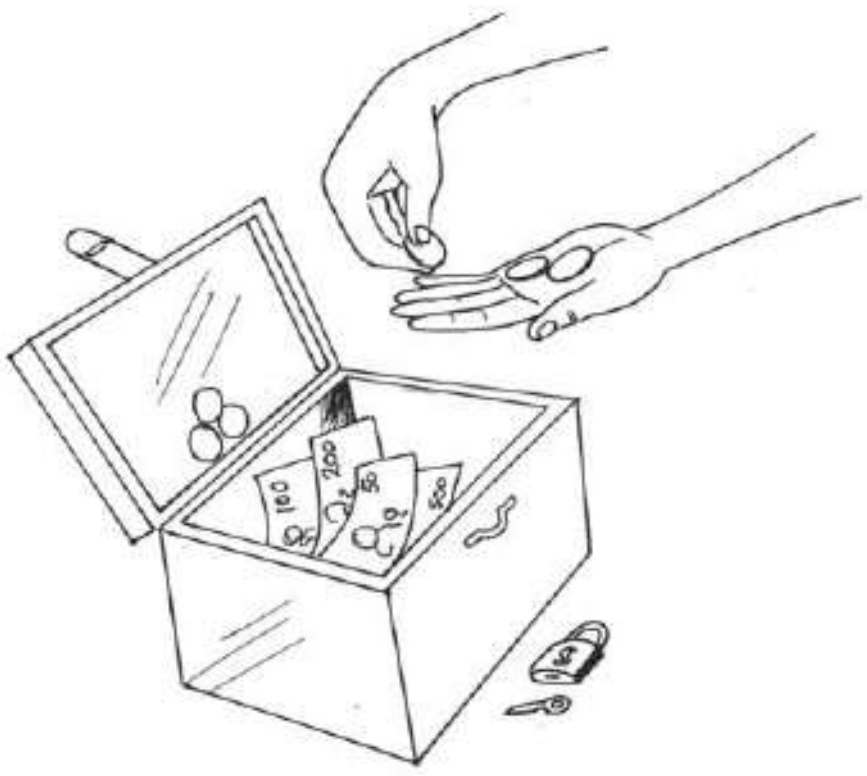

ANDAA USAFIRI WA KUKUPELEKA KITUO CHA AFYA MUDA WOTE

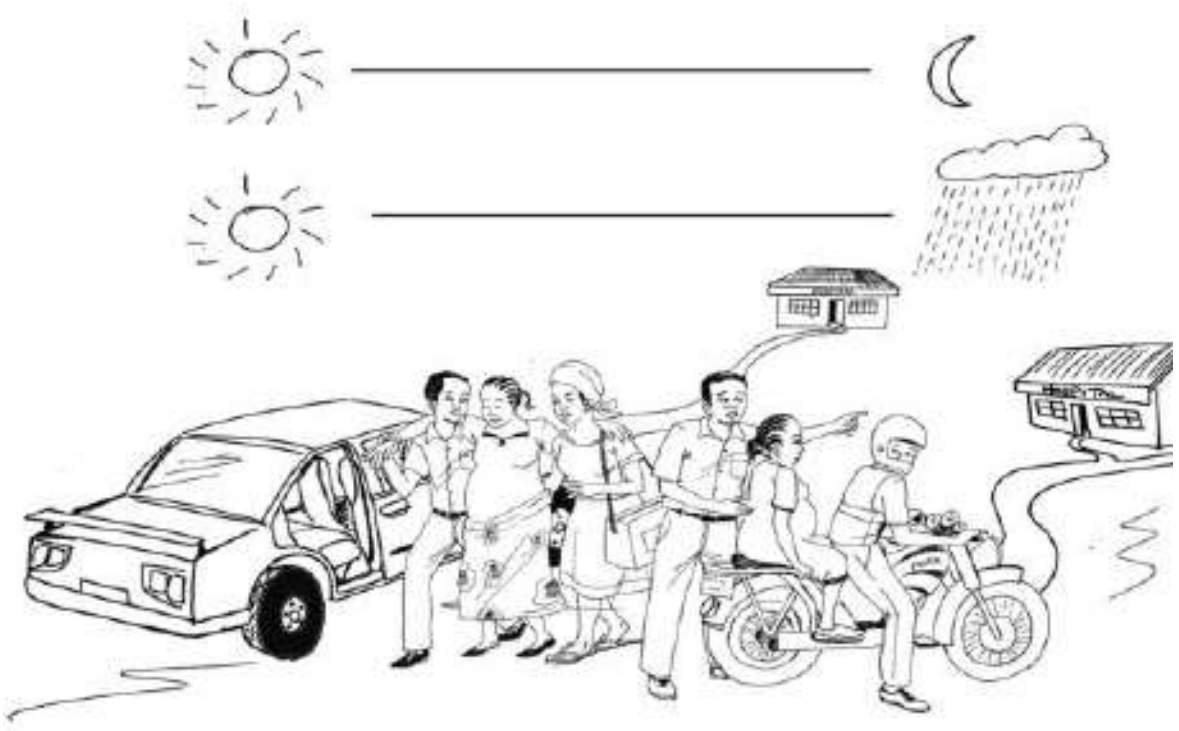

WAFUNDISHE FAMILIA NA WANOKUZUNGUKA KUHUSU DALILI ZA HATARI WAKATI WA UJAUZITO

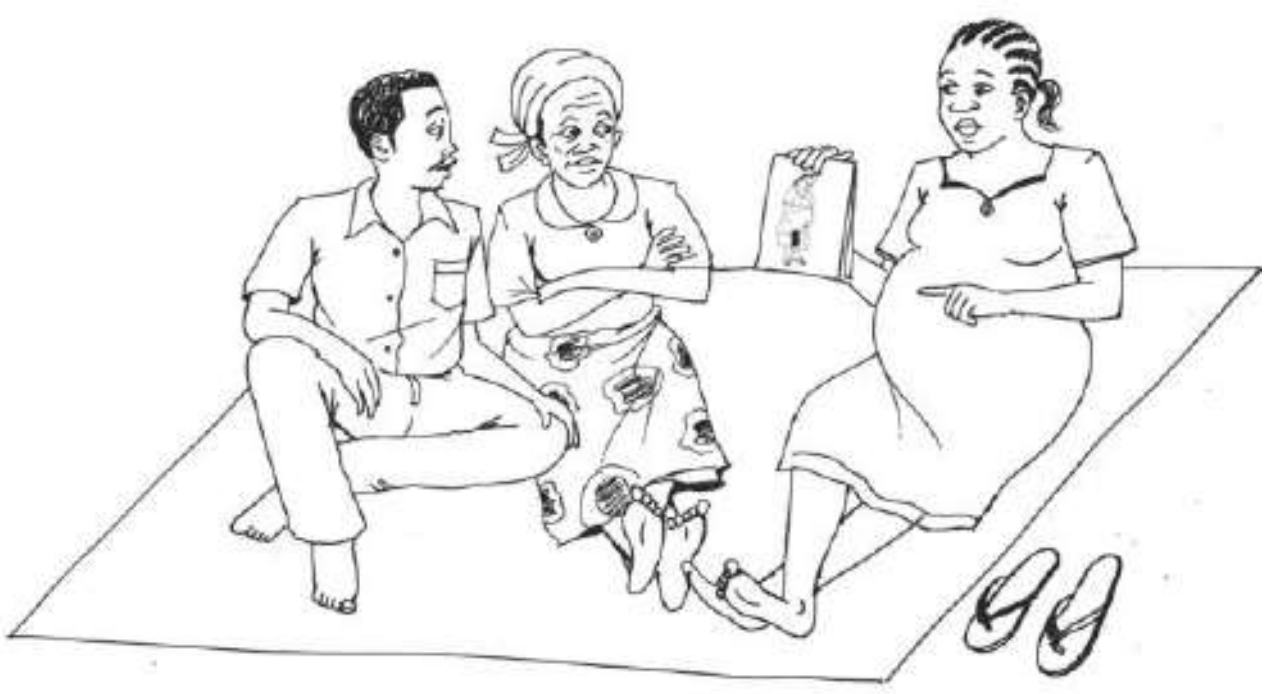

UZAZI WA MPANGO

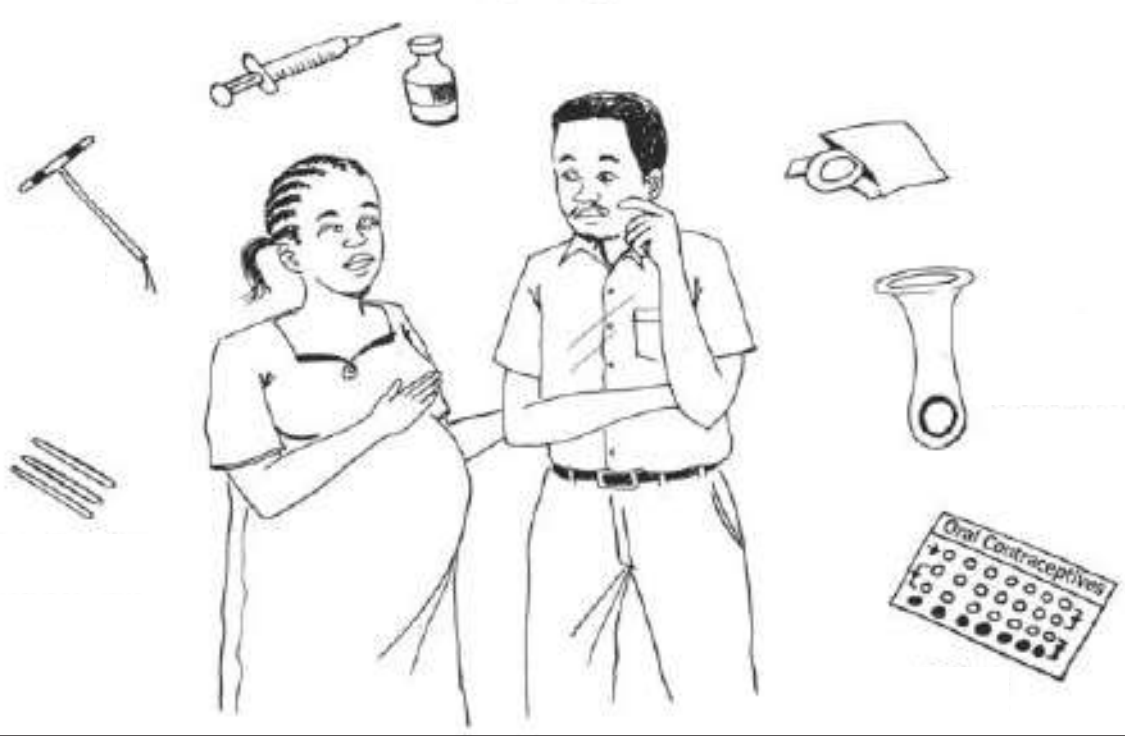

KUWA NA WATOTO WENGI AU KUBEBA MIMBA MARA KWA MARA

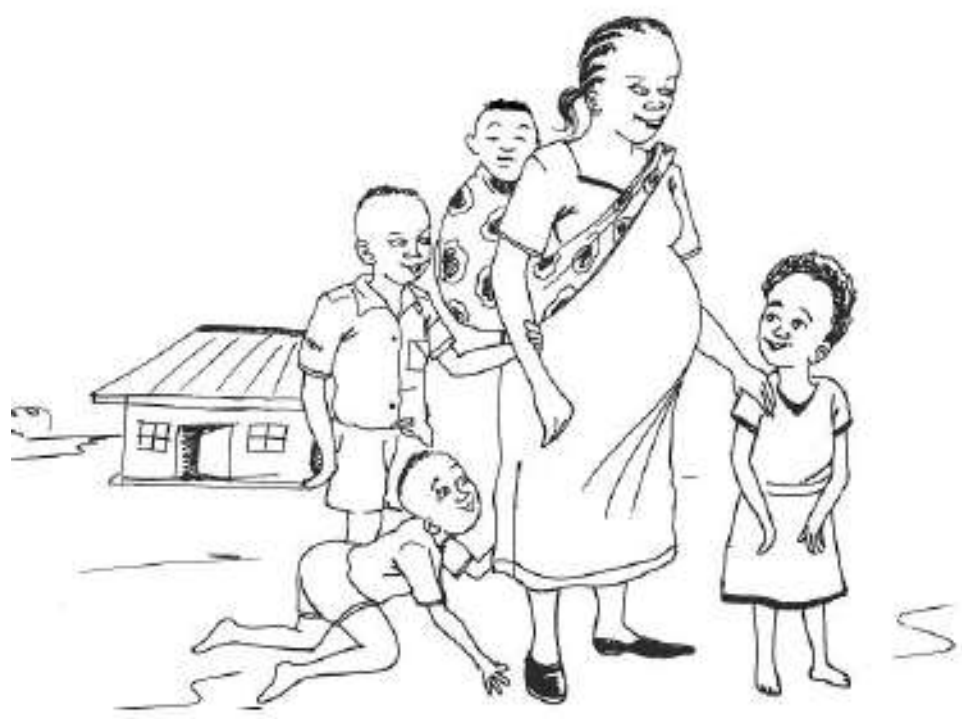

4a Kuzuia matatizo ya kiafya baada ya kujifungua

NYONYESHA MARA BAADA YA KUJIFUNGUA NA MARA NYINGI.
NYONYESHA MAZIWA YA MAMA PEKEE KWA MUDA WA MIEZI 6

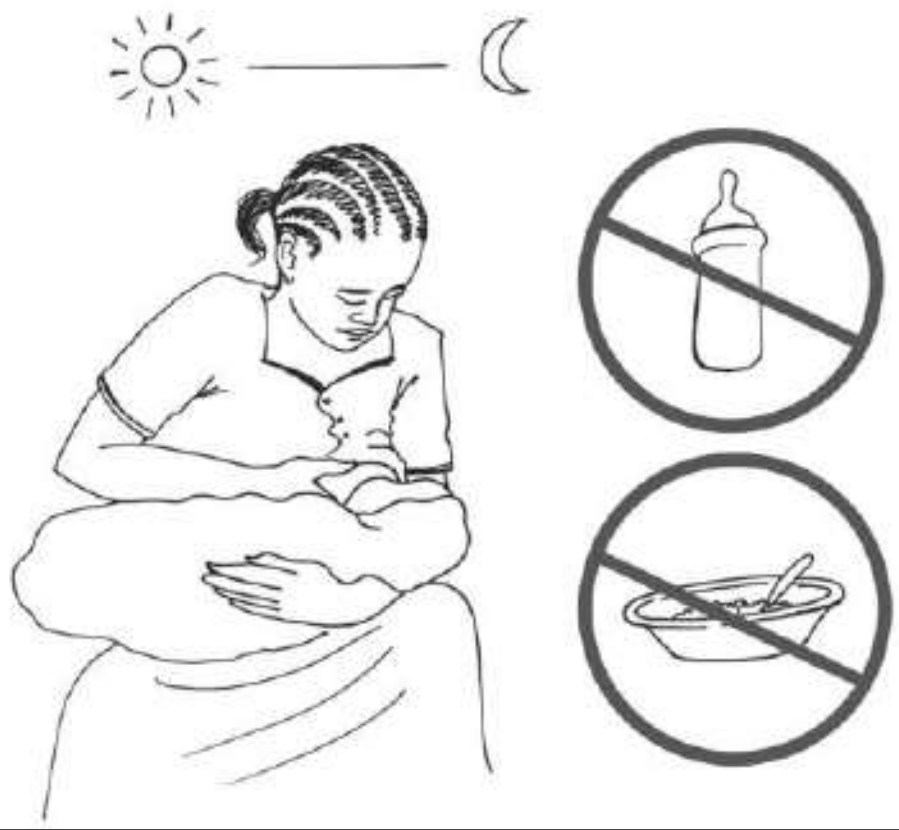

MWEKE MTOTO KATIKA HALI YA JOTO KWA KUMFUNIKA VIZURI KWA NGUO NZITO

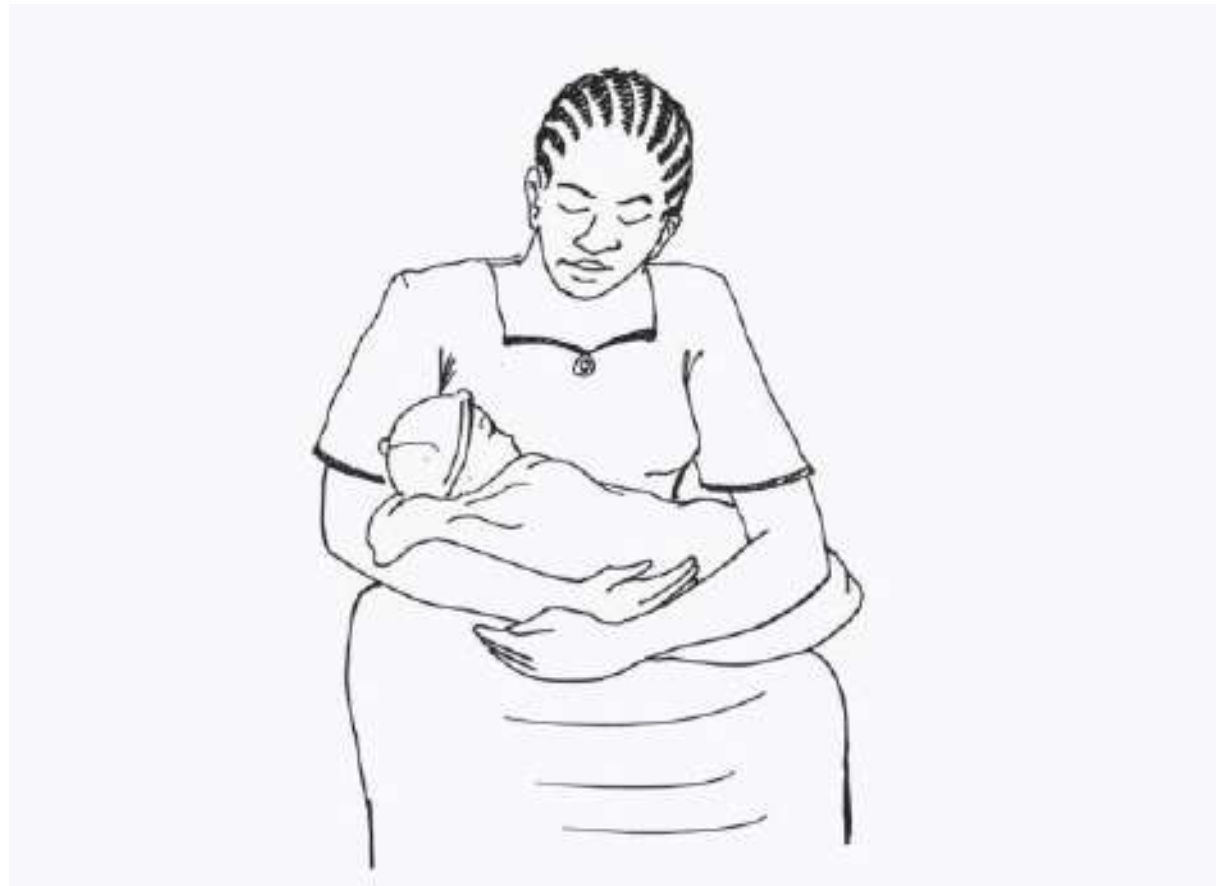

MWEKE MAMA, MTOTO NA KITOVU KATIKA HALI YA USAFI

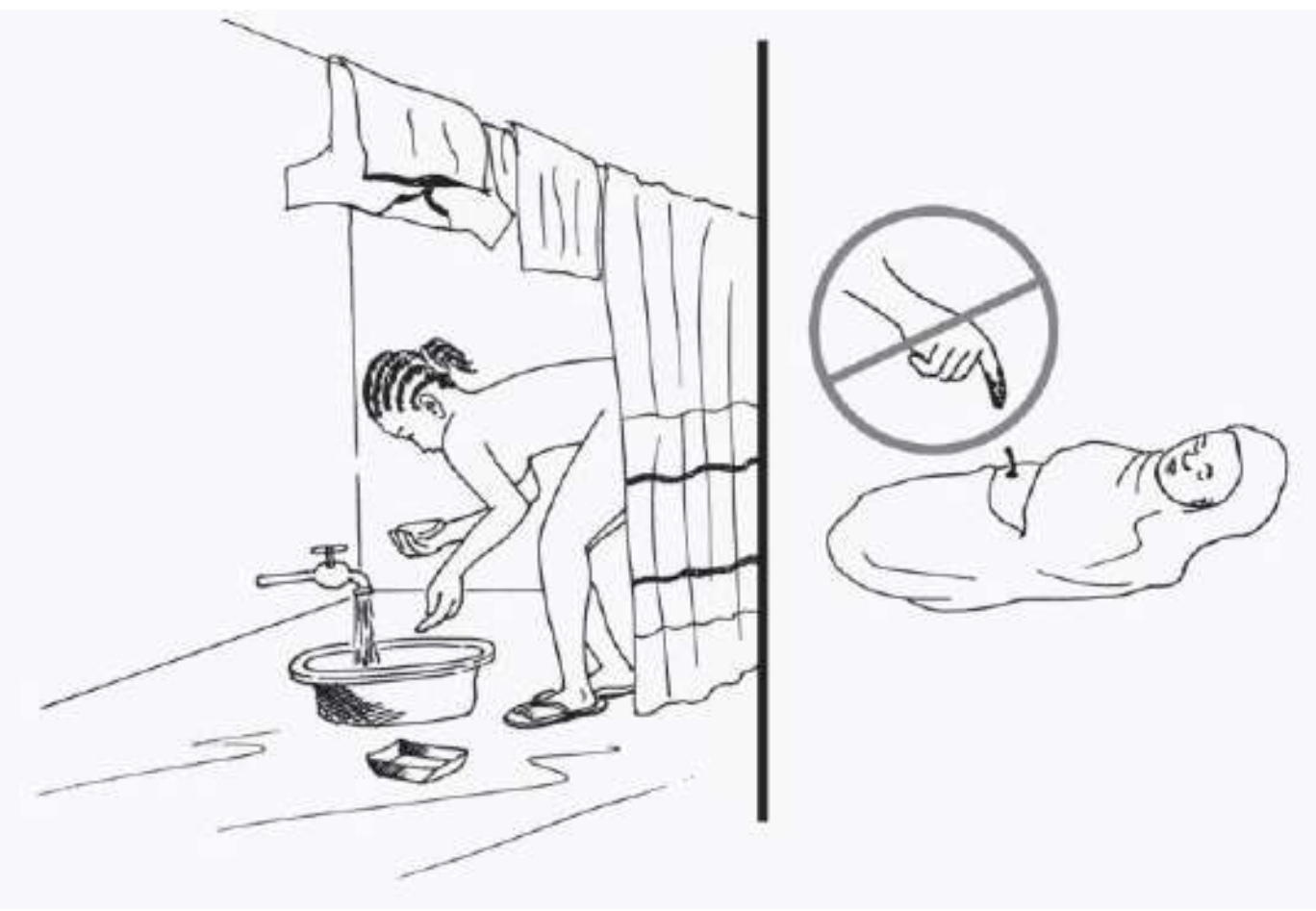

KUNYWA VIMIMINIKA VINGI.ANGALAU MARA 4 KWA SIKU.
MEZA VIDONGE VYA MADINI CHUMA NA FOLIC ACID KWA MUDA WA MIEZI 3

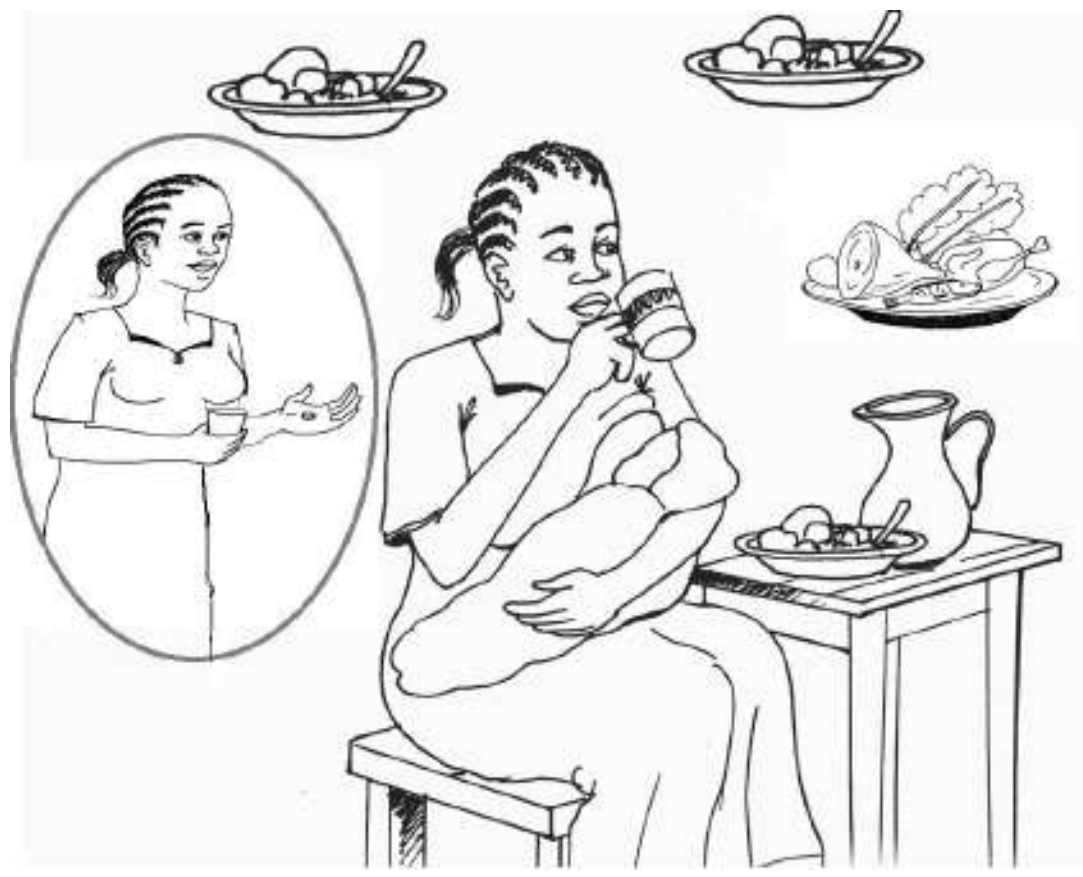

USIFANYE KAZI NGUMU AU KUBEBA VITU VIZITO KWA MUDA WA WIKI 2. MAMA NA MTOTO WALALE KWENYE CHANDARUA.

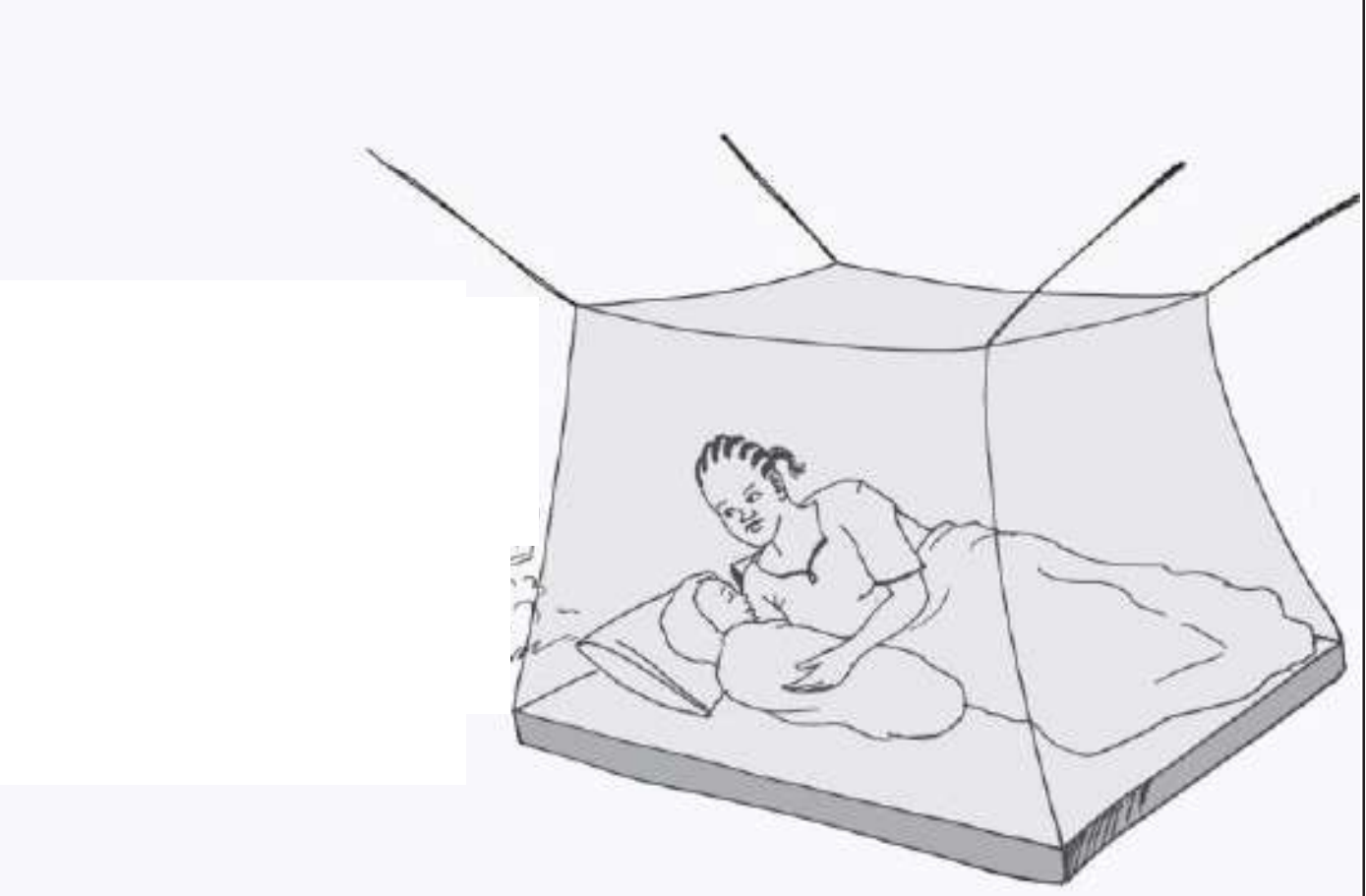

FANYA UCHUNGUZI WA MATATIZO BAADA YA KUJIFUNGUA. NENDA KLINIKI YA MAMA NA MTOTO ANGALAU MARA 4 NDANI YA SIKU 42 BAADA YA KUJIFUNGUA. SIKU 3-7, 8-28, 8-28, 29-42.

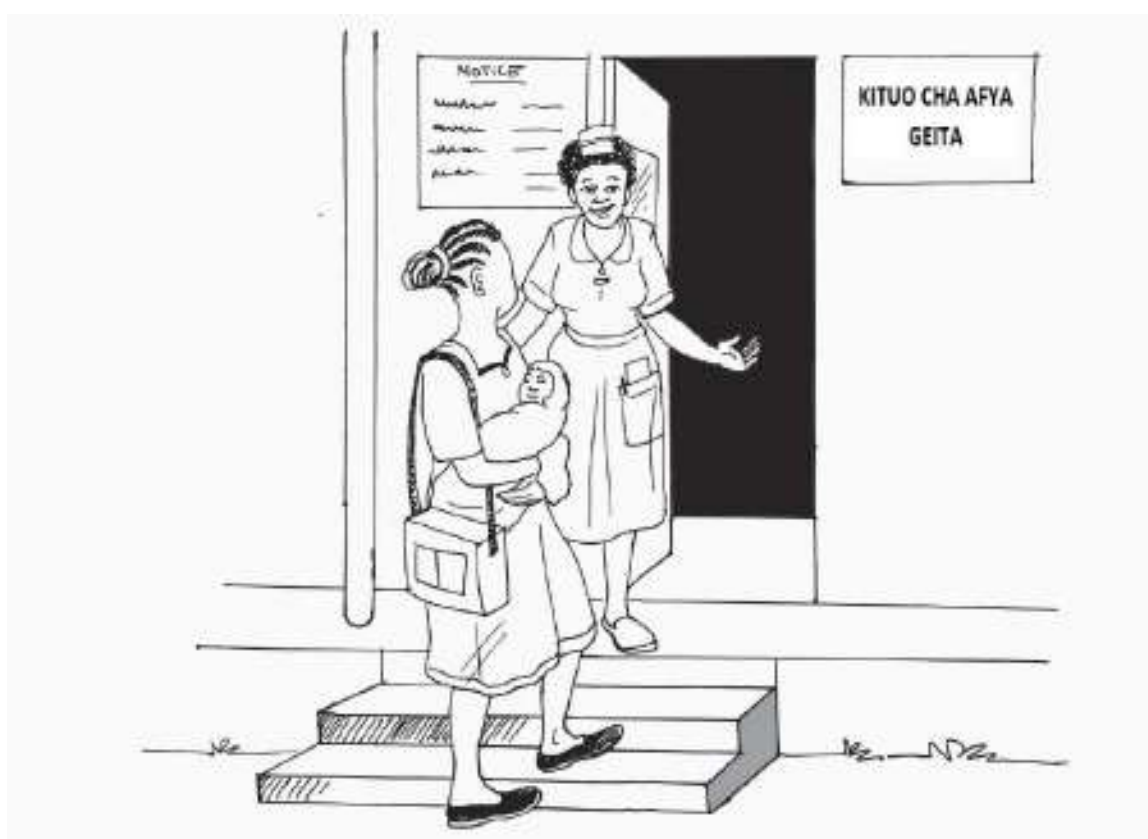

4b Wasiyohitaji watoto wengine tena: njia za kudumu

KUFUNGA MIRIJA YA UZAZI KWA MWANAMKE

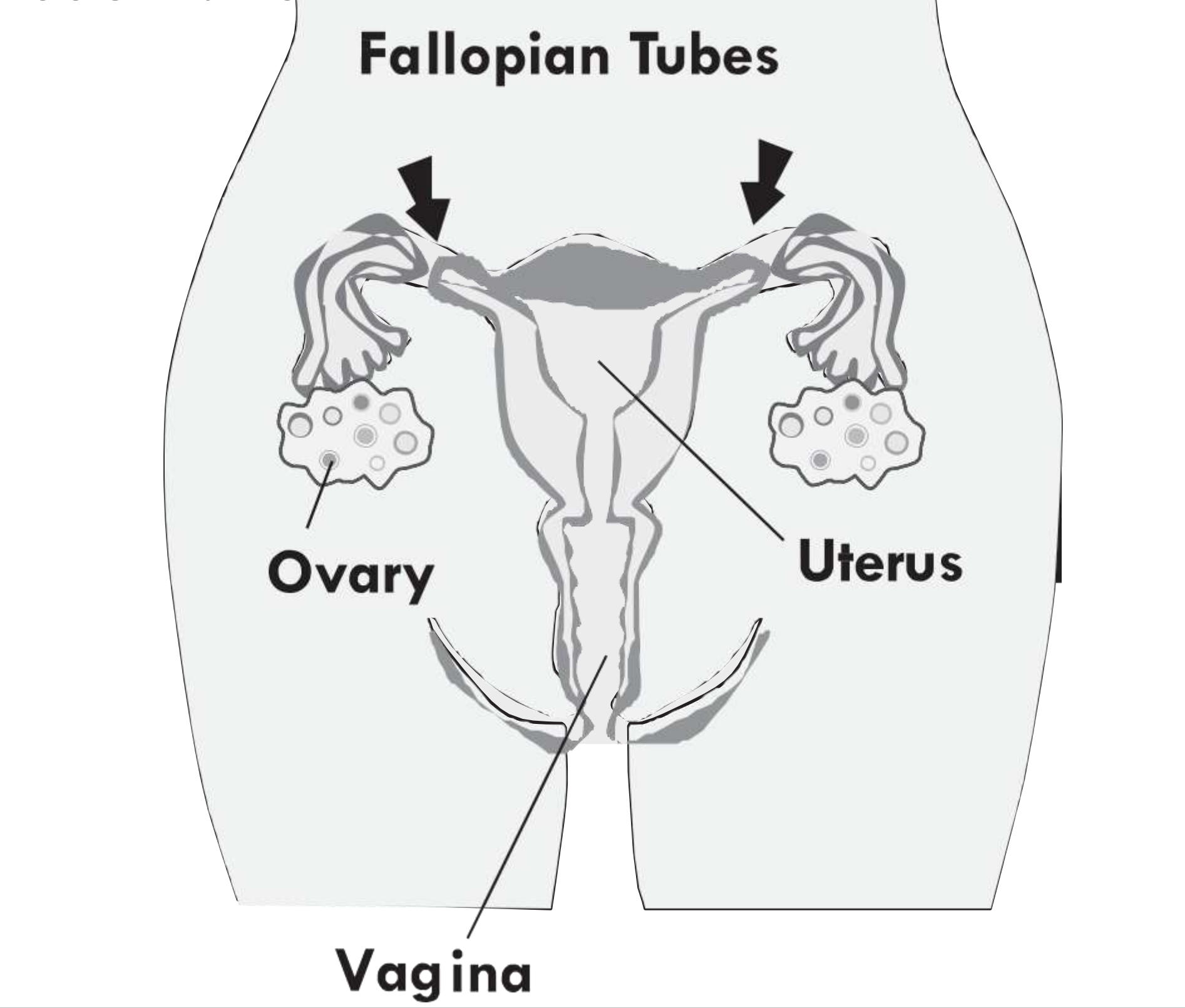

KUFUNGA UZAZI KWA MWANAUME

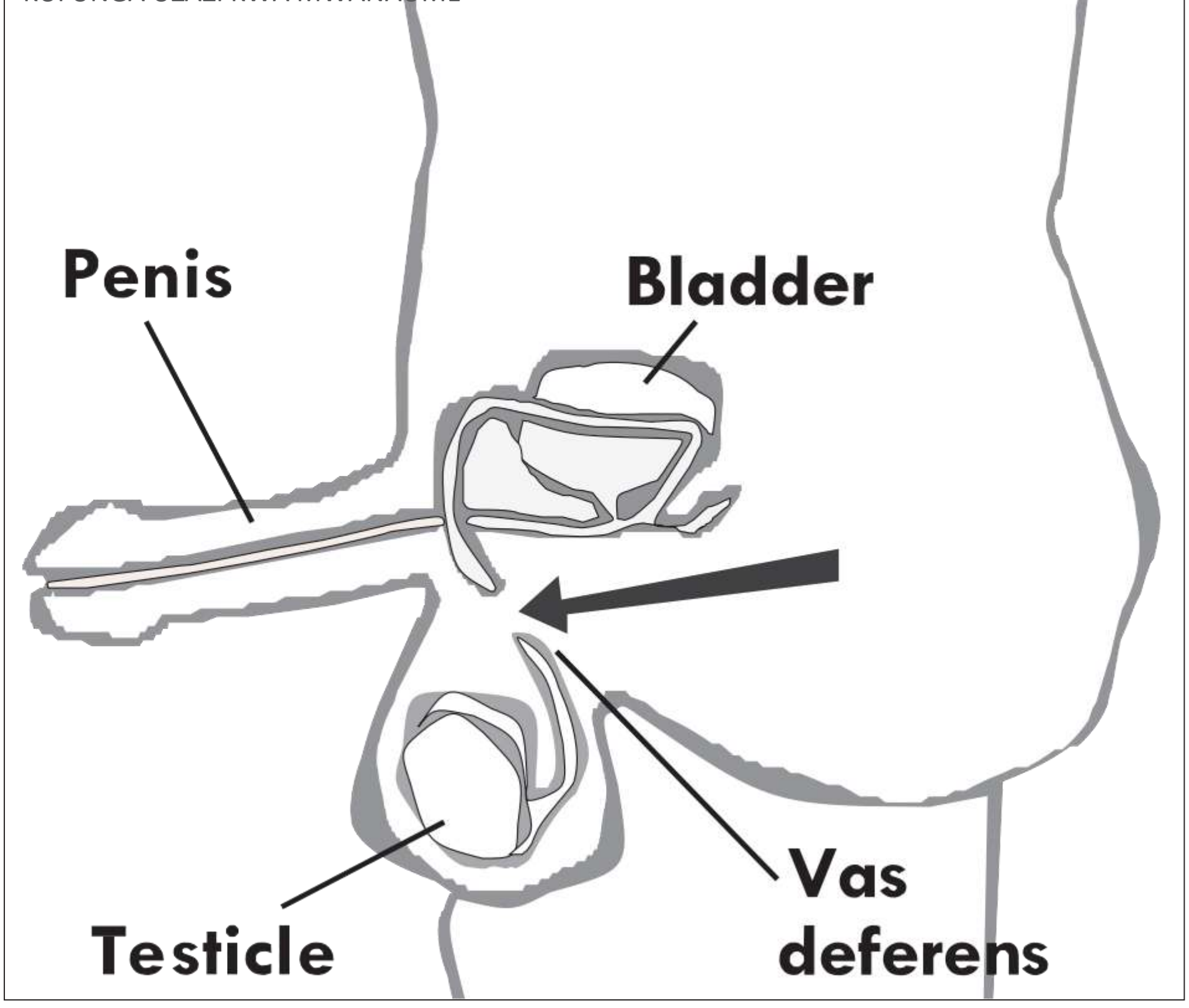

4b Uzazi wa mpango: Matumizi ya Baadae

SINDANO YA KICHOCHEO CHA PROGESTINI (KILA MIEZI 2-3): Njia hii inaweza kutumika mara baada ya kujifungua kama hunyonyeshi na miezi sita baada ya kujifungua kama unanyonyesha

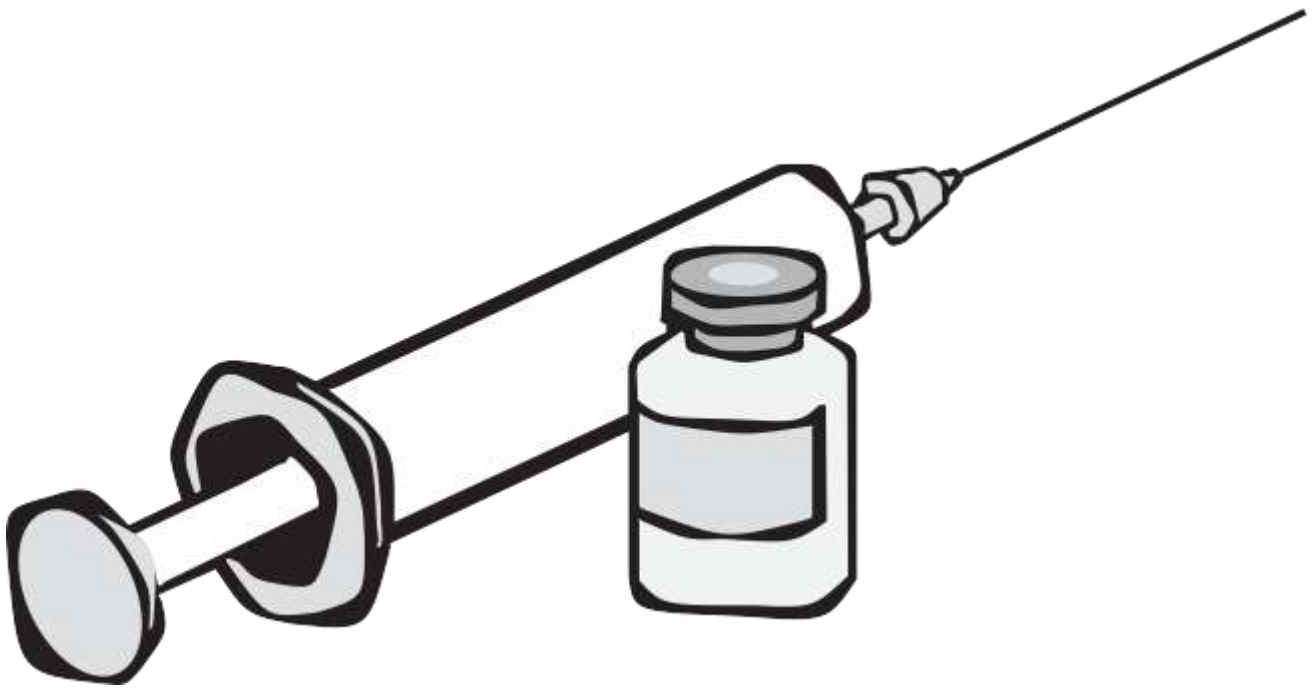

SINDANO ZA KUCHOMA KILA MWEZI: Unaweza kuanza kutumia sindano wiki 3 baada ya kujifungua kama haunyonyeshi ; na baada ya miezi 6 kama unanyonyesha.

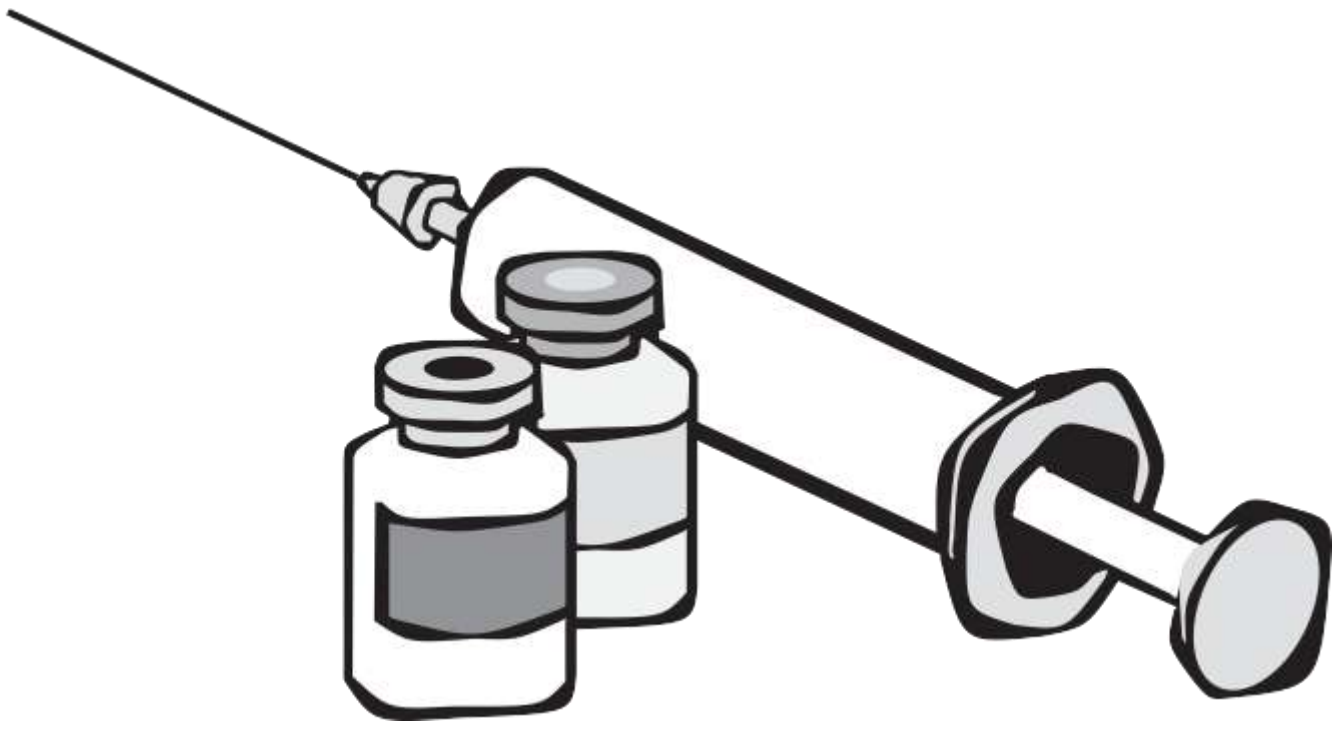

VIDONGEVYA UZAZI WA MPANGO VYENYE VICHOCHEO MCHANGANYIKO: Anza kutumia njia hii baada ya wiki ya tatu kama hunyonyeshi; kama unanyonyesha anza kutumia njia hii miezi 6 baada ya kujifungua.

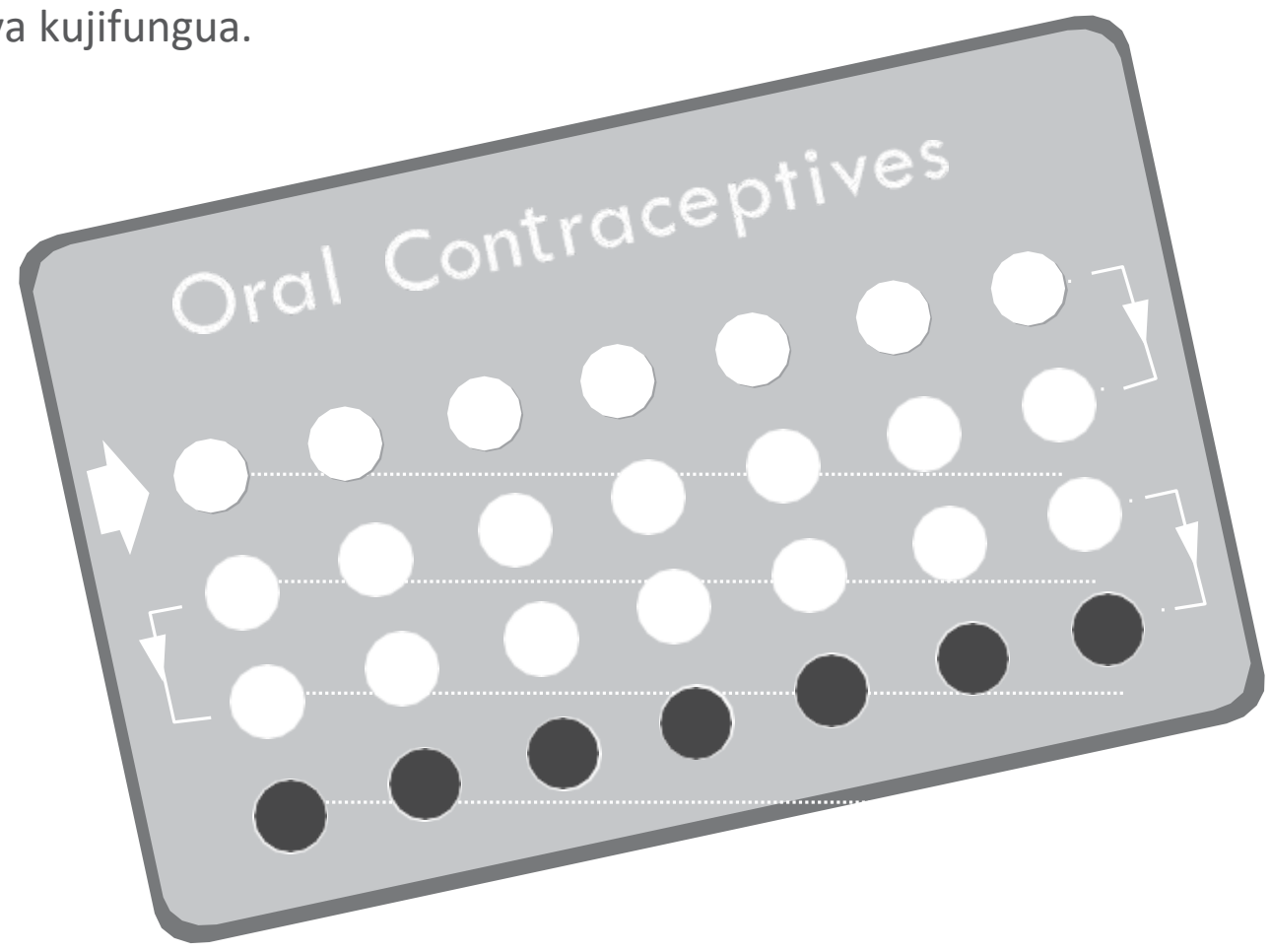

4b

Njia za uzazi wa mpango: Matumizi ya papo hapo baada ya kujifungua.

KITANZI CHENYE KICHOCHEO
KITANZI CHA SHABA

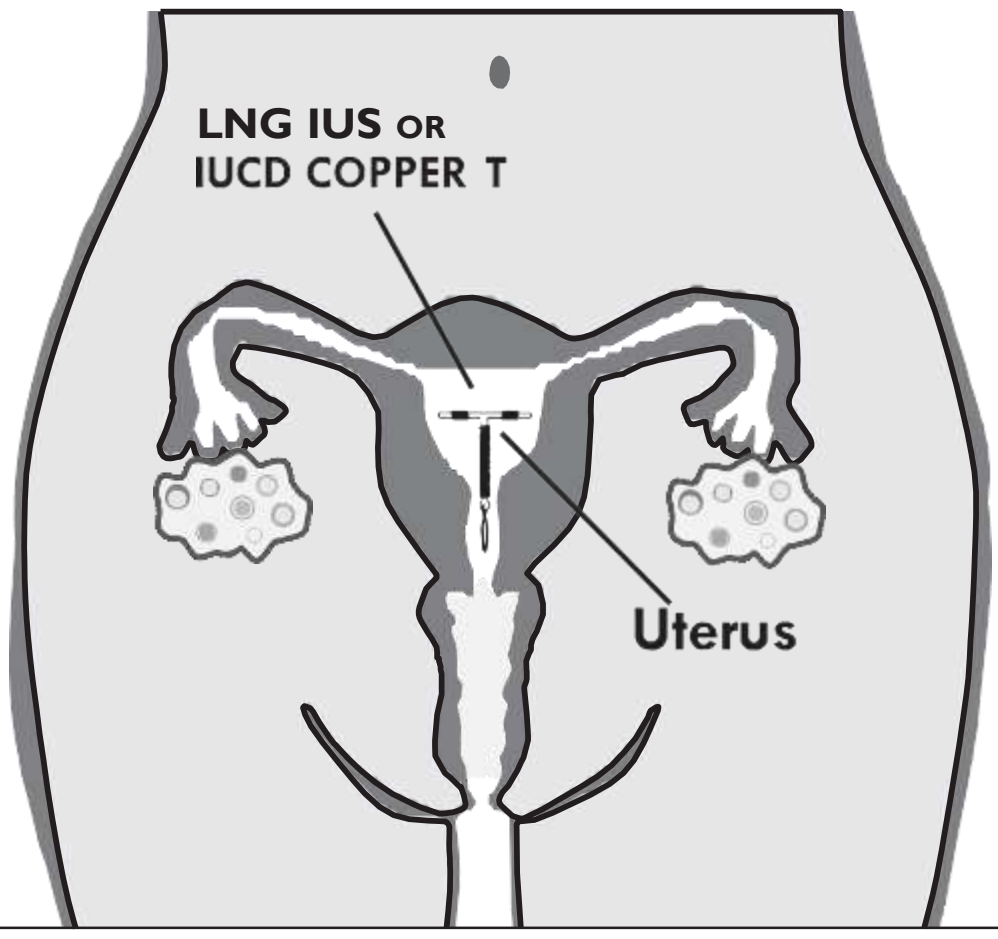

VIPANDIKIZI VYENYE VICHOCHEO

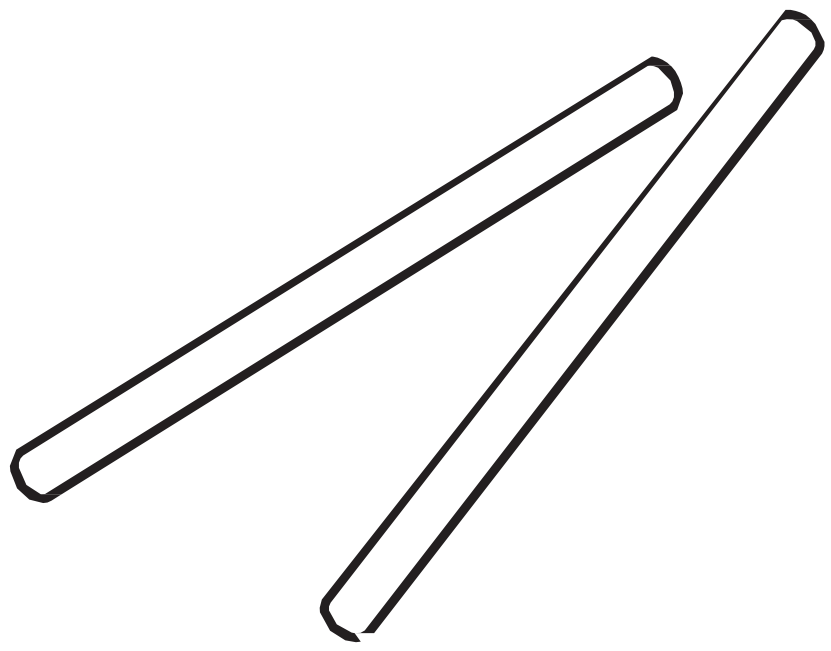

NJIA YA UNYONYESHAJI
MAZIWA YA MAMA

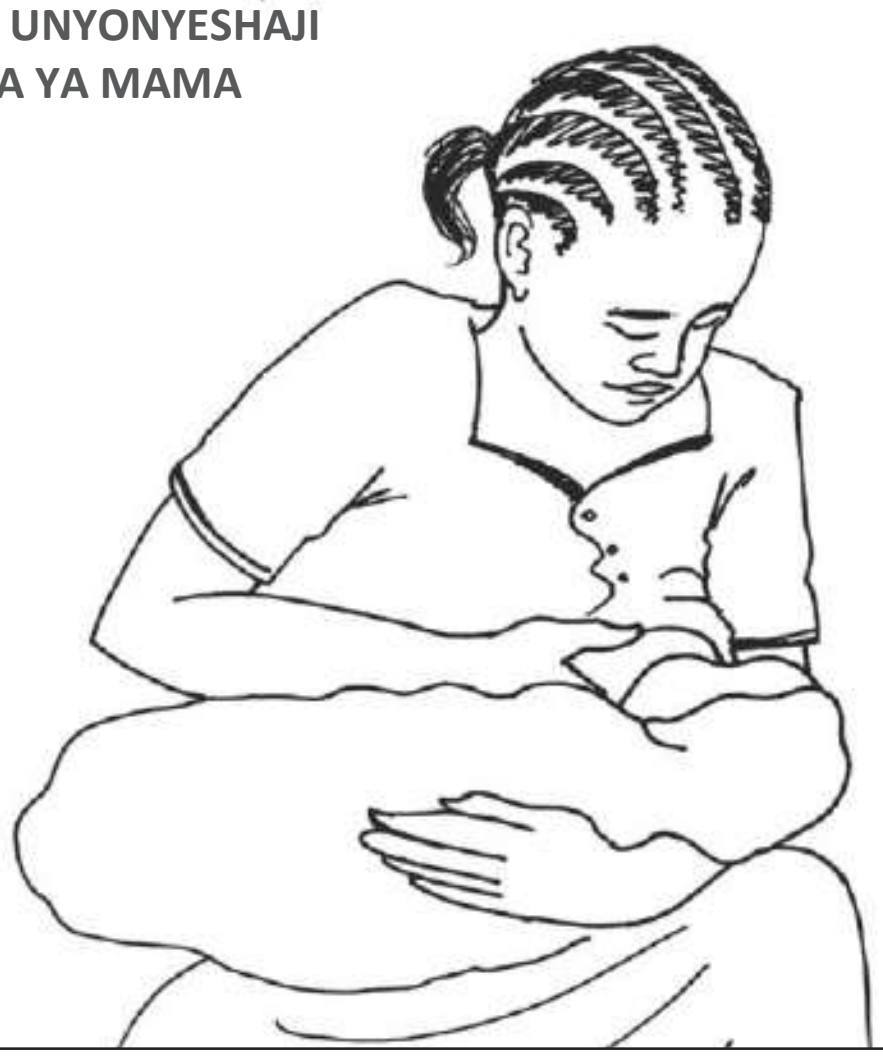

VIDONGE
VYENYE KICHOCHEO
KIMOJA CHA
PROGESTINI

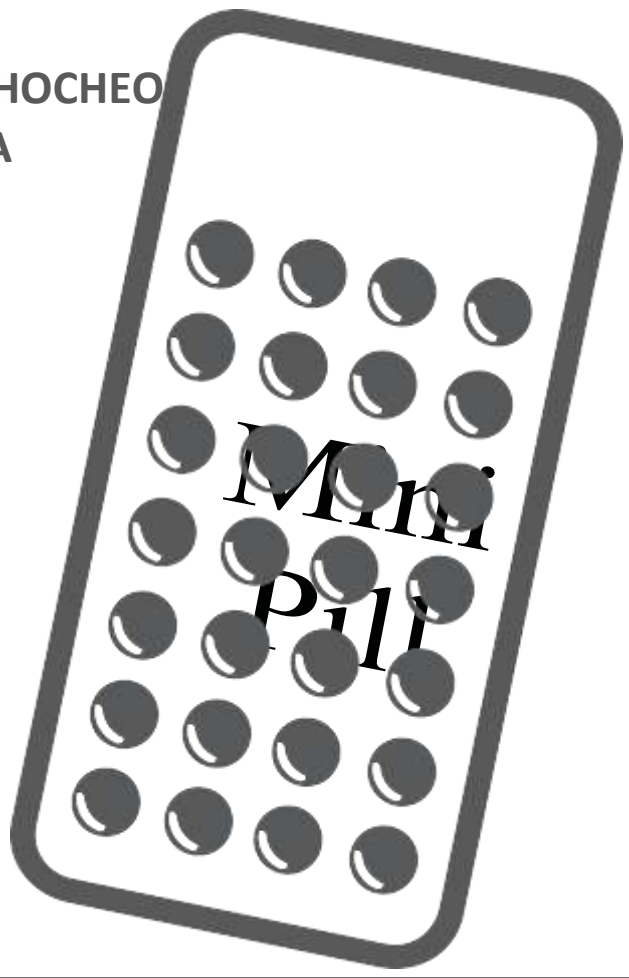

MPIRA WA KIUME / KONDOMU

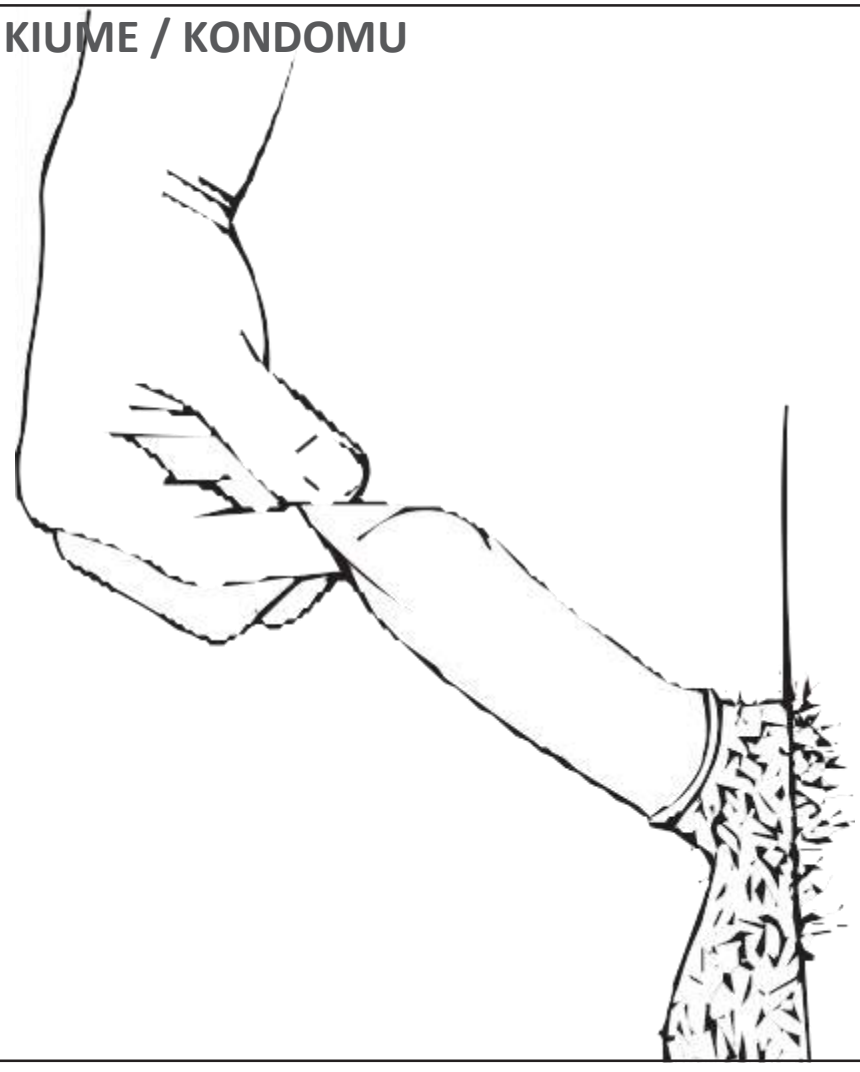

KUCHOMOA UUME KWENYE UKE/ KUMWAGA SHAHAWA NJE YA UKE

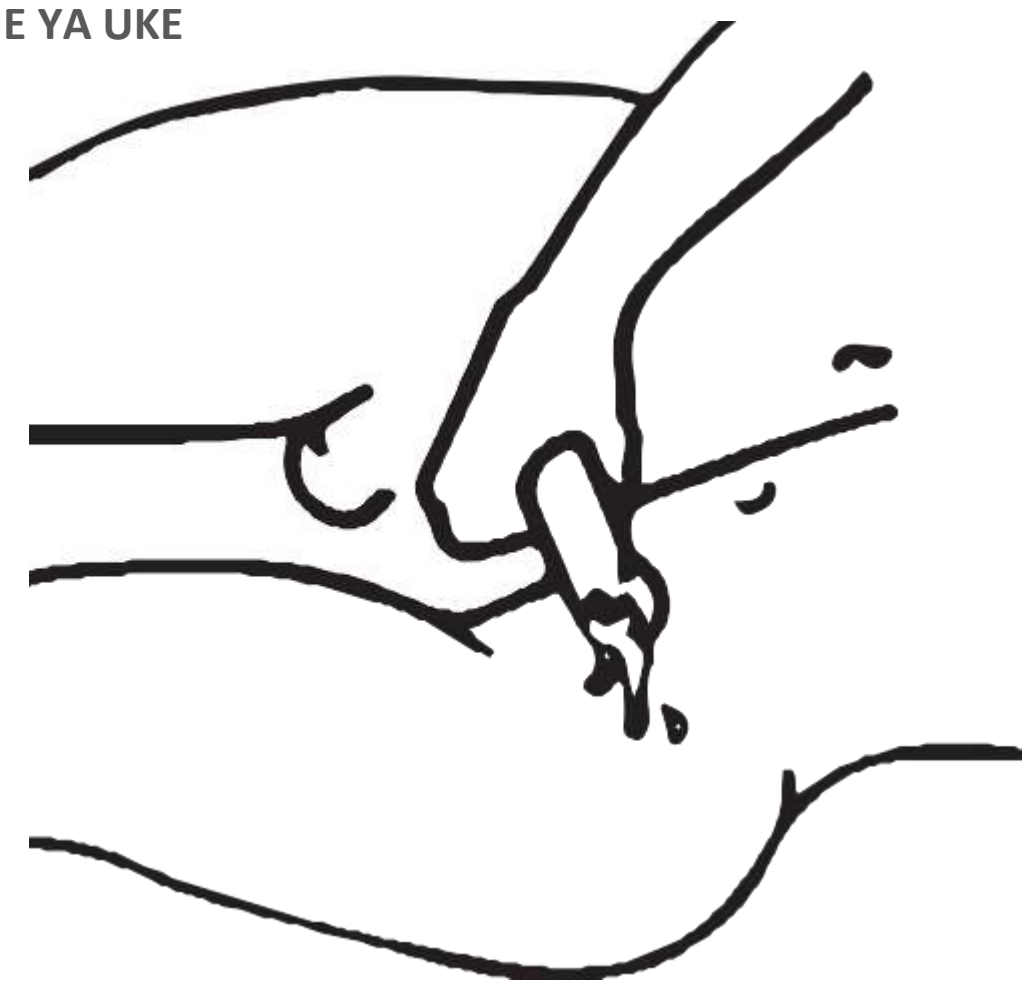

5a Matatizo ya mtoto

KUPUMUA KWA HARAKA AU KUPUMUA KWA SHIDA

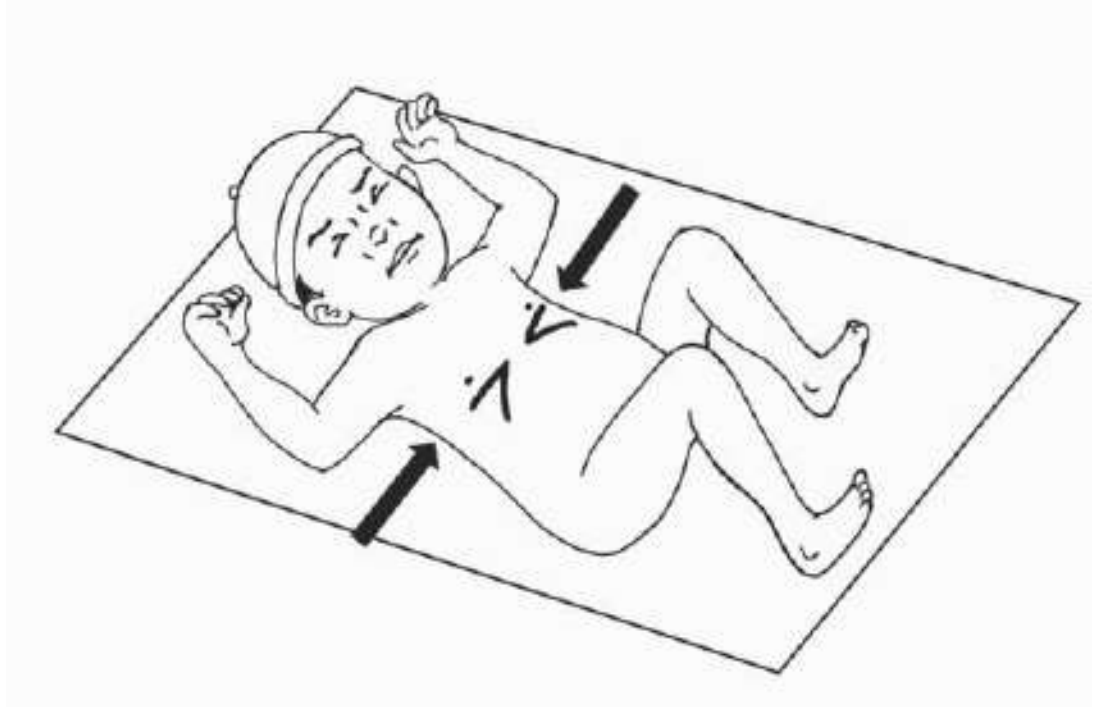

JOTO KALI AU BARIDI SANA

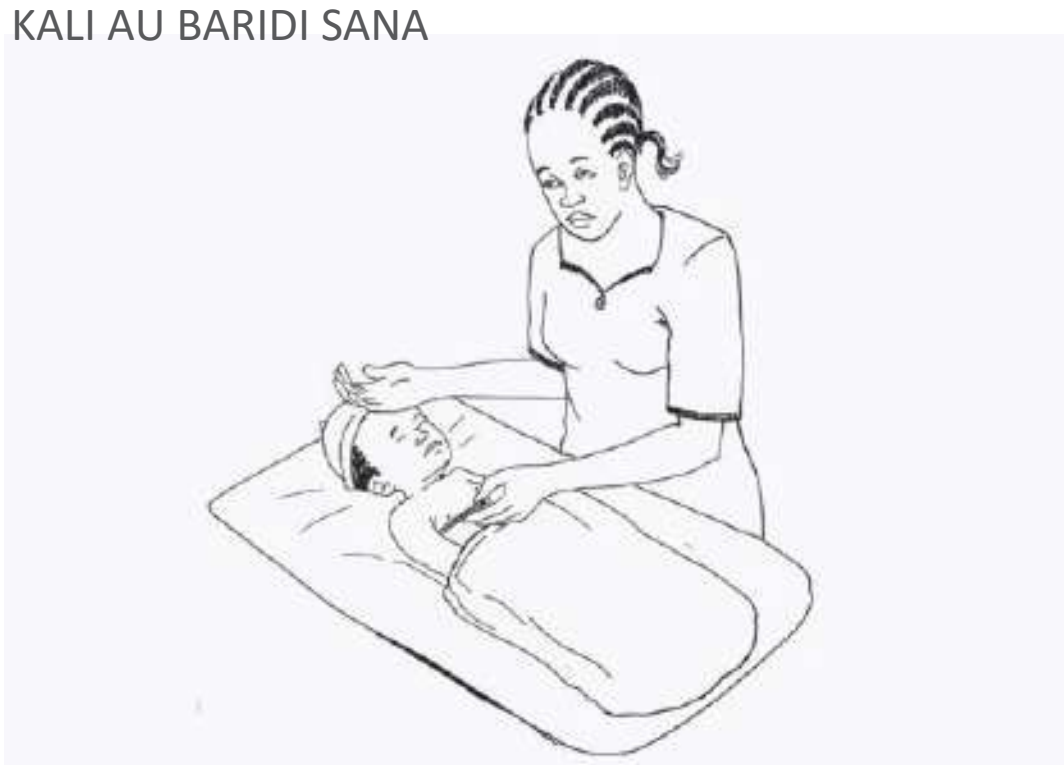

MTOTO ANAHANGAIKA: ANA DEGEDEGE

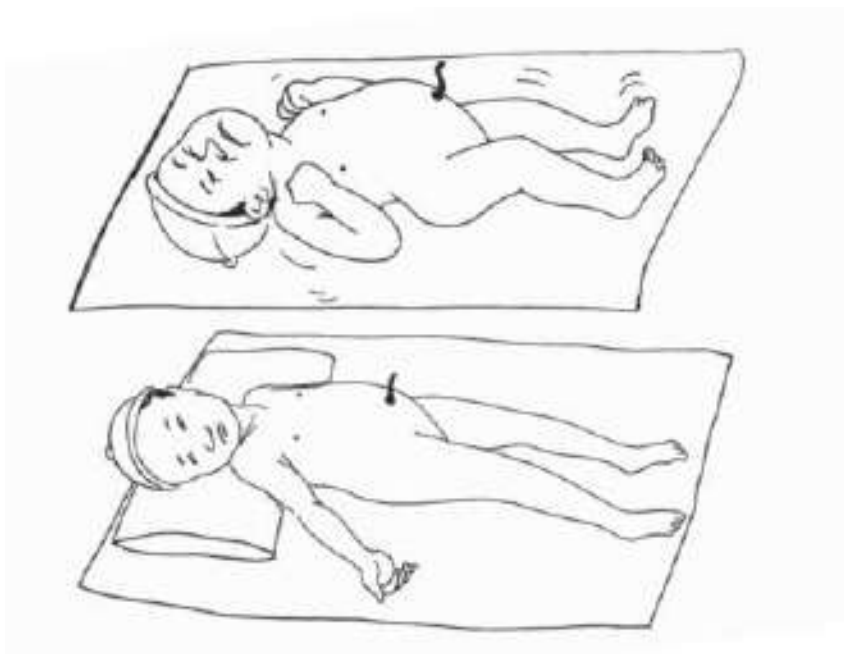

MTOTO AMEACHA KULA / KUNYONYA VIZURI

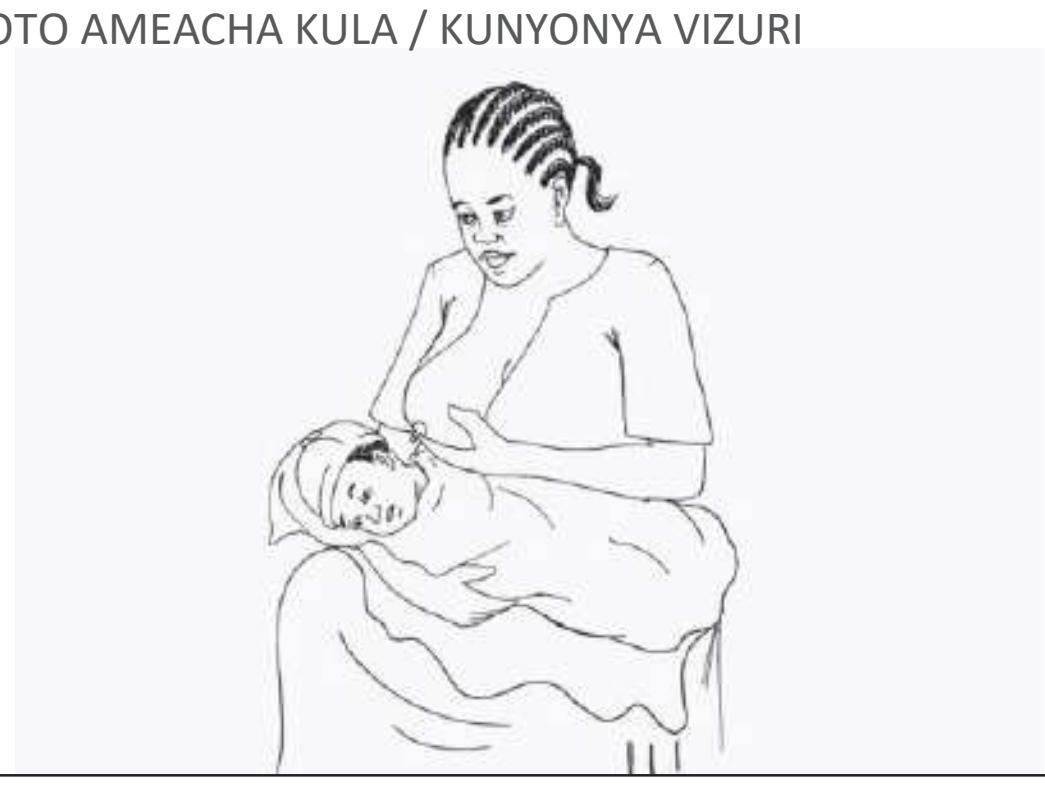

MTOTO NI MDOGO SANA

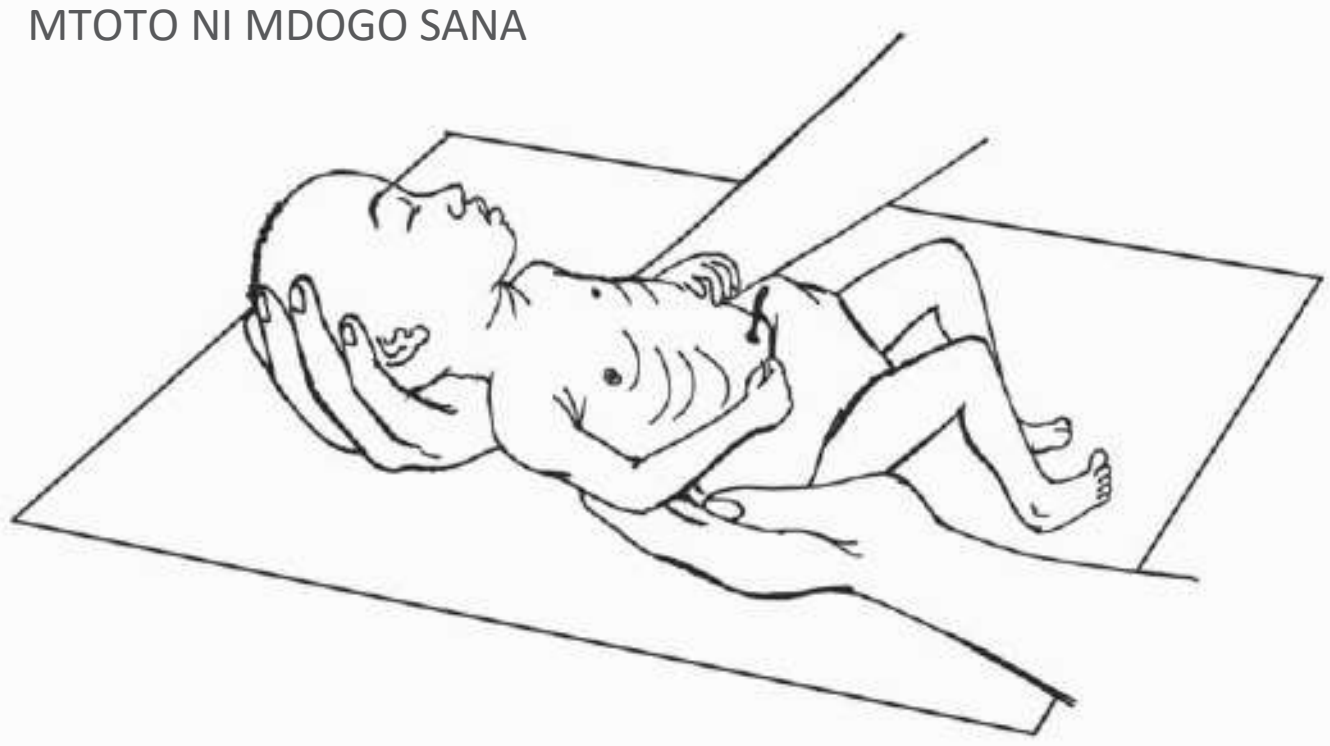

KITOVU NI CHEKUNDU,
AU KINATOA DAMU

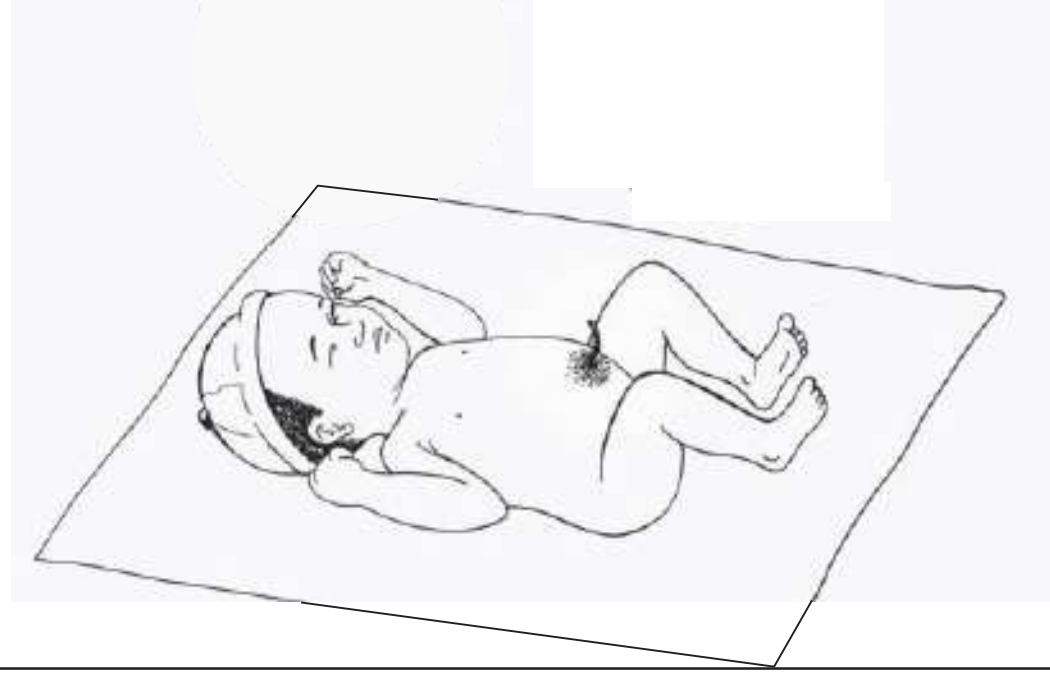

MTOTO NI WA NJANO

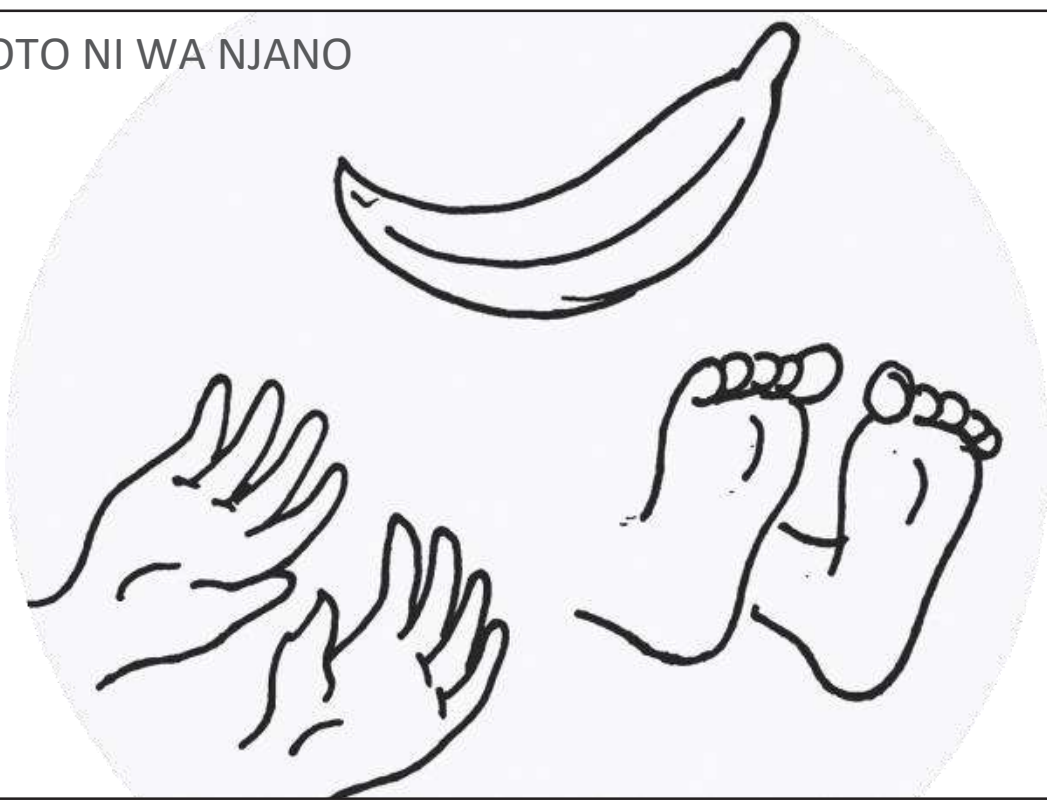

5b Matatizo ya mama baada ya kujifungua

KUKOKWA NA DAMU NYINGI UKENI

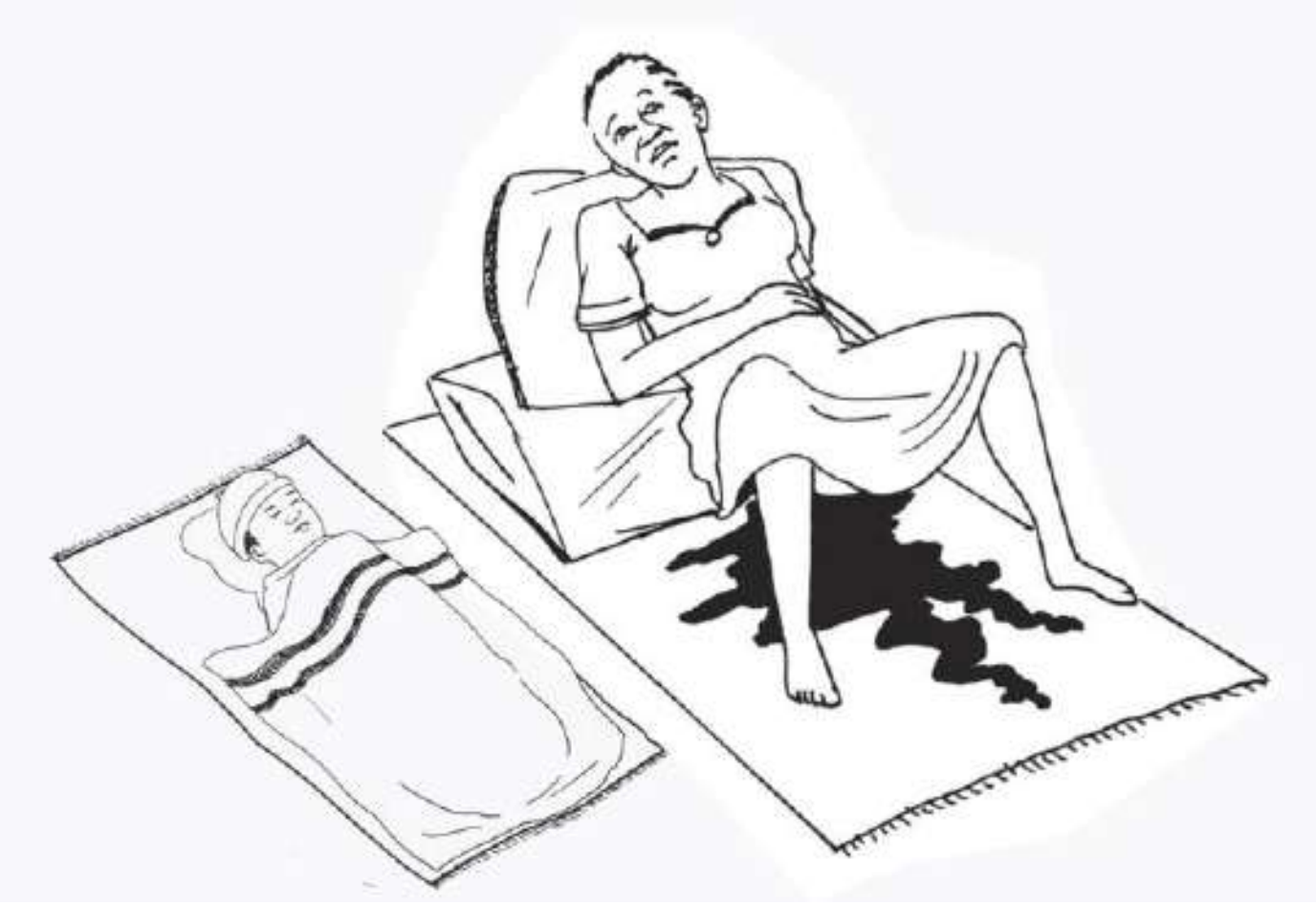

MAUMIVU MAKALI YA:
TUMBO, MATITI, KIFUA, MIGUU AU KICHWA

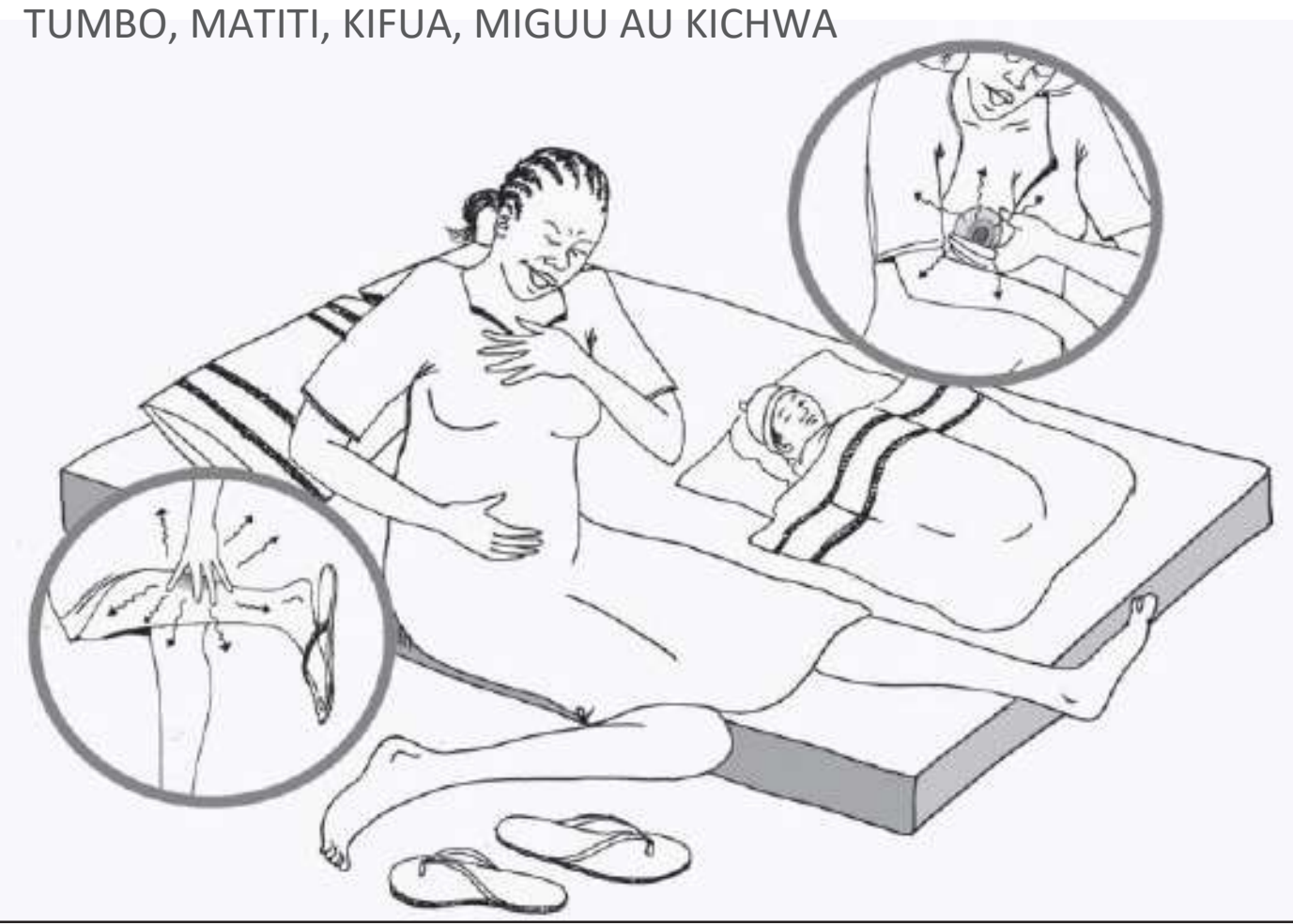

DEGEDEGE: KIFAFA CHA MIMBA

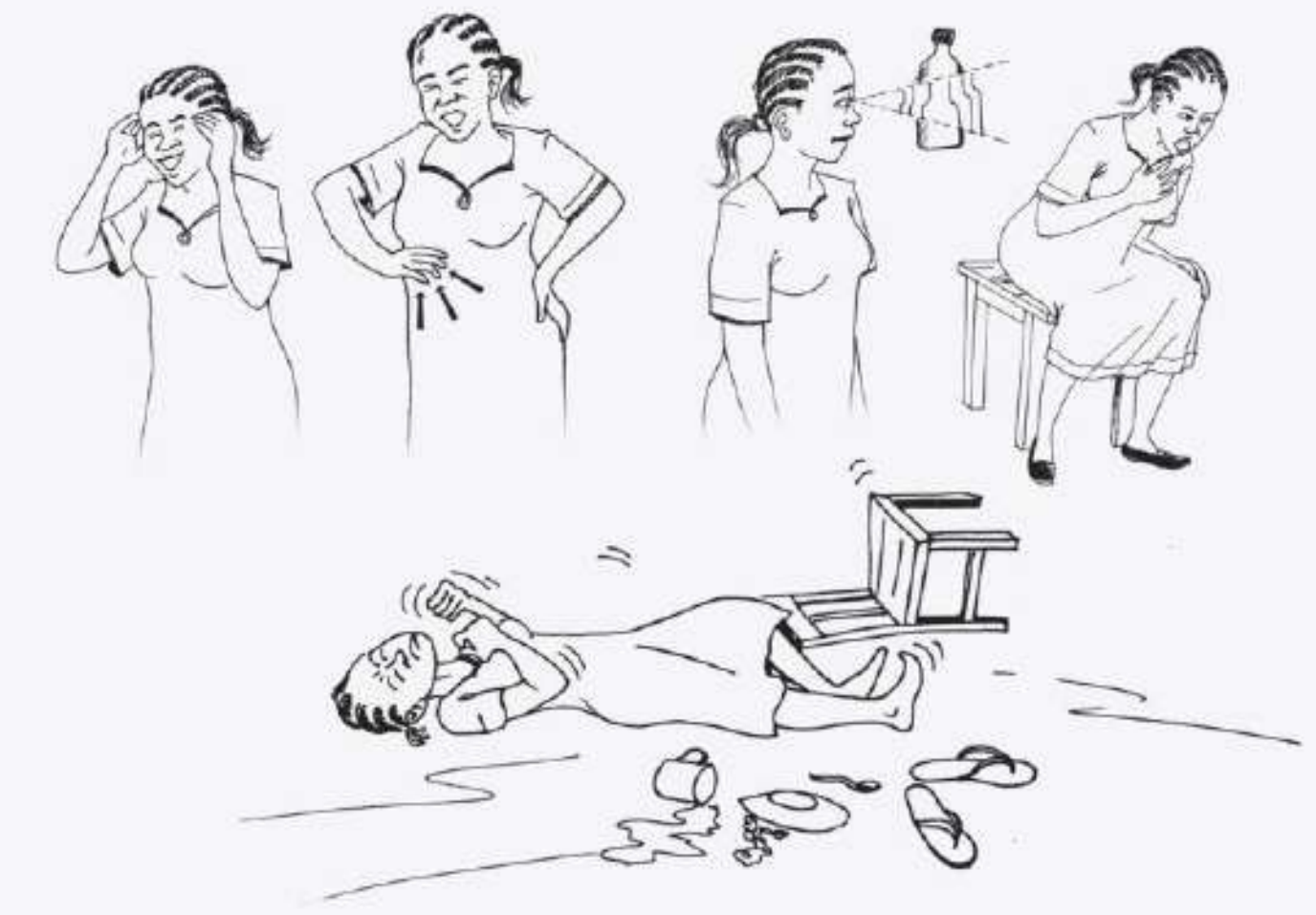

HOMA, AU KUSIKIA BARIDI, KUJIHISI MGONJWA SANA

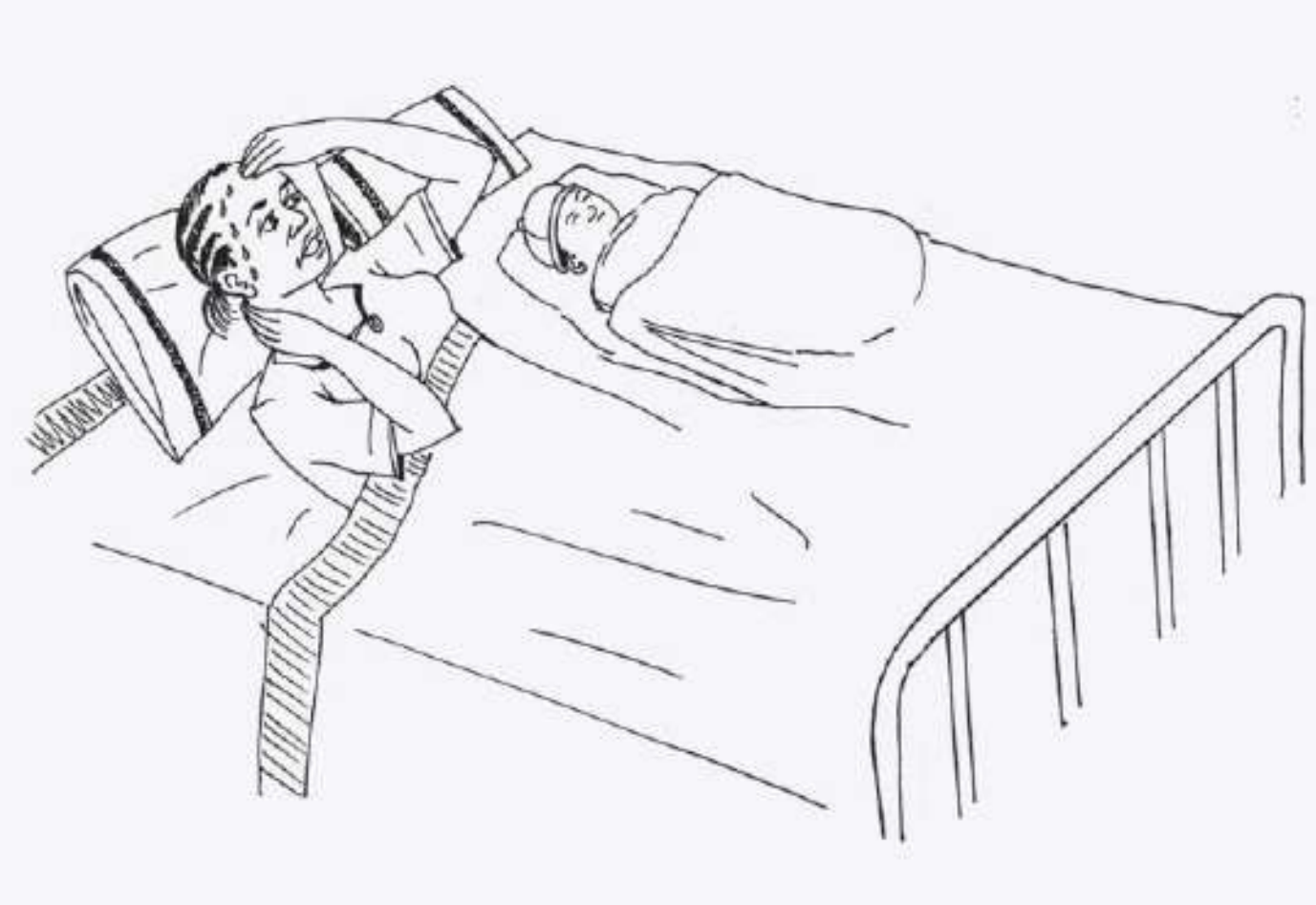

HUZUNI KUBWA SANA

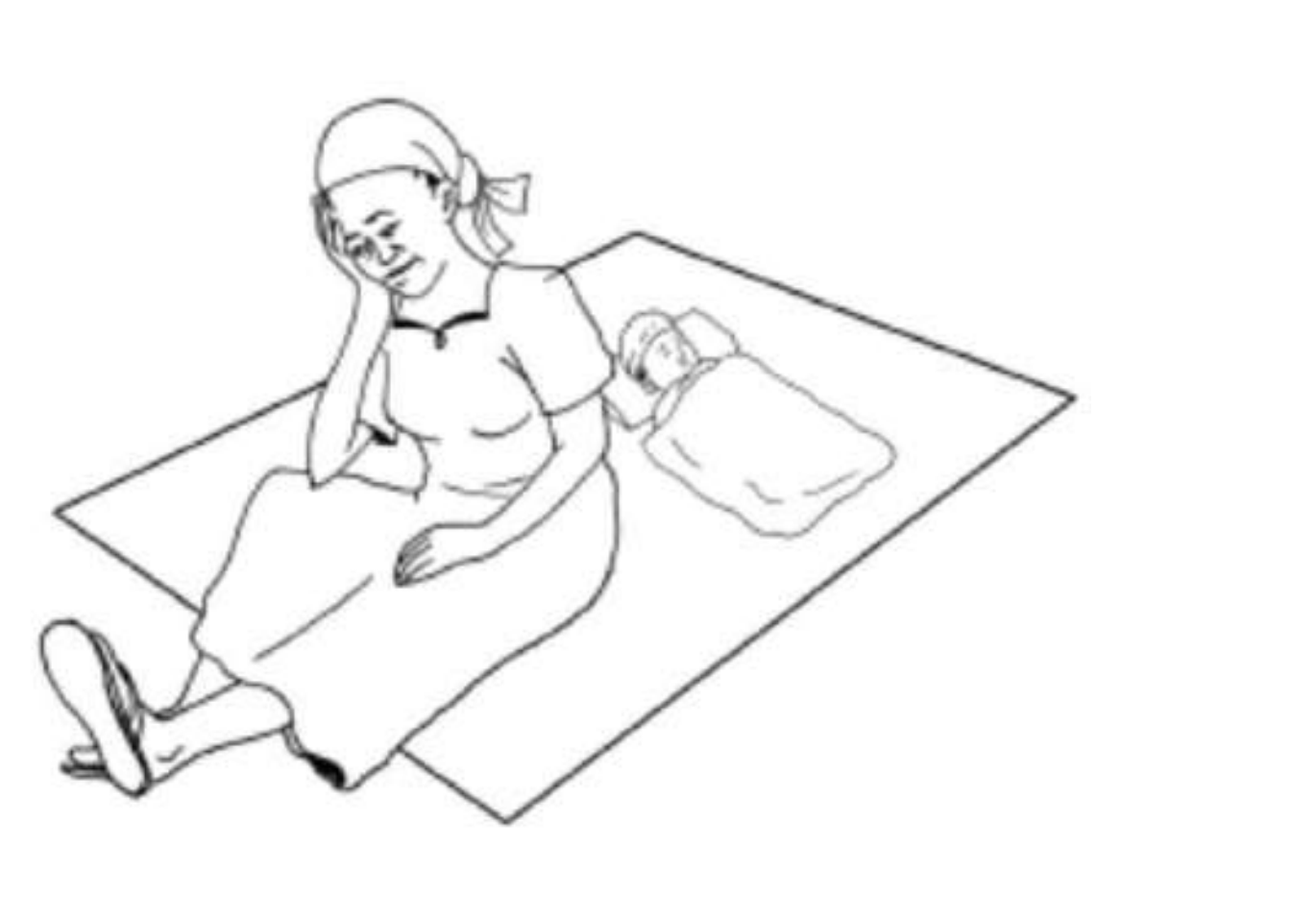

MATITI YANA WEKUNDU
AU YANA JOTO SANA

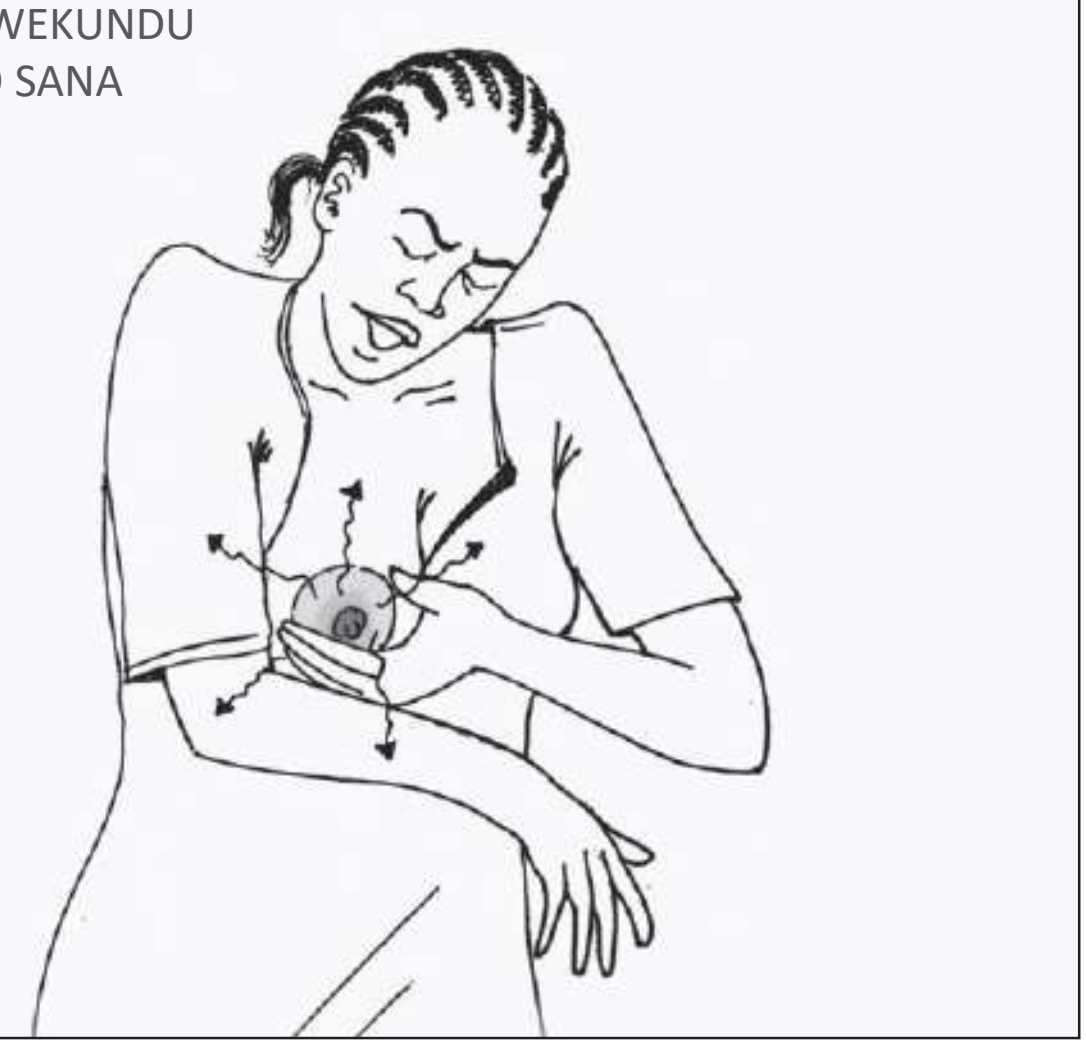

Utayari kwa ajili ya kupata mtoto/ KUJIFUNGUA!

☐ Kituo cha kujifungulia kimetambuliwa

☐ Usafiri uko tayari

☐ Wasaidizi wamechaguliwa

☐ Kiasi cha hela kimehifadhiwa

☐ Vifaa vya kujifungulia vipo tayari

☐ Uzazi wa mpango

☐ Mpango wa huduma baada ya kujifungua

☐ Maadalizi ya nguo zenye joto kwa ajili ya mtoto

☐ Tambua daliliza hatari na jinsi ya kuzuia matatizo

☐ Jina la mtoto limechaguliwa

☐ Nguo za kuvaa mwanzoni zipo tayari

☐ Familia iko tayari kumpokea mtoto

NJIA ZA KUPUNGUZA MAUMIVU WAKATI WA UCHUNGU

Unaweza kutumia njia hizi 5 kupunguza maumivu wakati wa uchungu

Pumua

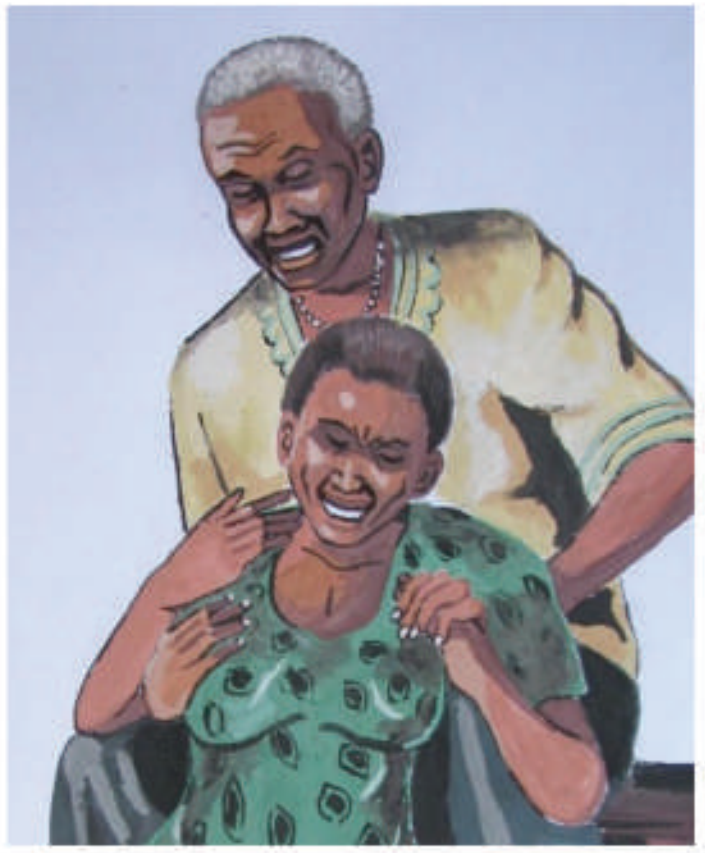

Taratibu(wakati wa kuanza uchungu) Haraka (Uchungu unapoongezeka)

Kutia moyo

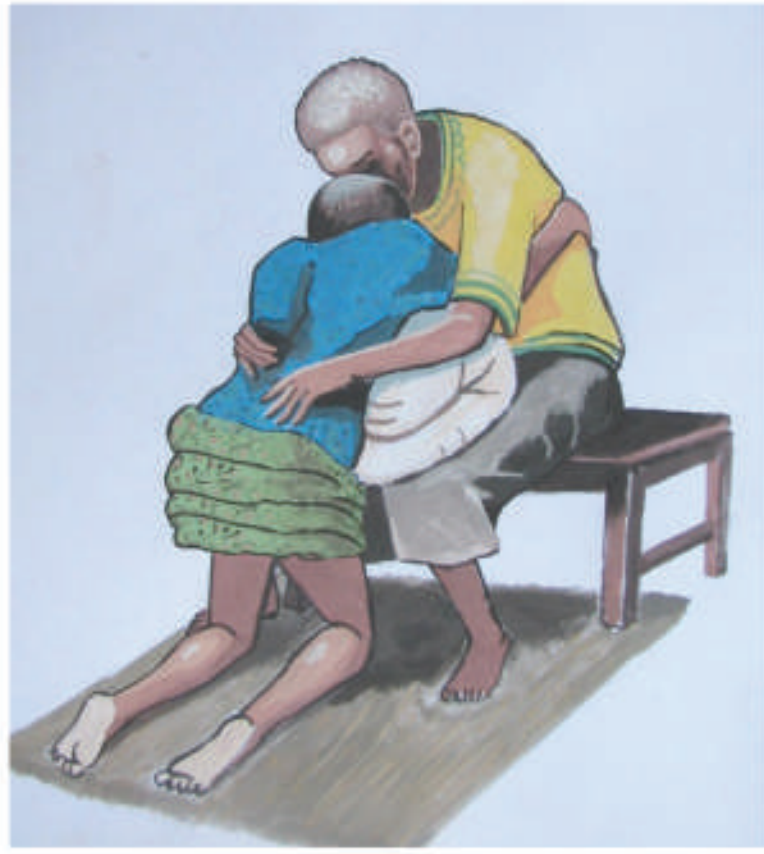

Msifie na kumpa uakika mjamzito

Zunguka

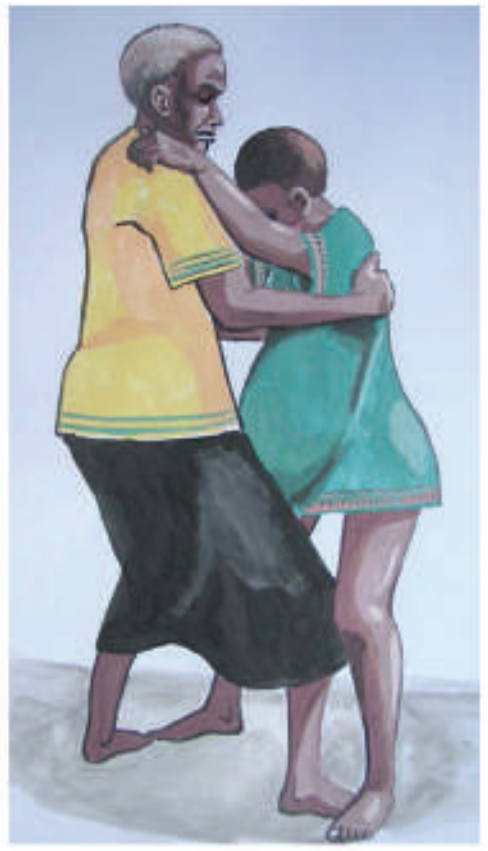

Badili mkao, tembea Tembea

Kumbuka

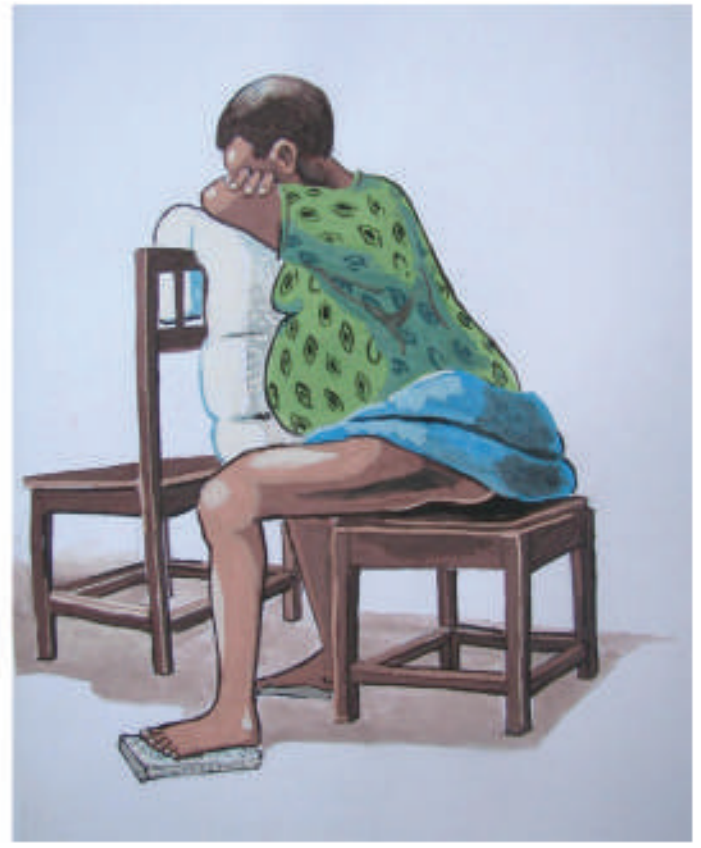

Kuhusu furaha, mawazo chanya

Kusugua

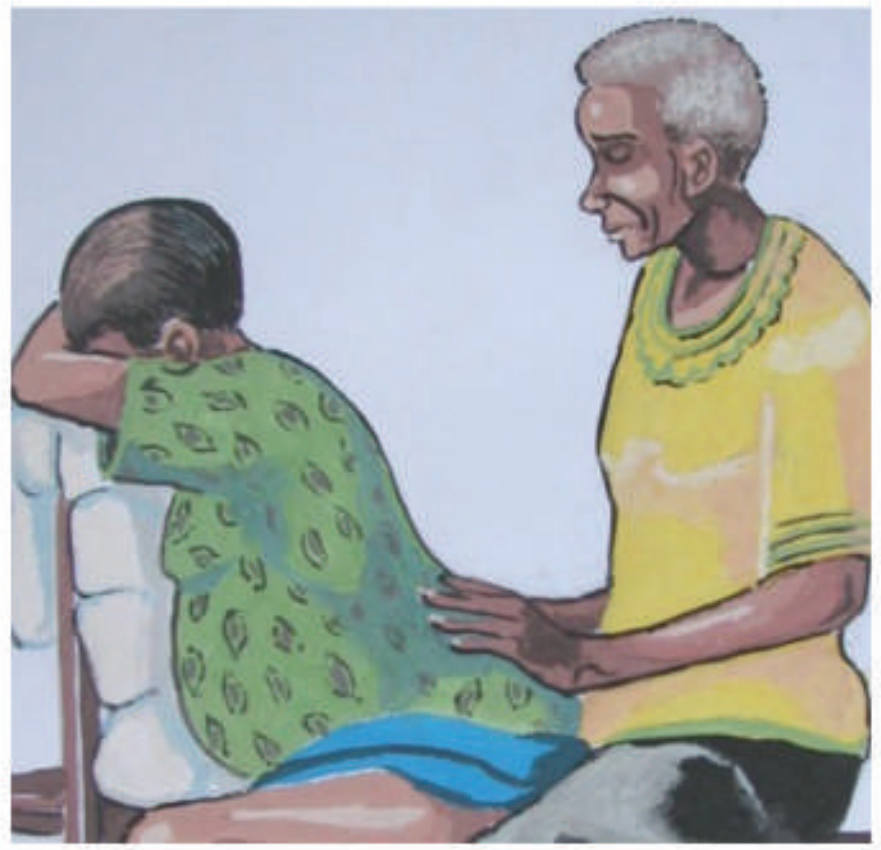

Kwenye mgongo wa mama au miguu kwa kutumia mikono au mpira

Kupunguza maumivu umsaidia mama kuhisi uzoefu mzuri wa kujali. Inaweza kusaidia hali bora wakati wa kujifungua na kwa mtoto

Pictures provided by Jill Moloney, PHC, GOAL Uganda. Ugandan artist Okello Victor
