# Supplementary material for: Implementation factors of group antenatal care in low-resource settings in Tanzania: A qualitative study using the Consolidated Framework for Implementation Research
Source: PLOS Glob Public Health. 2026 Sep 18;6(9):e0006221. doi: 10.1371/journal.pgph.0006221 (PMC13588383; doi:10.1371/journal.pgph.0006221)
Supplement: Supplementary file 5 [file pgph.0006221.s006.docx]

EDITORIAL COMMENTS

1. **Please amend your detailed Financial Disclosure statement. This is published with the article. It must therefore be completed in full sentences and contain the exact wording you wish to be published.**

*Response: We thank the editorial team for this guidance. In response, the Financial Disclosure statement has been revised accordingly.*

2. **Some material included in your submission may be copyrighted. According to PLOS’s copyright policy, authors who use figures or other material (e.g., graphics, clipart, maps) from another author or copyright holder must demonstrate or obtain permission to publish this material under the Creative Commons Attribution 4.0 International (CC BY 4.0) License used by PLOS journals. Please closely review the details of PLOS’s copyright requirements here: PLOS Licenses and Copyright. If you need to request permissions from a copyright holder, you may use PLOS's Copyright Content Permission form.**

*We thank the editorial team for this important clarification regarding copyright and licensing requirements. We carefully reviewed all submitted figures and supplementary materials for copyright compliance. Figures 1, 2, and supplementary materials S2_GANC Cohort Tracker, S3_Self Assessment Card, and S4_GANC_Women Booklet_kisw were partly developed or revised by the study team specifically for implementation of the G-ANC intervention.*

*As indicated in the submission portal, Figure 2 was adapted and modified from materials published under the Creative Commons Attribution 4.0 International (CC BY 4.0) License, with appropriate attribution maintained. The Swahili version of S4_GANC_Women Booklet_kisw and Figure 1 most illustrations (s4) were designed or edited or modified by the corresponding author (AK) with permission and part of the original developer team (Jhpiego). A similar process was followed for S2_GANC Cohort Tracker.*

*In this regard, we confirm that all materials and figures identified as potential copyright items are compatible with the Creative Commons Attribution 4.0 International (CC BY 4.0) License. The authors grant permission for these materials to be reused, adapted, and distributed in accordance with the terms of the CC BY 4.0 license, with appropriate attribution to the original sources and authors.*

***Where applicable, relevant source has been acknowledged appropriately.***

***(b) LOGOS on S2 and S4 documents***

**Response***: All logos have been removed*

**3. Please ensure that your Ethics Statement is available in its entirety at the beginning of your Methods section, under a subheading 'Ethics Statement'.**

**Response***: We thank the editorial team for this guidance. In response, the Ethics Statement has been revised and moved to the beginning of the Methods section under the subheading “Ethics Statement.*

**REVIEWER 1: COMMENTS AND RESPONSES FROM THE AUTHORS**

**Reviewer #1: First, I would like to state that I have no competing interests for this review. When appraising the quality of the article using the JBI Quality Research Synthesis tool, there is evidence of congruity among the authors' philosophical perspectives, research methodology, data analysis, and interpretation. As well as important and highly relevant cultural awareness and knowledge to better interpret the results. It would be a great added value if the authors addressed a possible influence of the research on the researchers.**

**As a reviewer, I congratulate the authors for presenting their research on a topic that requires holistic, multilevel interventions from policymakers and public health institutions, especially in contexts of high vulnerability.**

***Response****: We sincerely thank the reviewer for the positive and constructive feedback. We appreciate the reviewer’s encouraging remarks and acknowledgement of the importance of this work in strengthening maternal and newborn health services in vulnerable settings. In response to the comment, we have further clarified the reflexive role of the research team and measures taken to minimize potential bias during the research process.*

**- In the abstract, the results need more clarity on the implementation processes for advanced vs. weak planning (what is the planning referring to?) and on "champions supported delivery".**

***Response****: We thank the reviewer for this important observation. The abstract has been revised to improve clarity regarding the implementation processes. Specifically, advance planning as” a process” has been briefly clarified to refer to preparation related of specific schedules for G-ANC sessions. In addition, the description of “champions” has been clarified to refer to CHWs and HCPs with positive in group care who supported implementation.*

**- In the introduction, the acronyms G-ANC, GA, and CFIR are introduced before their explanation. Even though it refers to known acronyms, it is recommended that the authors state them.**

**Response***: Thank you for this important observation. Previously, the authors considered the prior clarification of these acronyms in the abstract to be sufficient. However, we have carefully considered the reviewer’s comment, and the manuscript has now been revised to ensure that all acronyms are fully stated at their first appearance in the introduction.*

**- In the methods, the authors are recommended to clarify about the pilot study "Mlinde Mama project" given the impact of said implementation on the subsequent data collection, please also cite, if available.**

**Response***: We thank the reviewer for this important suggestion. The Methods section has been revised to provide additional clarification on the Mlinde Mama project. Relevant details on the implementation period and context have been added, and a citation describing the project outcomes has also been included in the manuscript.*

*The Mlinde Mama Project, named after Kiswahili words meaning “protect a mother,” was implemented by Prime Health Initiative Tanzania (PHIT) with funding support from the Bill and Melinda Gates Foundation (BMGF). Published findings from the project indicate that G-ANC contributed to improved ANC coverage and increased uptake of selected ANC services, including malaria testing, blood pressure monitoring, and preventive medications (19). Women participating in G-ANC also reported positive experiences of care, increased satisfaction, and enhanced emotional and peer support (19,31).*

**- In the methods, the authors are recommended to specify the eligibility criteria for pregnant women to join the group antenatal care.**

***Response****: Thank you for this important observation. The Methods section has been revised to further clarify the eligibility criteria for participation in G-ANC. Specifically,* *we included women with GA below 20 weeks, willingness to participate in group sessions, and without serious mental health conditions or severe medical illnesses such as chronic hypertension.*

**- In the methods, it is mentioned that women measured their own blood pressure and weight. Did the women receive any training for this measurements, mainly blood pressure? And if not, have the authors considered the possibility that this could introduce bias in the measurement, and whether it is worth mentioning for the authors, given that it was found to be a barrier within the first domain of "Intervention characteristics"?**

***Response****: This is an important observation and the section has been revised accordingly.* *Women participating in G-ANC received orientation and practical guidance from HCPs on how to measure blood pressure and weight before conducting self-assessments during group sessions (This sentence was added on description section). In addition, all self-recorded measurements were reviewed and confirmed by healthcare providers during the individual clinical consultation. We agree that for some women, confidence with self-assessment could potentially affect consistency of measurements, and this has been acknowledged in the manuscript as part of the implementation challenges.*

**- In the methods, are the interview guides used by the authors previously validated, or are they only "informed by exisiting similar studies"? Given the relevance of these guides to data collection, the authors should state any major changes or adaptations from validated questionnaires.**

**Response***: We thank the reviewer for this important comment. This comment has been addressed clearly; The interview guides were not adopted as fully validated instruments but were revised based on existing studies on G-ANC and informed by the CFIR framework.* *The interview guides were piloted to assess clarity, relevance, flow of questions, and contextual appropriateness. The pilot process also helped refine wording, sequencing of probes, and interview approaches to improve consistency and depth of data collection across participant groups. These clarifications have now been added to the Methods section.*

**- Does the software used for audio recording during the interviews require a license? The authors are recommended to address this concern to avoid ethical issues.**

**Response***: Thanks for this valid comment. The authors addressed revised this accordingly; Audio recordings were conducted using password-protected digital recording devices and secure mobile applications approved for research data collection. The software used did not require any authorized or licensed access. This clarification has been added to the Methods section.*

**- The first paragraph of the data analysis is confusing to read, as if it were missing information.**

**Response***: We thank the reviewer for this observation. In response to this comment and related feedback from the second reviewer, we comprehensively revised the analysis section to improve clarity, precision, and overall coherence.*

**- It is not clear whether this study presents the results of the pilot implementation phase of the intervention (Group Antenatal Care) or the results after the intervention was fully implemented in the selected facilities, given the timelines presented: "The G-ANC pilot implementation was conducted between January 2023 and June 2024. However, a qualitative data collection conducted between June and November 2024."**

**Response***: The intervention was fully implemented from January 2023 to June 2024, followed by an evaluation phase conducted between June and November 2024. These results are after the intervention was fully implemented. To avoid confusion, relevant terms have been revised throughout the manuscript.*

**- It is recommended that the tables and the figures can "stand alone" through their descriptions and titles, including the specification of acronyms.**

**Response***: We thank the reviewer for this important observation. In response to comments from both reviewers, the results section has been comprehensively revised to improve clarity, synthesis, and interpretability. Although the narratives are organized according to specific CFIR domains, they were revised to function as self-explanatory presentations by clearly specifying facilitators, barriers, and supporting participant quotations. This was done to ensure that readers can understand the key findings without relying heavily on the accompanying tables.*

*In addition, we revised the presentation of the findings to improve prioritization by emphasizing the factors most consistently reported across participants and settings. Following the richness of the data and the complexity of the implementation approach, we retained the tables to ensure comprehensive presentation of the findings. The tables were retained primarily to support transparency and present additional constructs and illustrative quotations, while the narrative now focuses more clearly on synthesis, interpretation, and emerging patterns across domains.*

**- It is recommended to maintain consistent wording throughout the study, particularly regarding acronyms and the use of full names.**

**Response***: We thank the reviewer for this observation. In response, the manuscript has been carefully reviewed and revised to ensure consistency in wording throughout the study, particularly in the use of acronyms, terminology, and full names across all sections. Acronyms were used consistently after their first definition, while full names were maintained when beginning a sentence. The most commonly retained full terms were healthcare providers (HCPs) and, in selected paragraphs, Group Antenatal Care (G-ANC) to improve readability and sentence flow. In addition, the full term “healthcare providers” was maintained within participant quotations presented only in the tables.*

**- It is recommended to check for grammatical errors and consistent orthography throughout the study.**

**Response***: Thank you for your valuable comments. The full manuscript has been carefully reviewed to ensure consistent orthography, grammar, and academic writing style throughout. Some of the key revisions made include:*

1. *Addition of missing articles, particularly “the” and “a.”*
2. *Correction of sentence structure and subject–verb agreement to improve readability (e.g., “this focuses” instead of “this focus”).*
3. *Maintenance of tense consistency throughout the manuscript (e.g., “studies have highlighted” instead of “studies highlighted”).*
4. *Improvement of academic phrasing and clarity (e.g., “well-structured” instead of “good structured”).*
5. *Revision of wording for clarity and precision (e.g., “effectiveness” instead of “effect” where appropriate).*
6. *Correction of punctuation inconsistencies. Eg. Colon before quotations*
7. *Revision of paragraph structure and coherence to improve flow and readability.*
8. *Consistent use of “G-ANC” throughout the manuscript instead of interchangeably using “group care.”*
9. *Maintenance of a uniform English standard throughout the manuscript (e.g., “analyse” versus “analyze”).*
10. *Improvement of word forms and terminology accuracy (e.g., “adapted” instead of “adopted”).*
11. *Correction of spelling and formatting inconsistencies (e.g., “in-depth” instead of “indepth”).*

**REVIEWER 2: COMMENTS AND RESPONSES FROM THE AUTHORS**

**Comment: The manuscript claims that “no study in similar settings has applied formal implementation science frameworks” to G-ANC. This is likely overstated.**

**Response***: We thank the reviewer for this important observation. We agree that the original statement overstated the novelty of the study. The manuscript has been revised .The revised text now emphasizes the limited application of CFIR within the Tanzanian context.*

**Comment: Clarify what is uniquely added (e.g., multi-level CFIR mapping? rural facility diversity? policy implications?).**

**Response***: We thank the reviewer for this suggestion. The manuscript has been revised to further clarify the unique contribution of this study, including the application of CFIR to and its implication within routine maternal health services in low-resource settings (The last two paragraphs)*

**Comment: Snowball sampling is mentioned but not justified.**

**Response***: We thank the reviewer for this observation. We have reviewed the manuscript has to clarify that snowball sampling was specifically used to identify women who had previously participated in G-ANC but were no longer attending ANC clinics, in order to capture a broader range of implementation experiences beyond currently active participants.*

**Comment: It is unclear how initial participants were identified.**

**Response***: We thank the reviewer for this observation. Additional clarification has been added to explain that initial participants were identified through ANC registers, G-ANC cohort tracking tools, and support from healthcare providers involved in ANC services. Also, we have explained how it was done and how other participants were approached (****on selection of the participants paragraph 2)***

**Comment: Whether selection bias may have favored more engaged G-ANC participants.**

**Response***: We appreciate the reviewer’s important observation. The possibility that women with more positive or sustained engagement in G-ANC may have been more likely to participate has been acknowledged and added in the limitations section as a potential selection bias.*  **(Paragraph 3 in Strengths and Limitations section)**

**Comment: Clarify inclusion/exclusion criteria.**

**Response***: We thank the reviewer for this suggestion. The manuscript has been revised to further clarify eligibility criteria for women participating in G-ANC, including gestational age below 20 weeks, willingness to participate in group sessions, and absence of serious mental health conditions or severe medical illnesses.*

**Included revision in manuscript:**

*Women eligible for G-ANC included those with gestational age below 20 weeks, willingness to participate in group sessions, and without serious mental health conditions or severe medical illnesses.*

**Comment: Non-participation/refusal rates (if available).**

**Response***: We thank the reviewer for this suggestion. Information on non-participation was limited because recruitment occurred within routine service settings and participation was voluntary (Participation refusal was not documented)*

**Comment: The manuscript states saturation was achieved but does not explain how it was assessed operationally.**

**Response***: We thank the reviewer for this important observation. The manuscript has been revised to clarify that saturation was assessed iteratively during ongoing data collection and analysis and was considered achieved when additional interviews and discussions no longer generated substantively new insights related to implementation experiences.*

**Comment: Whether saturation was tracked by stakeholder group.**

**Response***: We appreciate the reviewer’s suggestion. The manuscript now clarifies that saturation was assessed both within and across stakeholder groups* *to ensure adequate representation of perspectives from women, healthcare providers, and health managers.*

**Comment: The “hybrid deductive–inductive approach” is mentioned but poorly described.**

**Missing details on coding process.**

**Missing number of coders and consensus process.**

**Missing use of software.**

**Response***: We thank the reviewer for this observation. In response to this comment and related feedback from the first reviewer, we comprehensively revised the analysis section to improve clarity, precision, and overall coherence.*

**The results rely heavily on tables and the narrative - Often repeats table content rather than synthesizing insights and Lacks prioritization (all factors appear equally important).**

**Response***: We thank the reviewer for this important observation. In response to comments from both reviewers, the results section has been comprehensively revised to improve clarity, synthesis, and interpretability. Although the narratives are organized according to specific CFIR domains, they were revised to function as self-explanatory presentations by clearly specifying facilitators, barriers, and supporting participant quotations. This was done to ensure that readers can understand the key findings without relying heavily on the accompanying tables.*

*In addition, we revised the presentation of the findings to improve prioritization by emphasizing the factors most consistently reported across participants and settings. Following the richness of the data and the complexity of the implementation approach, we retained the tables to ensure comprehensive presentation of the findings. The tables were retained primarily to support transparency and present additional constructs and illustrative quotations, while the narrative now focuses more clearly on synthesis, interpretation, and emerging patterns across domains.*

**Policy recommendations need to be expanded in terms of a) staffing, b) space and c) integration in ANC guidelines.**

**Response***: We thank the reviewer for this important recommendation. In response, the implications section has been expanded to more clearly address key system-level considerations for sustainable implementation of G-ANC. Specifically, we strengthened discussion on the need for adequate staffing and workload redistribution to support continuity of group sessions. We stressed the need to address the above issues for successful integration of G-ANC into routine ANC guidelines.*

**Limitations section can be further strengthened around selection bias, positive reporting bias and non-participation perspectives.**

**Response***: We thank the reviewer for this important observation. The Strengths and Limitations section has been revised to further acknowledge potential selection bias, positive reporting bias, and limited representation of perspectives from women who may have discontinued participation or declined involvement in the study.*

***Included revision in manuscript:***

*Women with more positive experiences or sustained participation in G-ANC may have been more likely to participate in the study because they were easier to identify through ongoing clinic attendance and more willing to share their experiences. Potentially, this may introduce selection and positive reporting bias. Although snowball sampling was used to identify postpartum women who were no longer actively attending ANC clinics, perspectives from women who completely disengaged from care or declined participation may remain underrepresented. Despite these limitations, the study provides in-depth, contextually grounded insights that are valuable for informing adaptation and strengthening of G-ANC implementation in similar low-resource settings.*

**While the study has clear potential for publication, but requires stronger analytical depth, clearer methods description, and more critical interpretation to meet the standards for PLOS Global Public Health.**

**Response***: We thank both reviewers for their constructive assessment and valuable recommendations. In response, the manuscript has been comprehensively revised to strengthen the analytical depth, improve clarity of the methods description and provide more critical interpretation of the findings. The discussion section was also expanded to provide deeper interpretation of the findings in relation to existing literature and implications for policy, practice, and future research. These revisions have improved the overall novelty, rigor and clarity.*
