# Supplementary material for: Implementation factors of group antenatal care in low-resource settings in Tanzania: A qualitative study using the Consolidated Framework for Implementation Research
Source: PLOS Glob Public Health. 2026 Sep 18;6(9):e0006221. doi: 10.1371/journal.pgph.0006221 (PMC13588383; doi:10.1371/journal.pgph.0006221)
Supplement: Peer review history

# Peer review: Decision Letter 0

**DOI:** 10.1371/journal.pgph.0006221.r001

**Authors:** Ferdinand Mukumbang

10 May 2026

PGPH-D-26-00997

Implementation factors of Group Antenatal Care in Low-Resource settings in Tanzania: a Qualitative Study using the Consolidated Framework for Implementation Research

PLOS Global Public Health

Dear Dr. Kinyina,

Thank you for submitting your manuscript to PLOS Global Public Health. After careful consideration, we feel that it has merit but does not fully meet PLOS Global Public Health’s publication criteria as it currently stands. Therefore, we invite you to submit a revised version of the manuscript that addresses the points raised during the review process.

Two reviewers have reviewed this manuscript and have provided valuable feedback for the authors to consider. They have raised concerns around the methodology and reporting of findings. Kindly pay attention to addressing these and all the other issues that they have brought to your attention.

Please submit your revised manuscript by June 30, 2026. If you will need more time than this to complete your revisions, please reply to this message or contact the journal office at globalpubhealth@plos.org. When you're ready to submit your revision, log on to https://www.editorialmanager.com/pgph/ and select the 'Submissions Needing Revision' folder to locate your manuscript file.

Please include the following items when submitting your revised manuscript:

- A letter that responds to each point raised by the editor and reviewer(s). You should upload this letter as a separate file labeled 'Response to Reviewers'.
- A marked-up copy of your manuscript that highlights changes made to the original version. You should upload this as a separate file labeled 'Revised Manuscript with Track Changes'.
- An unmarked version of your revised paper without tracked changes. You should upload this as a separate file labeled 'Manuscript'.

Guidelines for resubmitting your figure files are available below the reviewer comments at the end of this letter.

As the corresponding author, your ORCID iD is verified in the submission system and will appear in the published article. PLOS supports the use of ORCID, and we encourage all coauthors to register for an ORCID iD and use it as well. Please encourage your coauthors to verify their ORCID iD within the submission system before final acceptance, as unverified ORCID iDs will not appear in the published article. *Only*  the individual author can complete the verification step; PLOS staff *cannot*  verify ORCID iDs on behalf of authors.

We look forward to receiving your revised manuscript.

Kind regards,

Ferdinand C Mukumbang, PhD

Academic Editor

PLOS Global Public Health

**Journal Requirements:**

1. Please amend your detailed Financial Disclosure statement. This is published with the article. It must therefore be completed in full sentences and contain the exact wording you wish to be published.

i. Please clarify all sources of financial support for your study. List the grants, grant numbers, and organizations that funded your study, including funding received from your institution. Please note that suppliers of material support, including research materials, should be recognized in the Acknowledgements section rather than in the Financial Disclosure.

ii. State the initials, alongside each funding source, of each author to receive each grant. For example: "This work was supported by the National Institutes of Health (####### to AM; ###### to CJ) and the National Science Foundation (###### to AM)."

iii. State what role the funders took in the study. If the funders had no role in your study, please state: “The funders had no role in study design, data collection and analysis, decision to publish, or preparation of the manuscript.”

iv. If any authors received a salary from any of your funders, please state which authors and which funders.

2. Some material included in your submission may be copyrighted. According to PLOS’s copyright policy, authors who use figures or other material (e.g., graphics, clipart, maps) from another author or copyright holder must demonstrate or obtain permission to publish this material under the Creative Commons Attribution 4.0 International (CC BY 4.0) License used by PLOS journals. Please closely review the details of PLOS’s copyright requirements here: PLOS Licenses and Copyright. If you need to request permissions from a copyright holder, you may use PLOS's Copyright Content Permission form.

Please respond directly to this email or email the journal office and provide any known details concerning your material's license terms and permissions required for reuse, even if you have not yet obtained copyright permissions or are unsure of your material's copyright compatibility.

Potential Copyright Issues:

a. Figures 1, 2 and S2_GANC Cohort tracker, S3_Self Assessment Card, S4_GANC_Women booklet_kisw: Please confirm whether you drew the images / clip-art within the figure panels by hand. If you did not draw the images, please provide (a) a link to the source of the images or icons and their license / terms of use; or (b) written permission from the copyright holder to publish the images or icons under our CC-BY 4.0 license. Alternatively, you may replace the images with open source alternatives. See these open source resources you may use to replace images / clip-art:

- https://commons.wikimedia.org

- https://openclipart.org/

b. S2_GANC Cohort tracker, S4_GANC_Women booklet_kisw contains branding/a logo. We are not permitted to publish this under our CC-BY 4.0 license, even with permission. We ask that you please remove or replace it.

3. Please ensure that your Ethics Statement is available in its entirety at the beginning of your Methods section, under a subheading 'Ethics Statement'.

If the reviewer comments include a recommendation to cite specific previously published works, please review and evaluate these publications to determine whether they are relevant and should be cited. There is no requirement to cite these works unless the editor has indicated otherwise.

**Additional Editor Comments (if provided):**

[Note: HTML markup is below. Please do not edit.]

Reviewers' comments:

Reviewer's Responses to Questions

**Comments to the Author**

1. Does this manuscript meet PLOS Global Public Health’s publication criteria?

Reviewer #1: Yes

Reviewer #2: Yes

**********

2. Has the statistical analysis been performed appropriately and rigorously?

Reviewer #1: N/A

Reviewer #2: I don't know

**********

3. Have the authors made all data underlying the findings in their manuscript fully available (please refer to the Data Availability Statement at the start of the manuscript PDF file)?

The PLOS Data policy

Reviewer #1: Yes

Reviewer #2: Yes

**********

4. Is the manuscript presented in an intelligible fashion and written in standard English?

Reviewer #1: Yes

Reviewer #2: No

**********

**Reviewer #1:**  First, I would like to state that I have no competing interests for this review. When appraising the quality of the article using the JBI Quality Research Synthesis tool, there is evidence of congruity among the authors' philosophical perspectives, research methodology, data analysis, and interpretation. As well as important and highly relevant cultural awareness and knowledge to better interpret the results. It would be a great added value if the authors addressed a possible influence of the research on the researchers.

As a reviewer, I congratulate the authors for presenting their research on a topic that requires holistic, multilevel interventions from policymakers and public health institutions, especially in contexts of high vulnerability.

When reviewing the paper, I came across some questions and doubts that arose during the process:

- In the abstract, the results need more clarity on the implementation processes for advanced vs. weak planning (what is the planning referring to?) and on "champions supported delivery".

- In the introduction, the acronyms G-ANC, GA, and CFIR are introduced before their explanation. Even though it refers to known acronyms, it is recommended that the authors state them.

- In the methods, the authors are recommended to clarify about the pilot study "Mlinde Mama project" given the impact of said implementation on the subsequent data collection, please also cite, if available.

- In the methods, the authors are recommended to specify the eligibility criteria for pregnant women to join the group antenatal care.

- In the methods, it is mentioned that women measured their own blood pressure and weight. Did the women receive any training for this measurements, mainly blood pressure? And if not, have the authors considered the possibility that this could introduce bias in the measurement, and whether it is worth mentioning for the authors, given that it was found to be a barrier within the first domain of "Intervention characteristics"?

- In the methods, are the interview guides used by the authors previously validated, or are they only "informed by exisiting similar studies"? Given the relevance of these guides to data collection, the authors should state any major changes or adaptations from validated questionnaires.

- Does the software used for audio recording during the interviews require a license? The authors are recommended to address this concern to avoid ethical issues.

- The first paragraph of the data analysis is confusing to read, as if it were missing information.

- It is not clear whether this study presents the results of the pilot implementation phase of the intervention (Group Antenatal Care) or the results after the intervention was fully implemented in the selected facilities, given the timelines presented: "The G-ANC pilot implementation was conducted between January 2023 and June 2024. However, a qualitative data collection conducted between June and November 2024."

- It is recommended that the tables and the figures can "stand alone" through their descriptions and titles, including the specification of acronyms.

- It is recommended to maintain consistent wording throughout the study, particularly regarding acronyms and the use of full names.

- It is recommended to check for grammatical errors and consistent orthography throughout the study.

**Reviewer #2:**  The paper is generally well-structured, methodologically sound, and grounded in theory. The use of CFIR is appropriate and strengthens analytical rigor. However, several areas require clarification, tightening, and deeper critical engagement—particularly around methods transparency, analytical depth, and positioning within existing literature.

• The manuscript claims that “no study in similar settings has applied formal implementation science frameworks” to G-ANC. This is likely overstated.

o There are implementation-focused studies using frameworks (including CFIR-informed or realist approaches) in Kenya, Rwanda, and Nigeria.

• The authors should:

o Reframe the contribution more cautiously (e.g., “limited application in Tanzania” rather than globally).

o Clarify what is uniquely added (e.g., multi-level CFIR mapping? rural facility diversity? policy implications?).

Insufficient Detail for Reproducibility

a. Sampling and Recruitment

• Snowball sampling is mentioned but not justified.

• It is unclear:

o How initial participants were identified

o Whether selection bias may have favored more engaged G-ANC participants

• Clarify:

o Inclusion/exclusion criteria

o Recruitment flow

o Non-participation/refusal rates (if available)

b. Data Saturation

• The manuscript states saturation was achieved but does not explain:

o How it was assessed operationally

o Whether saturation was tracked by stakeholder group

• Suggest adding a brief methodological explanation.

c. Data Analysis

• The “hybrid deductive–inductive approach” is mentioned but poorly described.

• Missing details:

o Coding process (open → axial? framework coding?)

o Number of coders

o Inter-coder reliability or consensus process

o Use of software (e.g., NVivo, Atlas.ti)

This is a key weakness—analysis currently reads as descriptive rather than analytically transparent.

The results rely heavily on tables and the narrative - Often repeats table content rather than synthesizing insights and Lacks prioritization (all factors appear equally important).

The discussion needs to be stronger. I would particularly like the authors to explore further the tension between efficiency and workload; and further work on the equity implicaitions, particularly the socioeconomic barriers. Also, the privacy and peer support trade-off, while important, needs further discussion.

Policy recommendations need to be expanded in terms of a) staffing, b) space and c) integration in ANC guidelines.

Limitations section can be further strengthened around selection bias, positive reporting bias and non-participation perspectives.

While the study has clear potential for publication, but requires stronger analytical depth, clearer methods description, and more critical interpretation to meet the standards for PLOS Global Public Health.

**********

what does this mean?). If published, this will include your full peer review and any attached files.

**Do you want your identity to be public for this peer review?**  If you choose “no”, your identity will remain anonymous but your review may still be made public.

For information about this choice, including consent withdrawal, please see our Privacy Policy

Reviewer #1: **Yes:**  Maira Alejandra Vera Montoya

Reviewer #2: No

**********

[NOTE: If reviewer comments were submitted as an attachment file, they will be attached to this email and accessible via the submission site. Please log into your account, locate the manuscript record, and check for the action link "View Attachments". If this link does not appear, there are no attachment files.]

**Figure Resubmissions:**

# Author comment: Author response to Decision Letter 1

**DOI:** 10.1371/journal.pgph.0006221.r002

1 Jun 2026

Attachment Submitted filename: Response to Reviewers.docx

# Peer review: Decision Letter 1

**DOI:** 10.1371/journal.pgph.0006221.r003

**Authors:** Ferdinand Mukumbang

24 Jul 2026

Implementation factors of Group Antenatal Care in Low-Resource settings in Tanzania: a Qualitative Study using the Consolidated Framework for Implementation Research

PGPH-D-26-00997R1

Dear Dr Kinyina

We are pleased to inform you that your manuscript 'Implementation factors of Group Antenatal Care in Low-Resource settings in Tanzania: a Qualitative Study using the Consolidated Framework for Implementation Research' has been provisionally accepted for publication in PLOS Global Public Health.

Before your manuscript can be formally accepted you will need to complete some formatting changes, which you will receive in a follow up email. A member of our team will be in touch with a set of requests.

Please note that your manuscript will not be scheduled for publication until you have made the required changes, so a swift response is appreciated.

IMPORTANT: The editorial review process is now complete. PLOS will only permit corrections to spelling, formatting or significant scientific errors from this point onwards. Requests for major changes, or any which affect the scientific understanding of your work, will cause delays to the publication date of your manuscript.

If your institution or institutions have a press office, please notify them about your upcoming paper to help maximize its impact. If they'll be preparing press materials, please inform our press team as soon as possible -- no later than 48 hours after receiving the formal acceptance. Your manuscript will remain under strict press embargo until 2 pm Eastern Time on the date of publication. For more information, please contact globalpubhealth@plos.org.

Thank you again for supporting Open Access publishing; we are looking forward to publishing your work in PLOS Global Public Health.

Best regards,

Ferdinand C Mukumbang, PhD

Academic Editor

PLOS Global Public Health

***********************************************************

Reviewer Comments (if any, and for reference):

Reviewer's Responses to Questions

**Comments to the Author**

Reviewer #1: All comments have been addressed

**********

publication criteria?

Reviewer #1: Yes

**********

3. Has the statistical analysis been performed appropriately and rigorously?

Reviewer #1: N/A

**********

4. Have the authors made all data underlying the findings in their manuscript fully available (please refer to the Data Availability Statement at the start of the manuscript PDF file)?

The PLOS Data policy

Reviewer #1: Yes

**********

5. Is the manuscript presented in an intelligible fashion and written in standard English?

Reviewer #1: Yes

**********

Reviewer #1: The authors answered and considered the questions and suggestions presented on the first revision. The manuscript is presented in a clear and structured way that allows the comprehension of the research question, as well as the social and public health issue that this question addresses.

**********

what does this mean?). If published, this will include your full peer review and any attached files.

**Do you want your identity to be public for this peer review?**  If you choose “no”, your identity will remain anonymous but your review may still be made public.

For information about this choice, including consent withdrawal, please see our Privacy Policy

Reviewer #1: **Yes:**  Maira Alejandra Vera Montoya

**********
